# The role of speaker accuracy and mutual exclusivity in children’s novel word learning

**DOI:** 10.1371/journal.pone.0361483

**Published:** 2026-10-29

**Authors:** Shalini Banerjee, Ishanti Gangopadhyay

**Affiliations:** Department of Speech, Language and Hearing Sciences, Indiana University, Bloomington, Indiana, United States of America; Education University of Hong Kong, HONG KONG

## Abstract

Children draw on multiple sources of information during word learning, including speaker testimony and lexical expectations. Yet, little is known about how these cues jointly influence children’s ability to learn new words. The present study examined the combined effect of speaker accuracy and mutual exclusivity (ME) on short-term retention of label-object associations in 4- to 7-year-old monolingual English speaking children. Children were presented novel and familiar object pairs, and were taught novel labels by either an accurate speaker (one who previously labeled familiar objects correctly) or an inaccurate speaker (one who previously labeled familiar objects incorrectly). Novel labels were introduced under two conditions: Align-with-ME (speakers labeled the novel object with novel names) and Conflict-with-ME (speakers labeled the familiar object with novel names, conflicting with ME). Results showed that children retained novel labels for novel objects from both accurate and inaccurate speakers when speaker testimony aligned with ME expectations. This suggests that children are open to learning novel words from speakers regardless of their prior accuracy and may also draw on other available cues, such as label–object co-occurrences, during learning. However, when speaker testimony violated ME expectations, children performed poorly, possibly due to difficulty disambiguating between speaker testimony and their existing lexical knowledge. Together, these findings suggest that children’s short-term retention of label–object associations is supported when available cues align, irrespective of speaker accuracy status. These associations, however, become less robust when ME and speaker cues provide conflicting information.

## Introduction

Children learn words rapidly during the first few years of life by integrating multiple sources of information, including social cues provided by speakers and expectations derived from their existing lexical knowledge [1–5]. Two cues that have received considerable attention in the word-learning literature are speaker accuracy and the mutual exclusivity (ME) principle. Speaker accuracy reflects children’s evaluation of the reliability of information provided by others and is typically established through a speaker’s prior use of known object labels. Studies examining speaker accuracy have reported mixed findings [6–9], with some showing that children preferentially seek and accept information from speakers who have previously labeled objects accurately [8–13], whereas others have found that children can learn and recall novel words taught by both accurate and inaccurate speakers [6,7,14,15]. On the other hand, ME is a linguistic constraint whereby children assume a one-to-one correspondence between labels and objects, leading them to map novel labels onto unfamiliar objects rather than familiar objects whose names they already know [16–20]. Although ME is a robust and early-emerging strategy, children can suspend this assumption depending on the learning context, such as when learning multiple labels for the same object [13,21–23]. Because speaker accuracy and ME represent distinct sources of information—one derived from social evaluation and the other from lexical knowledge— examining how children integrate and utilize multiple cues during word learning is important for understanding the mechanisms that support successful vocabulary acquisition.

In natural learning environments, children must regularly disambiguate words and their referents using information provided by speakers alongside their own lexical knowledge. These cues may provide convergent information (e.g., a caregiver labels an unfamiliar object with a novel word) or conflicting information (e.g., a caregiver applies a novel label to an object whose name is already known). Such situations are not uncommon because children often encounter multiple potential referents, learn more than one label for the same object, or hear labels produced by speakers who are uncertain [22–24]. Consequently, children must weigh information from speakers against their existing lexical expectations when learning new words. Although previous studies have examined speaker accuracy and ME together during word learning, these studies have primarily focused on referent selection [25–27]. Immediate referent selection tasks provide insight into how children initially map words to referents, but it does not necessarily reveal whether these mappings are maintained beyond the initial learning episode. However, studies of word learning more broadly have examined children’s retention of novel label–object mappings after a longer delay of 5 or 10 minutes [28–30]. As a result, it remains unclear whether speaker accuracy and ME specifically influence children’s retention of novel label–object mappings following a short delay after the learning episode. The present study therefore examined the combined effects of speaker accuracy and ME on children’s short-term retention of novel label–object mappings.

### Role of speaker accuracy in word learning

Research shows that children use a variety of cues to assess the trustworthiness of an adult’s testimony [31–33]. One such cue is a speaker’s prior accuracy in labeling objects, wherein a speaker is considered accurate if they have labeled objects correctly in the past versus inaccurate if they have labeled objects incorrectly [9,12,34]. By the age of four years, children tend to display a preference for acquiring new information from speakers who have demonstrated accuracy in the past, as opposed to those who have been inaccurate [7,9,12,34,35]. Some studies have shown that when different speakers introduce a novel label for a novel object, children tend to select names provided by accurate speakers over inaccurate speakers during referent selection tasks [7,11,34,36]. For instance, Koenig and colleagues (2004) familiarized children with two speakers: one who consistently named familiar objects correctly (accurate speaker) and one who labeled the same objects incorrectly (inaccurate speaker). Afterward, both speakers introduced a novel object, each providing a different label (e.g., one said “*toma*” and the other “*mido*”), and after each exposure, children were asked, “*Can you tell me what this is called, a mido or a toma*?” Children consistently chose the label from the accurate speaker. Beyond referent selection, some studies have also examined whether speaker accuracy influences children’s delayed retention of novel label-object associations [6,7,15]. For example, in Koenig and Woodward (2010), children were shown two novel objects, one of which was labeled by either the accurate or inaccurate speaker (e.g., “*Look! That’s a blicket*”). Following a 2-minute delay, children were asked to identify the referent of the novel label. Children were less likely to select the intended referent when the label had been introduced by an inaccurate speaker than when it had been introduced by an accurate speaker. These findings suggest that children show a preference for information provided by accurate speakers during both referent selection and retention of newly learned label–object associations.

Yet, other studies have revealed a different pattern of results, showing that children can learn from both types of speakers but that they may do so in different ways [6,15,27,37]. For example, in a study by Gangopadhyay and Kaushanskaya (2022), 4- to 5-year-old children were introduced to four novel labels (two taught by the accurate speaker and two by the inaccurate speaker), each paired with a different novel object. Children’s short-term retention of these label-object mappings was tested using a visual world eye-tracking paradigm across multiple test trials. While children’s fixations to the target object were quicker for labels taught by the accurate speaker than for those taught by the inaccurate speaker, children’s short-term retention of novel label–object mappings did not differ between labels taught by the accurate and inaccurate speakers. Similarly, in a study conducted by Sabbagh and Shafman (2009), 4- to 5-year-old children were taught novel labels by either a knowledgeable or an ignorant speaker and were subsequently asked comprehension questions. When the question referenced the labeling episode (e.g., *“Which one did I say is the [modi/dawnoo]?”*), children correctly identified the object labeled by both the knowledgeable and ignorant speakers. However, when the question assessed semantic knowledge (e.g., “*Which one of these things is called [modi/dawnoo]?”*), children tended to select the object labeled by the knowledgeable speaker. These findings suggest that children do not entirely disregard information from previously inaccurate sources but may differentially integrate them into their learning.

Notably, investigations into speaker accuracy and word learning have revealed mixed findings, with some studies showing a preference for accurate speakers during referent selection and retention, and others showing that children can learn from both accurate and inaccurate speakers, with differences emerging in the speed of looking toward labeled objects [6,34]. Most of this work has focused on contexts involving novel, unfamiliar objects. Consequently, less is known about how children weigh speaker testimony against their existing lexical knowledge when these sources of information provide conflicting cues. Understanding how children resolve such conflicts is particularly important given evidence that children rely on assumptions about how words map onto objects, such as the principle of ME, during word learning.

### Role of mutual exclusivity in word learning

A powerful linguistic constraint that helps children differentiate between familiar and unfamiliar word-referent mappings is the principle of ME [17,18,20,38–40]. The ME principle posits that children assume each object has a single label. Thus, when encountering a novel word, children tend to link it to an unfamiliar object rather than assigning it as a second label to a known object. In disambiguation tasks, where children are shown a familiar object (e.g., a cup) and a novel object (e.g., a palette), children typically associate a new label (e.g., “*pilson*”) with the unfamiliar object [20].

In a seminal series of experiments, Markman and Wachtel (1988) demonstrated that 3-year-old English-speaking monolingual children tended to avoid assigning a novel label to an object whose name they already knew. In their task, children were presented with a familiar object and a novel object and were asked to *“show me the x,”* where *x* was a nonsense syllable, randomly assigned to the object. When the novel word was introduced in the presence of an unfamiliar object, children readily mapped the label to that unfamiliar object. In contrast, when the only object present was familiar, children tended to resist relabeling the object and instead interpret the novel word as referring to a part or attribute of the object. These findings provide clear evidence that children assume objects have a single label and actively search for alternative interpretations when this assumption is challenged. Some studies have shown that children can use ME to learn the meaning of words at earlier ages – Mather and Plunkett (2010) at 10 months and Halberda (2003) at 17 months. However, the ME bias is not applied consistently across all contexts [41,42].

Children can suspend the ME assumption when the learning environment provides clear pragmatic or contextual cues suggesting that multiple labels may appropriately apply to the same referent. For instance, children may relax their use of ME when a speaker explicitly signals a hierarchical or taxonomic relationship between labels (e.g., “*a poodle is a type of dog*”) or when social-pragmatic cues highlight that different names refer to different aspects of an object [41]. In addition, the communicative context itself can support flexible application of ME. For example, Diesendruck (2005) found that children were less likely to apply ME when a speaker was unlikely to know the conventional name of an object. Similarly, Diesendruck et al. (2010) demonstrated that children were less likely to select an unfamiliar object as the referent of a novel label when the speaker had previously demonstrated inaccurate language use. Together, these findings suggest that children’s use of ME is not rigid but is shaped by their evaluation of a speaker’s testimony.

### Combined impact of speaker accuracy and mutual exclusivity

Children draw on multiple sources of information during word learning, such as a speaker’s accuracy [10–12,14,15,26], and default assumptions about words’ meanings, such as ME [20,38–40]. Importantly, these cues do not operate in isolation, and children must often integrate and weigh multiple sources of information when interpreting novel labels [3,43,44]. It is plausible that the relative importance assigned to speaker accuracy and ME cues may vary depending on the learning context. When children trust a speaker, they are more likely to accept new labels from that individual, even if the label contradicts expectations based on ME [27]. For example, even though children already know the object as a cow, they may still accept a new label, particularly when the speaker has previously demonstrated referential intent [19,45]. Conversely, when the speaker has previously been inaccurate, children tend to fall back on their own heuristics, such as ME, and are less likely to accept conflicting information [25].

Critically, Birch et al. (2008) provided evidence that 3-year-old children do integrate speaker accuracy with ME when interpreting novel labels. Their study included a *Contrast Condition* designed specifically to test whether children treat a speaker’s testimony about word meanings as robust enough to support ME-based inferences. Children first learned a novel label (*“ferber”)* from an accurate speaker and from an inaccurate speaker in separate trials. In the Contrast Condition, two objects were presented, and each was labeled *“ferber”* once, one by the previously accurate speaker and one by the previously inaccurate speaker. When subsequently asked to find a different novel label (*“cheena”),* children were expected, based on ME, to map the new word to the object *not* previously labeled *“ferber”* by the accurate speaker. The key insight from this design is that children treated the accurate speaker’s label as sufficiently stable to support an ME-driven inference, whereas labels from an inaccurate speaker did not support such an inference. However, this paradigm used entirely novel objects for both the initial mapping and the contrast trials. Thus, the design depended on the novel word–novel object contrast, which differs from paradigms in which children must decide whether to map a second label onto a *familiar* object with an existing name [7,27], a form of ME violation that demands greater lexical flexibility.

Building on this body of work, Krogh-Jespersen and Echols (2012) provide valuable insights into how young children assess speaker labeling behaviors alongside ME during word learning. The authors tested 24-month-old children’s willingness to learn novel labels for either familiar or novel objects from an adult speaker using an immediate referent selection task. Each child was assigned 1 of 5 speakers labeling behavior conditions: accurate, inaccurate, knowledgeable, ignorant, or uninformative. Speaker labeling behavior was established during the first six familiarization trials, after which children participated in two novel label learning trials. In these trials, children heard a novel label applied to either a familiar object (second label condition) or a novel object (first label condition), separately. After both learning trials, children completed two test trials in which they were asked to identify a previously labeled object from an array of four items: the target, two familiar distractors, and one novel object. Results revealed that children in the first label condition readily applied a novel label to a novel object, regardless of the speaker’s accuracy. However, in the second label condition, their willingness to apply a new label to a familiar object varied by speaker type. Children were more likely to accept the second label from an accurate speaker but were less likely to do so when the speaker had previously been inaccurate or ignorant. These findings suggest that when a conflict arises between speaker testimony and children’s existing lexical knowledge, children selectively consider speaker labeling behavior. That is, children are more likely to accept a second label from an accurate speaker but rely on ME when the speaker is inaccurate. However, this study assessed children’s immediate referent selection rather than the retention of novel label-object mappings.

Word learning is a multi-stage process that extends beyond initial referent selection. Retention reflects more than immediate label-to-object mapping; it indexes the extent to which a newly learned word has been integrated into a child’s developing lexicon [46,47]. Retention can be assessed at multiple points following the learning phase, including across repeated test trials within the same session or after a delay. Examining performance beyond the initial referent selection task therefore provides insight into the stability of newly formed word–object associations [47,48]. This process is closely tied to children’s active weighing of cues, as the cues prioritized during learning can influence how newly acquired words are remembered over time [48]. Koenig and Woodward (2010) demonstrated that 24-month-old children learned novel labels for novel objects equally well when taught by accurate and inaccurate speakers during referent selection. However, when tested after a two-minute delay, children showed better retention of labels introduced by an accurate speaker than by an inaccurate speaker. These findings suggest that children form weaker label–object representations from inaccurate sources, directly influencing later word retention.

Taken together, existing studies suggest that children integrate speaker accuracy and ME during referent selection and that speaker accuracy can influence subsequent retention of newly learned labels. However, these two lines of research have largely developed independently. Prior studies examining the interaction between speaker accuracy and ME have primarily focused on immediate selection of a possible referent, whereas studies examining retention have typically manipulated speaker reliability without considering conflicts between speaker testimony and ME. As a result, it remains unclear how children retain newly learned word–object mappings when speaker accuracy and ME either align or conflict. Addressing this gap is important because real-world word learning often requires children to evaluate multiple, sometimes conflicting, sources of information when forming and retaining label–object associations over time. By around 4 years of age, children show increasing sensitivity to speakers’ epistemic cues [33,49,50], alongside rapid growth in cognitive [51,52] and linguistic abilities [53,54]. Together, these developing abilities support more flexible and strategic use of social and linguistic information. Children are now able to track speaker intentions, evaluate speaker credibility, and resolve conflicting referential cues [55], making this period particularly meaningful for understanding how children learn new words from different sources of adult input [56]. Understanding how children integrate speaker testimony with lexical expectations is therefore critical for explaining how word learning proceeds in situations where cues converge or conflict and, more broadly, for understanding the mechanisms underlying word learning.

### Current study

The current study examined the impact of speaker accuracy and ME cues on 4- to 7-year-old monolingual English-speaking children’s short-term retention of novel word–object mappings. Using a screen-based-response box paradigm, children in the current study were presented with yoked pairs of novel objects and familiar objects. Children were taught new labels for the objects by two different speakers, Accurate (always providing correct labels) and Inaccurate (always providing incorrect labels), under two conditions. In the Align-with-ME condition, speaker cues and ME pointed to the same referent, creating an unambiguous learning context (i.e., the Accurate speaker labeled the novel object with a novel name, and the Inaccurate speaker labeled the same novel object with a different novel name). In the Conflict-with-ME condition, speaker cues and ME provided conflicting information (i.e., the Accurate speaker labeled the familiar object with a novel label, while the Inaccurate speaker labeled the same familiar object with a different novel label), allowing us to test how children prioritize cues.

We tested children’s retention of novel label-object mappings introduced during the Teaching phase in two testing blocks. In the first block (novel object testing), children were presented with the two novel objects previously taught side by side on the screen and were asked to select the object that matched the novel word they heard. In the second block (familiar object testing), children were presented with the two familiar objects that had appeared during the Teaching phase, side by side and were asked to select the object that matched the novel word they heard.

In the Align-with-ME condition, where both cues supported mapping novel labels to novel objects, we expected children to demonstrate successful retention of novel word–novel object pairings. If children preferentially learn from accurate speakers, retention should be higher for labels introduced by the Accurate speaker than by the Inaccurate speaker. Alternatively, if children retain labels from both speakers, retention should not differ by speaker type. Because neither ME nor speaker cues supported assigning a novel label to a familiar object in this condition, we did not expect evidence of learning in the familiar-object testing block.

In the Conflict-with-ME condition, speaker testimony and ME provided competing information. If children prioritize ME over conflicting testimony, they should retain mappings consistent with ME (i.e., novel word–novel object associations). If children prioritize adult testimony, they should retain novel word–familiar object pairings. Moreover, if speaker accuracy modulates this prioritization, retention of familiar-object pairings should be stronger for labels introduced by the Accurate speaker than by the Inaccurate speaker. If children are indifferent to speaker accuracy, retention should not differ across speaker types.

## Method

### Participants

The final sample included 24 children aged 4–7 years (*M*_*age*_ = 5.9 years, *SD*_*age*_ = .96). To characterize the effect sizes that this sample size could reliably detect, a post hoc sensitivity analysis was conducted using G*Power 3.1 [57]. Assuming α = .05 and power = .80, a sample of 24 participants provides sensitivity to detect medium-sized effects (Cohen’s *f* ≈ .25) in repeated-measures comparisons. We also conducted a sensitivity analysis for the Speaker Accuracy x Condition (2x2) interaction using a paired-differences approach (i.e., comparing the difference between Accurate and Inaccurate speakers across Align-with-ME and Conflict-with-ME conditions). Assuming α = .05 and power = .80, a sample of 24 participants provides sensitivity to detect an interaction effect of approximately *dz* = 0.60. These calculations offer benchmarks for the detectable size of main and interaction effects in simple within-subject comparisons. The target sample size of 24 is also consistent with prior research examining children’s learning from accurate versus inaccurate speakers [9,12,58] and ME reasoning [18,59,60], which has included approximately 16–32 participants per age group. Given the within-subject design, in which all children completed both experimental conditions, each participant contributed data across conditions, increasing sensitivity to condition-level effects relative to between-group designs.

This study was approved by the Indiana University Institutional Review Board (IRB Protocol #10027) and was conducted as part of a larger ongoing project. The recruitment process involved reaching out to families who had previously participated in laboratory studies and had expressed interest in future involvement. Participant recruitment for this ^st^udy took place between 1^st^ October 2023 and 30^th^ November 2024. Prior to participation, written informed consent was obtained from the parents of all child participants, and children provided verbal assent. Consent and assent were obtained by trained research assistants who administered the study. All children participated in two sessions, each lasting approximately one hour. Sessions were scheduled with a minimum interval of seven days between them, and the maximum time between sessions did not exceed 21 days. Separating the experimental conditions across two visits helped minimize confusion between learning conditions and speaker accuracy status, while also reducing participant fatigue associated with completing experimental measures and standardized assessments within a single visit. All participants successfully passed a hearing screening at 20 dB across frequencies of 1000, 2000, and 4000 Hz. Additional exclusion criteria for all participants were hearing loss, psychological or behavioral disorders, neurological impairments, other developmental disabilities, and performance below 1.5 standard deviations from the mean on the nonverbal IQ test (see Table 1). Participants were further excluded if they met any two of the subsequent criteria: a standardized vocabulary score below 85, a diagnosis of language impairment, or parent-reported concerns regarding their child’s language development or skills. Following these inclusion and exclusion criteria, two children were excluded from the final analysis due to a history of language impairment.

**Table 1:** Participant Characteristics.

	*M (SD)*
**N**	24 (14 Female)
**Age (years)**	5.95 (0.96)
^**a**^ **Non-verbal IQ (std. scores)**	112.29 (12.05)
^**b**^ **Vocabulary (std. scores)**	114.83 (7.84)
^**c**^ **SES (years)**	17.68 (2.46)

^a^Matrices Subtest, Kaufman Brief Intelligence Test-Second Edition.

^b^Receptive One Word Picture Vocabulary Test – Fourth Edition.

^c^SES indexed by years of maternal education.

### Experimental design

The word-learning task consisted of three phases: Familiarization, Teaching, and Testing. In the Familiarization phase, children learned about two speakers—an Accurate speaker, who correctly labeled six familiar objects (e.g., always providing the correct label, such as saying *“house”* when shown a house), and an Inaccurate speaker, who consistently provided incorrect labels for those same familiar objects (e.g., always providing the incorrect label such as saying *“dog”* when shown a house). In the Teaching phase, both speakers labeled objects under two learning conditions: Align-with-ME, where a novel object received a novel label (consistent with ME expectations), and Conflict-with-ME, where a familiar object received a novel label (violating ME expectations). The Testing phase assessed children’s short-term retention of the novel label-object association using two forced-choice blocks presented in fixed order, a Novel Object Test Block, followed by a Familiar Object Test Block, but trials within each testing block were randomly presented.

#### Stimuli.

The Familiarization phase included a total of six familiar objects (doll, shoe, house, fork, sock and ice-cream) to be labeled by both the Accurate Speaker and Inaccurate Speaker. For the Teaching phase, eight novel labels with consonant–vowel–consonant structures that followed English phonotactic rules were generated. Novel labels were split into two lists ensuring that the lists matched on developmental sound classes of the consonants [61] and English biphone probability (frequency of two-sound combinations within a language obtained from the CLEARPOND database) [62]. Images of four novel objects were chosen from the NOUN (Novel Object and Unusual Name) database [63], ensuring equal salience and complexity across lists. Additionally, four familiar objects (ball, butterfly, cake and shirt), different from those presented in the Familiarization phase, were selected to be paired with each novel object in the ME paradigm. Each novel object was yoked with one familiar object to form novel–familiar object pairs. To ensure that learning effects could not be attributed to a specific label–object pairing, the associations between novel labels and novel objects for the two lists were counterbalanced across conditions for all participants. Furthermore, each label was assigned equally across conditions such that it was produced half the time by the Accurate Speaker and half the time by the Inaccurate Speaker. Refer to Fig 1 for a full list of the label–object pairings.

**Fig 1:**
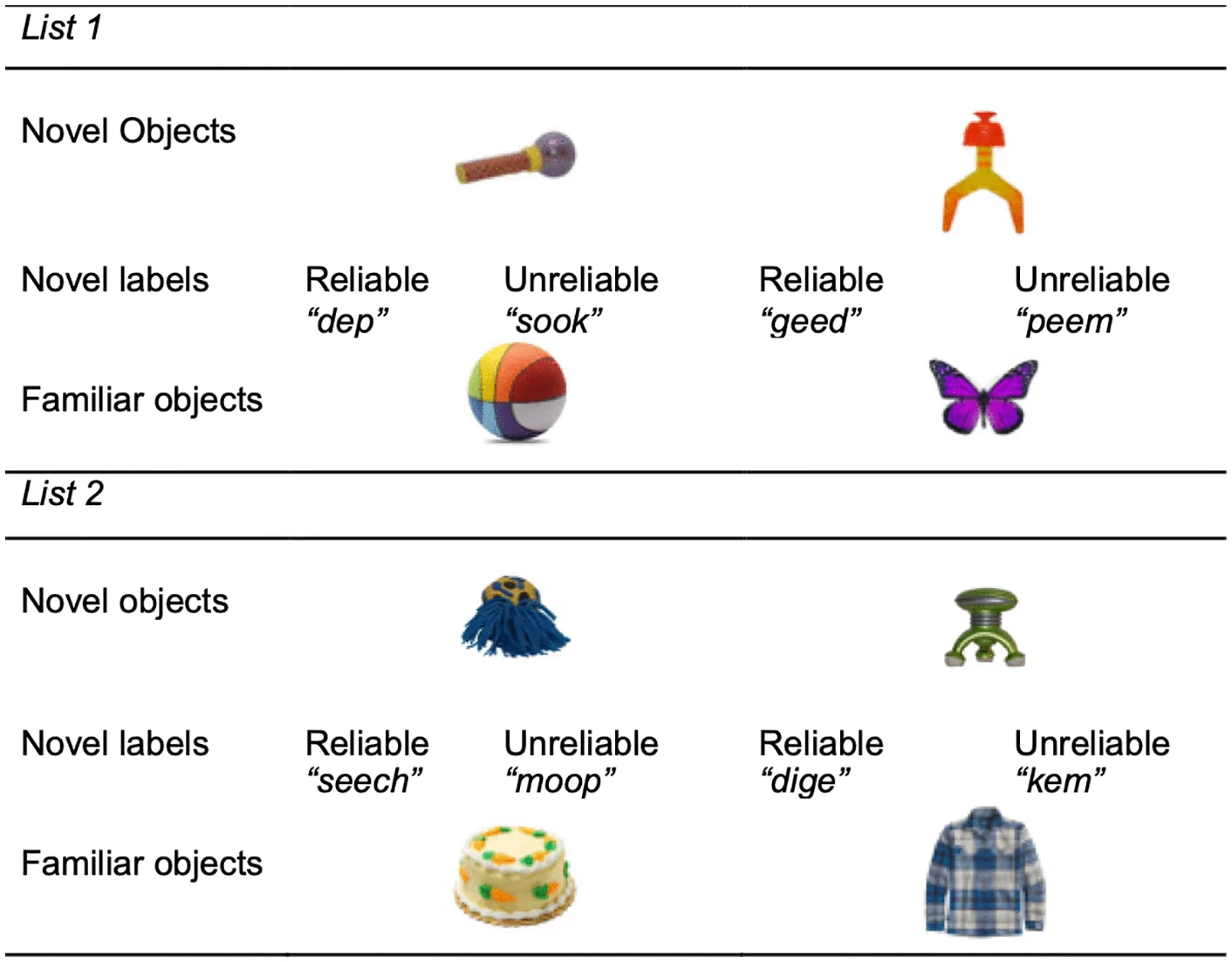
List of object-label pairings. Object–label pairings were counterbalanced such that “*dep*” was paired with each type of novel object in List 1. The novel object–familiar object pairs were yoked.

#### Recordings.

For the Familiarization and Teaching phases, we recorded videos featuring two adult females from the Midwest (actor A or B). These speakers had distinct facial features and wore different colored shirts for easy identification. To maintain uniformity across recordings, we edited all video recordings to minimize background noise, and the audio levels were standardized to 70dB. Each video segment was then carefully spliced to maintain a duration of approximately 5 seconds. For the test trials in the Testing Phase, a third adult female from the Midwest recorded the novel words and carrier phrases. These recordings were also spliced and normalized to 70dB. All recordings, whether for Familiarization, Teaching, or Testing phases, utilized child-directed speech.

#### Procedure.

**S**ee Figs 2, 3 and 4 for a visual representation of the task. Children were positioned in front of a computer screen with a resolution of 1920 x 1200. The order of presentation for each condition (Align-with-ME or Conflict-with-ME) was counterbalanced across children; half of the participants received the Align-with-ME condition in Session 1, while the other half received the Conflict-with-ME condition in Session 1. Prior to each session, children underwent a practice trial to acquaint themselves with the response-box button press. This involved presenting two familiar object pictures (not featured in the Teaching phase) and instructing the children to press the button (either right or left) corresponding to the picture named by the experimenter.

**Fig 2:**
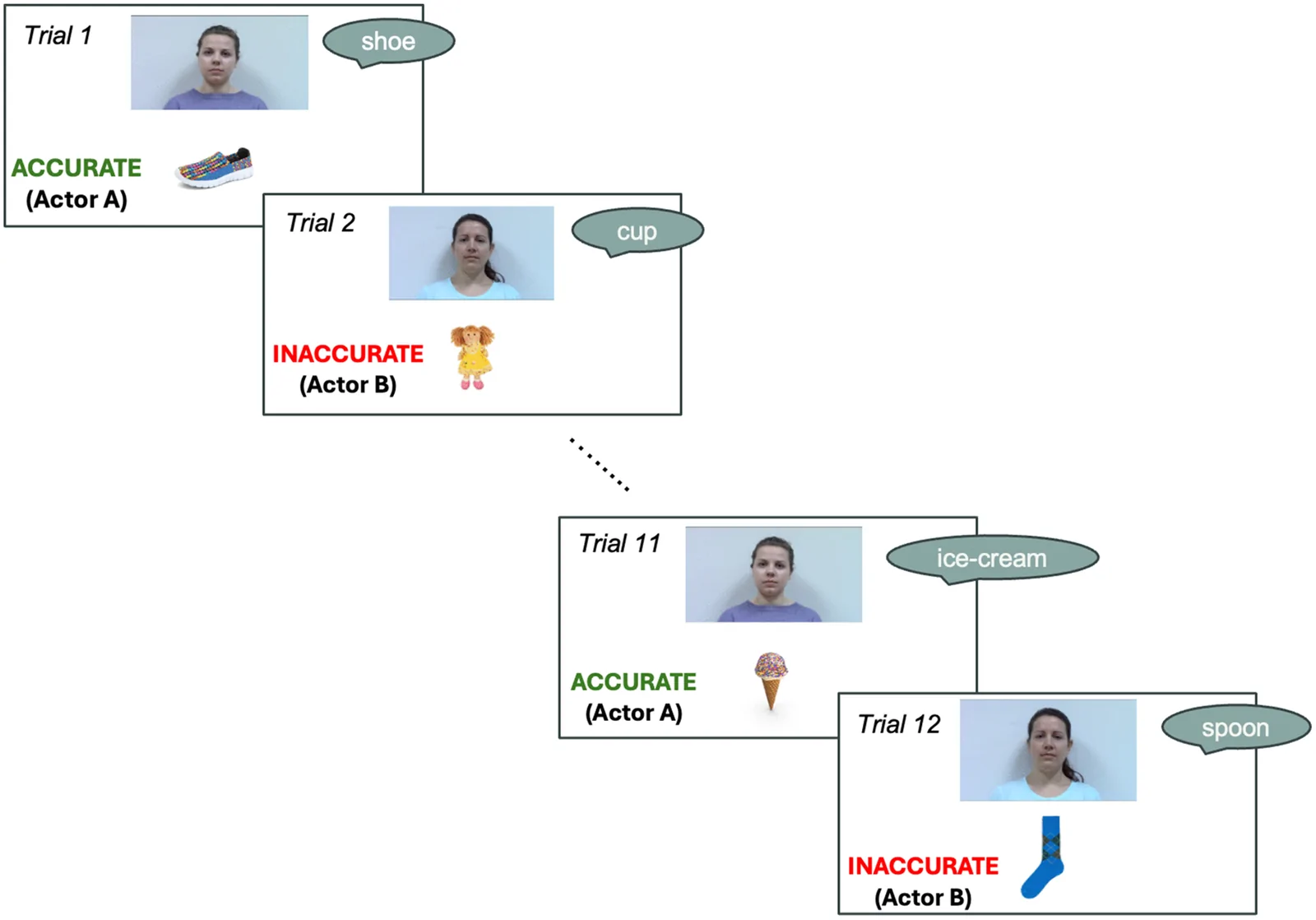
Familiarization Phase. To establish the accuracy of the speakers, one speaker explicitly and consistently provided correct labels for 6 known objects (e.g., shoe) and the other speaker consistently provided incorrect labels for the same 6 objects (e.g., labeling shoe as cup). Trials were randomized.

**Fig 3:**
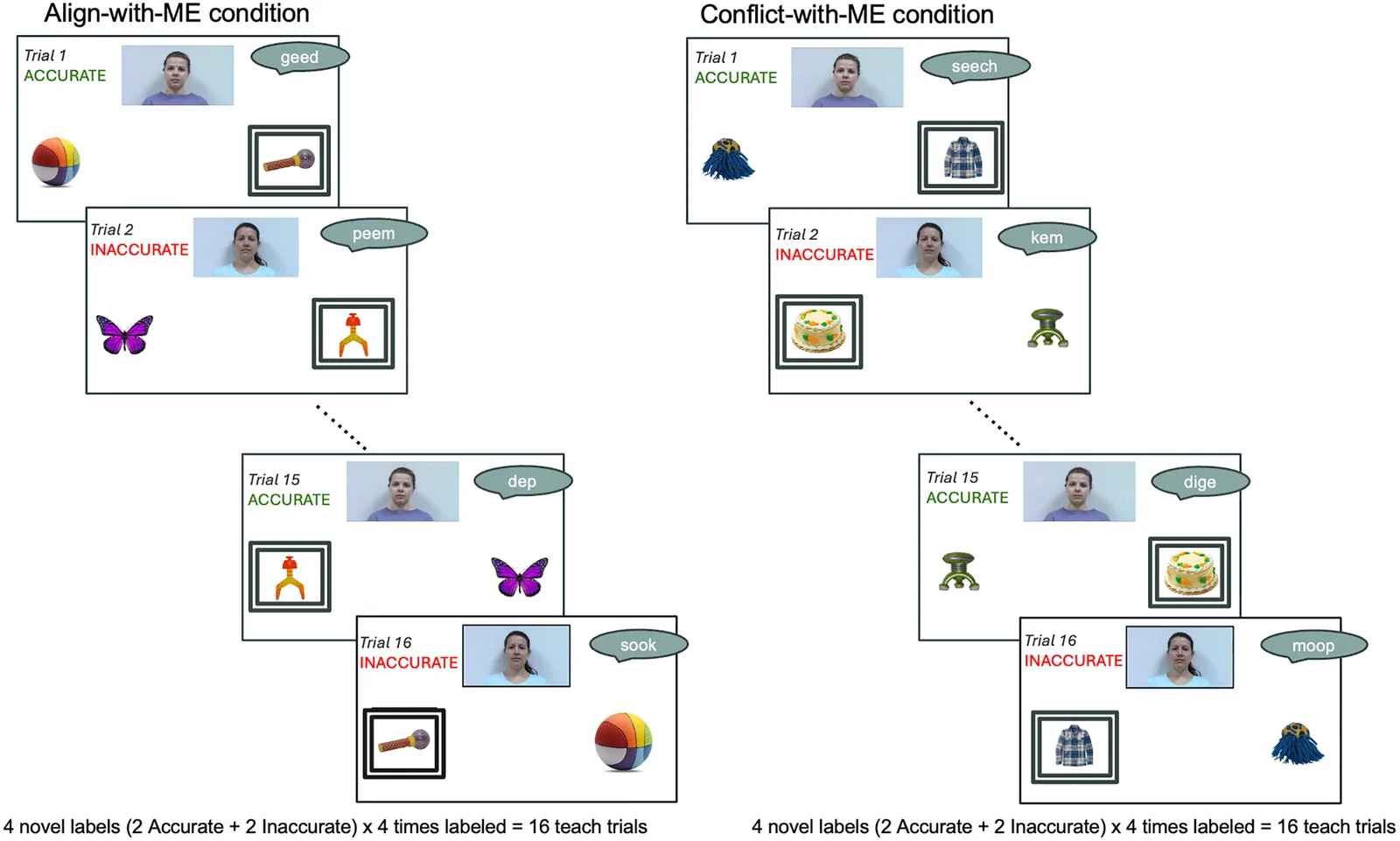
Example teaching trials for each Condition. Children were taught two new labels in each condition by each speaker type. Presentation of conditions was counterbalanced across children and label-object pairings were counterbalanced across conditions. Speaker presentation was randomized within a Condition. The double-lined black square indicates the object being labeled (target object) on a given trial. During the teaching videos, the target object moved in a bouncing motion while the speaker produced the label.

**Fig 4:**
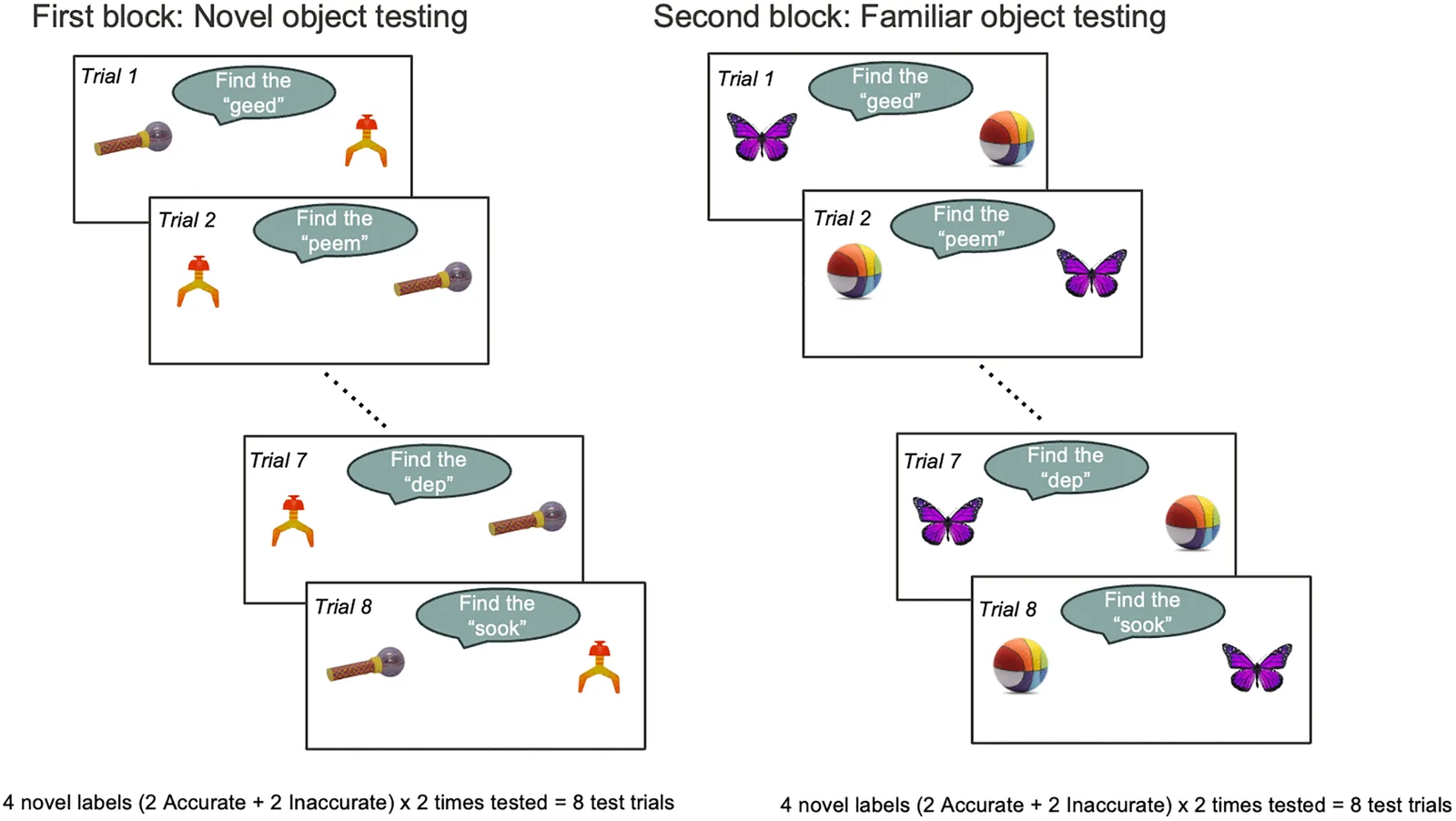
Example test trials for the Align with-ME condition from each testing block. The Conflict-with-ME condition had similar test trials, but with different novel and familiar object pairs.

*Familiarization.* The accuracy status of the two speakers was established during a Familiarization phase that occurred at the beginning of both Session 1 and Session 2. Children were instructed to *“Look and listen carefully!”* On each familiarization trial, a single familiar object appeared in the center bottom-half of the screen while one of the speakers appeared, looking straight in the center upper-half of the screen (for 2000 ms before the label onset) and produced a label for that object. The Accurate speaker consistently provided the correct label for all familiar objects (6 trials), whereas the Inaccurate speaker consistently produced incorrect labels for the same items (6 trials). A brief visual cue (a bouncing movement of the object was included to draw attention to the item being labeled; because only one object appeared on the screen, this bouncing movement did not serve as a referential disambiguation signal. The bouncing movement lasted 5000 ms, and the total duration of each teaching trial was 7 s. The order (first or second) in which the speakers appeared on the screen was randomized, and the assignment of speaker accuracy status (Accurate speaker or Inaccurate speaker) to actor (actor A or B) was counterbalanced across children. For each child, the assignment of speaker accuracy to actor identity was held constant across conditions (Align-with-ME and Conflict-with-ME), such that the same actor was always accurate and the other always inaccurate. The same two actors were used across both experimental conditions (Align-with-ME and Conflict-with-ME), but because the two sessions were separated by a minimum of seven days and the Familiarization phase was repeated for all children at the start of Session 2, interference or confusion about speaker accuracy status would be unlikely. Following the familiarization trials, children were shown images of both speakers and asked a brief set of questions about them. (1) Was one of them giving you all the right names? Which one? (2) Was one of them giving you all the wrong names? Which one? Children were not given any feedback during these questions and both pointing and verbal responses were permitted.

*Teaching.* After the Familiarization phase, children entered the Teaching phase, where all children were given the following instructions *– “Do you remember the two people you just saw? They will tell you the names of new pictures. All you have to do is listen very carefully.”* In the beginning of this phase, yoked pairs of novel and familiar objects appeared on either side (right or left) of the bottom-half of the screen (for 2000 ms before the label onset) that followed a ME paradigm. On each teaching trial, one speaker appeared on the center upper-half of the screen and produced the novel label (e.g., saying *“dep”*)*.* Labels were not embedded within carrier phrases, to ensure that children’s interpretations were driven by the label itself rather than by prosodic or syntactic cues. As the speaker labeled the object, at the same time, one of the two objects on the lower half of the screen (depending on Condition) made a bouncing movement to signal which object the speaker was naming. The bouncing movement lasted 5000 ms, and the total duration of each teaching trial was 7 s. This motion cue served solely to direct attention and was intentionally chosen instead of more socially informative cues (e.g., eye gaze), which could have introduced confounds with the speaker-accuracy manipulation. Using a non-social attentional cue ensured that any learning differences could be attributed to children’s weighting of speaker accuracy versus ME, rather than to their sensitivity to eye gaze or other ostensive signals. Importantly, children had already been exposed to the same motion cue during the Familiarization phase, where the bouncing object reliably corresponded to the labeled item. Thus, children understood that the bouncing object was the referent of the spoken label and that the cue was sufficiently salient for indicating the intended referent.

Children were taught labels under two learning conditions. In the Align-with-ME condition, speakers assigned novel labels to novel objects, providing children with an unambiguous context for using both ME and speaker accuracy cues. In the Conflict-with-ME condition, both speakers assigned novel labels to familiar objects, creating a violation of ME and requiring children to infer whether to rely on the speaker’s testimony or their own word knowledge. Each condition included two target objects, and for each target, children heard two different novel labels, one from each speaker, across eight randomized trials (four trials from the Accurate speaker and four from the Inaccurate speaker), resulting in sixteen teaching trials per condition.

In such a design, children sometimes heard a second novel label for an object they had just seen labeled. This choice reflects a central feature of the two-informant selective trust paradigm. A one-informant design (in which only one speaker labeled each object) would make it impossible to determine whether poorer performance under ME violation reflected (a) children’s distrust of an inaccurate speaker, or (b) a generalized reduction in performance because the input is unpredictable or counterintuitive. In contrast, presenting two conflicting labels from two distinguishable informants allowed selective trust to be assessed directly: children must decide which speaker’s information to retain. This two-informant structure also aligns with prior work demonstrating that this design offers a cleaner test of children’s cue integration and person-specific selection of reliability [64]. The order of speakers (Accurate or Inaccurate) and the location (left or right) of objects (novel or familiar) on the screen were randomized within each condition. The order of conditions (Align-with-ME vs. Conflict-with-ME) was counterbalanced across participants.

*Testing.* The Testing phase assessed children’s retention of the novel labels introduced during the Teaching phase. Testing consisted of two blocks presented in a fixed order. The Novel Object Test Block always occurred first, followed by the Familiar Object Test Block. Before each block, children received the following instructions: *“Now you will see two pictures and hear a word. Your job is to press the button for the picture that matches the word.”* No speaker was visible during testing. Instead, all test prompts were delivered by a third, unfamiliar speaker to prevent children from relying on visual or auditory recognition of the speakers during test trials.

On each test trial, two static images appeared side-by-side for 2000 ms before the auditory instruction. For the Novel Object Test Block, these images were the exact novel objects from Teaching; for the Familiar Object Test Block, the familiar objects were the exact same items previously paired with those novel objects during teaching. Thus, the test stimuli were identical tokens, not new category exemplars. Following the 2000 ms preview, children heard the prompt (*“Find the dep”*)*,* after which both images remained visible for up to 10 seconds. Children selected a response using the button box. Each of the four novel labels (Accurate or Inaccurate; Align-with-ME or Conflict-with-ME) was tested twice_—_once in the novel-object test block and once in the familiar-object test block. This resulted in eight trials per testing block. Trial order within each block was randomized for each participant and left-right placement of target objects was counterbalanced across trials and participants. Testing followed immediately after the Teaching phase within each session, separated only by the instructional slide (<30 seconds). Thus, retention of novel-labels was tested within-session without extended delays. An example trial for both testing blocks is provided in Fig 4.

### Measures

#### Parent questionnaires.

Parents completed a background questionnaire providing information on language, medical, and educational histories of their children. This questionnaire also gathered data on mothers’ years of education, serving as a proxy for children’s socioeconomic status (SES) [65].

#### Standardized assessments.

To assess English vocabulary knowledge, children were administered the Receptive One-Word Picture Vocabulary Test–Fourth Edition (ROWPVT-4) [66]. The test consists of a series of 4 pictures, each representing different objects, actions, or concepts. The examiner says a word, and the child is asked to select the picture that best represents that word. All children were also administered the Matrices subtest of the Kaufman Brief Intelligence Test (KBIT-2) [67], a test of nonverbal intelligence that consists of a series of visual patterns presented in a matrix format. For each item, the child is shown a matrix with a missing piece and asked to select the correct piece that completes the pattern from a set of multiple choices. These standardized assessments provided a descriptive profile of the sample, ensuring that children were within typical limits of developmental and language ability ranges.

#### Analyses.

Children’s retention was analyzed separately for the Novel Object Test Block and the Familiar Object Test Block. For each child, accuracy was converted into a proportion of correct responses for each of the four learning conditions (Accurate–Align-with-ME, Inaccurate–Align-with-ME, Accurate–Conflict-with-ME, Inaccurate–Conflict-with-ME). To determine whether children performed above chance (0.50), we used one-sample Wilcoxon Signed Rank tests. We selected Wilcoxon tests for two reasons: (1) children’s proportion-correct scores were bounded and often showed non-normal distributions, violating assumptions for parametric tests; and (2) visual inspection of the data confirmed skewed and clustered distributions, supporting the use of a distribution-free test. Because our hypothesis concerned whether performance exceeded chance, the Wilcoxon signed-rank tests were conducted as one-tailed tests against chance level (.50).

Following these analyses, we fit GLMM’s to examine the influence of speaker accuracy and learning condition at the trial level. Separate GLMMs (with a binomial error distribution) were constructed for novel-object and familiar-object trials because the two testing blocks assessed different possible label–object mappings: novel label–novel object mappings in the novel object testing block and novel label–familiar object mappings in the familiar-object testing block. Speaker accuracy (contrast coded as – 0.5 for Accurate and 0.5 for Inaccurate), learning condition (contrast coded as – 0.5 for Align and 0.5 for Conflict) and their interaction were entered as fixed effects and participant included as a random intercept. Potential item-level effects were initially evaluated by including test word as a random effect. However, the resulting model produced a singular fit, with negligible variability attributable to individual test words (*Variance* = 0.00, *SD* = 0.00). In addition, inclusion of the item random effect did not improve model fit relative to the participant random-intercept model (*AIC* = 521.20 vs. 519.20, respectively), and the pattern of fixed effects remained unchanged. Therefore, test word was not included as a random effect in the final analyses. Finally, adding by-participant slopes as random effects to the models resulted in singular fits and did not meaningfully alter the pattern of fixed effects. Therefore, the simpler random-intercept model was retained for the final analyses.

## Results

All 24 children correctly identified which speaker was accurate and which speaker was inaccurate, and thus, no children were excluded from the analysis.

### Chance performance analysis

In the Align-with-ME condition, where both speakers labeled the novel object with novel labels, children retained novel labels for novel objects, significantly above chance from both the Accurate speaker (*M* = 0.61, *SD* = 0.32, *p* = .037) and the Inaccurate speaker (*M* = 0.71, *SD* = 0.27, *p* = .001). In contrast, children did not demonstrate above-chance retention of novel labels for familiar objects, regardless of whether the labels were introduced by the Accurate speaker (*M* = 0.38, *SD* = 0.30, *p* = .966) or the Inaccurate speaker (*M* = 0.41, *SD* = 0.24, *p* = .967). The latter finding was expected because in this condition, familiar objects were not the targets of labeling and therefore, not intended to be associated with the novel labels. See Fig 5 for participants’ mean accuracy for novel object and familiar object testing in Align-with-ME condition.

**Fig 5:**
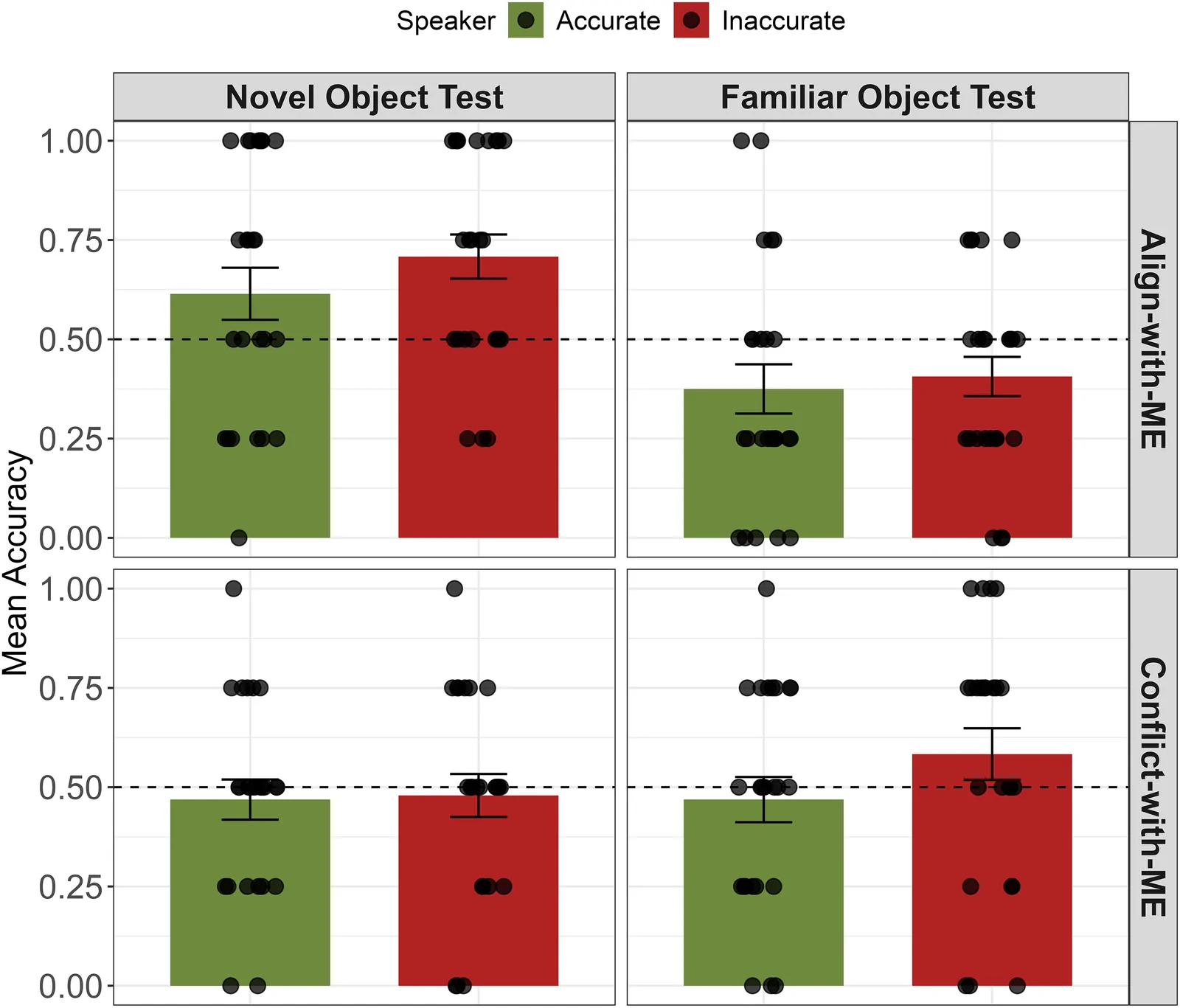
Task accuracy across learning conditions and testing blocks. Dashed horizontal line indicates chance. Error bars represent standard errors of the mean. In the Align-with-ME condition, children performed above chance for both speaker types in the Novel Object Testing (*p < .05). In the Conflict-with-ME condition, children did not perform above chance for either speaker type in either testing block.

In the Conflict-with-ME condition, where both speakers labeled the familiar object with novel labels, children did not demonstrate above-chance retention of novel labels for novel objects for either the Accurate Speaker (*M* = 0.47, *SD* = 0.25, *p* = .747) or the Inaccurate Speaker (*M* = 0.48, *SD* = 0.26, *p* = .688). Children also did not retain novel labels for familiar objects above-chance for the Accurate speaker (*M* = 0.47, *SD* = 0.28, *p* = .734) or the Inaccurate speaker (*M* = 0.58, *SD* = 0.32, *p* = .130). To address the possibility of below-chance performance, we conducted post-hoc two-tailed Wilcoxon signed-rank tests for all learning conditions. None of the conditions showed significant below-chance performance (all *p*s > .05), indicating no evidence that children formed consistent non-target associations. These results suggest that children did not prioritize ME nor the speaker’s testimony (accurate or inaccurate) when the cues conflicted. See Fig 5 for participants’ mean accuracy for novel object and familiar object testing in Conflict-with-ME condition.

### Novel object test trials

As the sample included children aged 4–7 years, we evaluated the model with Age (centered) as a covariate. Including age did not improve model fit (*AIC* increased from 519.20 to 520.25), and age was not a significant predictor (*b* = 0.12, *SE* = 0.12, *z* = 0.98, *p* = .326). Thus, we report results from the simpler model without Age. With the chosen contrast coding (−0.5, 0.5), the lower-order effect of learning condition represents the difference between conditions averaged equally across the two levels of speaker accuracy, and the lower-order effect of speaker accuracy represents the difference between speaker accuracy levels averaged equally across the two learning conditions.

GLMM analysis showed a significant effect of condition (*b* = −0.79, *SE* = 0.21, *z* = −3.71, *p* < .001), where children’s accuracy of retaining the novel labels was significantly higher in the Align-with-ME condition (*M* = 0.66, *SD* = 0.47) compared to the Conflict-with-ME condition (*M* = 0.47, *SD* = 0.50). This indicates that children were more successful at retaining novel labels when Speaker testimony aligned with ME expectations than when those expectations were violated. There was no significant main effect of speaker accuracy (*b* = 0.23, *SE* = 0.21, *z* = 1.10, *p* = .270), such that children did not show significantly different learning outcomes for labels introduced by the Accurate speaker (*M* = 0.54, *SD* = 0.50) compared to the Inaccurate speaker (*M* = 0.59, *SD* = 0.49). This suggests that, when averaged across learning conditions, children did not preferentially weigh speaker accuracy status (accurate or inaccurate) when retaining novel labels. Finally, there was no interaction between speaker accuracy and condition (*b* = −0.38, *SE* = 0.42, *z* = −0.90, *p* = .366), indicating that the effect of learning condition on label retention did not differ as a function of speaker accuracy. The model included a random intercept for participant, with an estimated variance of 0.05 (*SD* = 0.22). See Table 2 for full model results.

**Table 2:** Model results for novel object test trials.

	*b*	*SE*	*95% CI*	*z-value*	*p-value*
**Intercept**	0.29	0.12	[0.06, 0.52]	2.52	0.011
**Accuracy**	0.23	0.21	[−0.18, 0.65]	1.10	0.270
**Condition**	−0.79	0.21	[−1.21, −0.37]	−3.71	<.001
**Accuracy x Condition**	−0.38	0.42	[−1.21, 0.45]	−0.90	0.366

### Familiar object test trials

As with the novel-object analyses, adding Age (centered) as a covariate to the mixed-effects model did not improve model fit (*AIC* increased from 527.23 to 528.23), and Age was not a significant predictor (*b* = –0.13, *SE* = 0.14, *z* = −0.92, *p* = .360). We report here results from the simpler model without Age. As in the novel-object analyses, fixed-effect estimates of condition and accuracy are interpreted at the average level of the other predictor due to contrast coding.

GLMM analysis showed a significant effect of condition (*b* = 0.57, *SE* = 0.21, *z* = 2.70, *p* = .006), where children’s accuracy of retaining the novel labels was significantly better in the Conflict-with-ME condition (*M* = 0.53, *SD* = 0.50) compared to the Align-with-ME condition (*M* = 0.39, *SD* = 0.49). These findings are consistent with task expectations, as familiar objects were the intended referents of novel labels only in the Conflict-with-ME condition, whereas in the Align-with-ME condition, familiar objects were not labeled and therefore not expected to be associated with novel labels. This suggests that children’s responses reflected sensitivity to which objects were intended as referents within the learning context. There was no significant main effect of speaker accuracy (*b* = 0.31, *SE* = 0.21, *z* = 1.46, *p* = .145), such that children did not differ in their retention of novel labels introduced by the Accurate speaker (*M* = 0.42, *SD* = 0.50) compared to the Inaccurate speaker (*M* = 0.49, *SD* = 0.50). There was also no interaction between speaker accuracy and condition (*b* = 0.34, *SE* = 0.42, *z* = 0.81, *p* = .417), indicating that the effect of learning condition on familiar-object test performance did not vary as a function of speaker accuracy. The model included a random intercept for participant, with an estimated variance of 0.15 (*SD* = 0.39). See Table 3 for full model results.

**Table 3:** Model results for familiar object test trials.

	*b*	*SE*	*95% CI*	*z-value*	*p-value*
**Intercept**	−0.18	0.13	[−0.44, 0.08]	−1.33	0.184
**Accuracy**	0.31	0.21	[−0.11, 0.72]	1.46	0.145
**Condition**	0.57	0.21	[0.16, 0.99]	2.70	0.006
**Accuracy x Condition**	0.34	0.42	[−0.49, 1.17]	0.81	0.417

## Discussion

The present study investigated the combined effect of speaker accuracy and ME on novel word retention in monolingual English-speaking children. We found that all children successfully retained novel label-object mappings when speaker testimony aligned with ME expectations. Specifically, children showed above-chance retention for novel label–novel object pairings from both Accurate and Inaccurate speakers. This suggests that when speakers introduce novel labels in a manner consistent with children’s expectations about one-to-one word–object mappings, learning is supported regardless of speaker accuracy status [27]. However, when speaker testimony conflicted with ME, by applying novel labels to familiar objects, children did not retain novel labels above chance for either novel objects or familiar objects, and speaker accuracy had minimal impact on retention. This unexpected pattern of results indicates that conflict between speaker testimony and ME expectations constrained children’s ability to form stable word–object representations in this particular learning context.

### Novel object testing

In line with our predictions, when speaker testimony and ME aligned (i.e., when speakers labeled the novel objects with novel names), children successfully retained the novel labels for novel objects from the speakers. Moreover, children retained labels taught by both the accurate speaker and inaccurate speaker, suggesting that children are willing to learn new words for unfamiliar objects regardless of whether the speaker has been accurate at labeling in the past. In other words, despite children’s clear knowledge of which speaker was accurate and which was inaccurate, children retained novel labels taught by both speakers to the same extent. On the other hand, when speaker testimony conflicted with ME (i.e., when the speakers labeled the familiar objects with novel labels), children did not exceed chance levels of performance during novel object retention testing. These findings corroborate previous research on multiple cues [27,68,69], in that when cues converge on the same meaning, children’s novel word mappings are supported. Further, novel word learning appears to benefit from the convergence of multiple cues not only at the moment of mapping a label to a referent but also in supporting the retention of newly learned label-object associations [70].

Our findings converge with earlier work by Koenig and Woodward (2010), which demonstrated that even young children (24-month-olds) learn novel labels for novel objects equally well from both accurate and inaccurate speakers. In their design, the speaker provided novel label training across two blocks: (1) a first-label condition (a novel object labeled with a novel label) and (2) a familiar-object condition (e.g., *“mido”* for shoe; dubbed a “second label” because children already know shoe). Paired with the target was a distractor object, both during training and testing. Even though there were differences in our paradigm where speakers provided novel labels on separate trials, with a novel object and a familiar object presented side by side on the screen, older children were still able to learn from both types of speakers. Notably, children encountered both an accurate and an inaccurate speaker across the experiment, and judgments about speaker accuracy were built gradually across multiple exposures rather than through immediate comparison. Prior research also suggests that in such single-informant paradigms (as in [7]), children are more likely to accept information from both speakers, particularly when no alternative label is presented [71]. In addition, because the novel labels were consistently paired with novel objects and did not violate children’s existing lexical knowledge, children may have relied on multiple converging cues during learning. Specifically, repeated label–object pairings across learning trials may have supported the formation of label-object associations using cross situational learning alongside ME expectations and speaker testimony, consistent with a multiple-cue account of word learning.

In the Conflict-with-ME condition, where speakers labeled the familiar objects with novel names, children did not successfully retain novel labels for novel objects from either accurate or inaccurate speakers. The fact that children did not perform above chance for novel-object testing in the Conflict-with-ME condition suggests that ME was not sufficiently influential to guide learning. In other words, if children had strictly adhered to ME, they would have mapped the novel label onto the novel object, ignoring the conflicting information from the speakers. It is conceivable that children may have suspended the use of ME altogether. Supporting this interpretation, our GLMM analysis revealed a significant effect of Condition, where children performed more accurately in the Align-with-ME than in the Conflict-with-ME condition. One possible explanation for the poorer performance observed in the Conflict-with-ME condition is that introducing novel labels for objects that the children were highly familiar with might have created confusion in this learning context, resulting in uncertainty regarding the intended referent. Consequently, children may have failed to form stable word–object mappings, leading to chance-level performance during testing.

### Familiar object testing

To examine how children integrate multiple cues during word learning, particularly when cues conflict, the present study included testing trials assessing children’s ability to retain novel labels for familiar objects. In the Conflict-with-ME condition, the design allowed us to test whether children trusted the speaker’s testimony more than their usual assumption that new words refer to unfamiliar objects. The present study found no evidence that children retained novel labels for familiar objects from either the accurate or inaccurate speaker,

inconsistent with prior findings (e.g.[27],). Moreover, although model predictions revealed a significant main effect of condition, suggesting greater accuracy for familiar object testing in the Conflict-with-ME condition than the Align-with-ME condition, children did not perform above chance in either of the two conditions. Any accuracy in the Align-with-ME condition likely reflects random responses because familiar objects were not labeled at all. Therefore, with familiar object testing, we found that children did not prioritize speaker accuracy over ME [72].

Several factors may explain these findings. Our results diverge from Krogh-Jespersen and Echols (2012), who reported that children accepted a second label for a familiar object from an accurate speaker but not from an inaccurate speaker. A key difference between the studies is the age range of participants: Krogh-Jespersen and Echols tested 24-month-olds, whereas our study included children aged 4–7 years. While sensitivity to speaker accuracy may emerge by 3–4 years of age [7,27], younger children may be more vulnerable to speaker accuracy—perhaps stricter about whom they end up learning from—during second-labeling scenarios. In contrast, older children, with more developed metacognitive and linguistic skills, may integrate speaker accuracy with other available cues during word learning rather than relying predominantly on a single cue. However, the current sample did not provide sufficient power to test an interaction between age and speaker accuracy. Future work with larger samples could examine whether the influence of speaker accuracy on word learning varies with age.

Another reason for this pattern of results is that the bouncing object available during learning may not have been interpreted as a strong indicator of speaker intent. Although speaker accuracy status was established during the Familiarization phase, the bouncing object during labeling may not have carried enough communicative weight to override children’s existing lexical knowledge. This difficulty may have been even greater in the familiar-object trials. In these trials, ME would be a particularly strong constraint because children already had existing labels for the familiar objects. Unlike in the novel-object trials, children encountering familiar objects had to overcome both a strong prior expectation about the object’s known name and the speaker’s testimony. Additionally, because the Testing phase involved only auditory instructions from a recording of a third, unfamiliar speaker, children did not have access to the original speakers who had provided the labels. This lack of visual continuity may have limited children’s ability to make immediate judgments based on prior speaker accuracy. We further note that the degree of perceived similarity between the test speaker and the teaching speakers was not formally assessed. Therefore, we cannot completely rule out the possibility that some children associated the test speaker’s voice with one of the speakers from the teaching phase.

Lastly, it is important to note that testing consisted of two blocks presented in a fixed order. The Novel Object Test Block always occurred first, followed by the Familiar Object Test Block. The Novel Object Test Block was presented first because it provided the more intuitive testing situation for children. In this block, children were asked to identify novel objects associated with newly learned labels, whereas the Familiar Object Test Block required children to consider whether a novel label could refer to an object for which they already knew a conventional label. We anticipated that beginning with the novel-object trials would reduce potential confusion and help establish the task demands before children encountered the more challenging familiar-object trials. Nevertheless, this fixed presentation order may have influenced children’s performance. Future studies should consider alternative testing formats that allow novel and familiar referents to be assessed within a single testing phase and use implicit measures of word learning (e.g., eye-tracking), which can provide insight into the real-time dynamics of children’s recognition of newly learned label–object associations. Although some children may have experienced mild fatigue or reduced engagement by the second block, the two blocks were brief (approximately 12–15 seconds each), and testing followed immediately after teaching trials. Thus, while fatigue cannot be ruled out entirely, the observed differences across testing blocks are more consistent with the additional inferential demands required when ME is violated rather than with systematic declines in attention.

The present findings may also have implications for understanding word learning in children with different language experiences. Children growing up in multilingual environments may develop different expectations about label-object mappings because they regularly encounter multiple labels for the same object across languages. As a result, multilingual children may be more flexible in suspending ME assumptions or may weigh speaker cues differently during word learning [73,74]. Examining the influence of language environment on the interplay between speaker reliability and ME could help establish whether these mechanisms operate similarly across different linguistic contexts or whether there are important variations to consider.

Altogether, while this study makes valuable contributions to our understanding of how speaker accuracy and ME jointly affect children’s novel word retention, several important questions remain for future exploration. First, in the present study, speaker accuracy and ME may not have been the only sources of information available to children during learning. Consistent with a multiple-cue account of word learning, children may have drawn on additional cues when forming and retrieving novel word–object associations. For example, the repeated presentation of word–object pairings across teaching trials may have allowed children to track co-occurring statistics between labels and referents. Although the present study was designed to examine how speaker accuracy and ME jointly influence children’s learning, the design does not allow us to isolate the contribution of cross-situational statistical learning or other contextual cues that may have supported performance. Importantly, this possibility does not diminish the role of speaker accuracy and ME in the present findings. Rather, it highlights that children’s word learning likely reflects the integration of multiple sources of information. Future research should directly manipulate and compare these cues to better understand how children weigh speaker testimony, lexical expectations, and statistical regularities when learning new words.

Second, children’s age may have influenced their performance in this study compared to prior work [7,27]. While previous research primarily tested toddlers using simpler word learning tasks, our study involved a more complex design targeting 4- to 7-year-olds. Although this age group typically exhibits more developed cognitive and linguistic skills, the combination of conflicting cues, the low salience of the bouncing object and the absence of speakers in the Testing phase of our task may have posed unique learning challenges.

Third, our study focused solely on novel and familiar objects. This design did not account for a broader range of stimuli or contexts that may influence word retention (for instance, introducing multiple unfamiliar objects during testing, varying levels of object familiarity, or embedding word learning within richer, socially dynamic interactions). Future research would benefit from investigating how different categories of objects (e.g., animate vs. inanimate) or different scenarios (e.g., interactive vs. observational) impact children’s word learning. Such studies would provide a more comprehensive understanding of how various speaker cues interact with ME across diverse contexts.

Finally, we considered whether performance patterns across the task might reflect engagement issues, particularly given the overall low performance observed across conditions and children’s failure to successfully learn novel labels in the conflict condition. We initially speculated that task repetition and a lack of variation may have led to reduced engagement. However, we found no behavioral evidence of boredom as no children dropped out of the study, and all of them completed both sessions. Additionally, the shift in speaker identity between the Teaching and Testing phases may have contributed to confusion. Because the original speakers were no longer present during testing, children needed to recall speaker accuracy information without ongoing social or visual support. This disruption may have made it harder for children to connect what they learned during Familiarization and Teaching phases to the decisions required during the Testing phase. Rather than introducing more familiar trials to increase engagement, future studies might consider including more interactive or socially engaging task elements (e.g., live interactions or animated characters) to sustain attention while maintaining experimental control.

It is also important to note that in the present study, testing occurred within the same session and after a relatively short interval following the teaching phase. Therefore, our findings should be interpreted as reflecting children’s retention of newly formed word–object associations beyond the initial mapping event rather than long-term retention assessed after an extended delay. Future work should examine whether the effects observed here persist when children are tested after longer delays. Together, these considerations highlight how even older children may struggle with tasks involving complex cue integration, especially when memory loads and speaker discontinuity are introduced.

## Conclusion

The findings of this study contribute to our understanding of how children integrate speaker accuracy and ME cues for novel word retention. Most interestingly, when ME and speaker testimony provided conflicting information, children did not prioritize ME or speaker testimony. Therefore, neither cue was sufficiently influential to guide consistent learning, underscoring how conflicting signals can sometimes hinder word learning entirely [70]. This outcome raises questions about how children interpret cues provided by the speaker and whether they are strong enough to supersede children’s default lexical assumptions. Future research should examine how the perceived strength and communicative value of different sources of information—such as speaker testimony and lexical constraints—shape children’s ability to retain novel words. Further, while prior studies have demonstrated successful cue integration in younger children under simpler conditions, our findings highlight how task demands and methodological design can shape learning outcomes in older children. Future work should also consider using more ecologically valid designs (e.g., live speaker interactions or dynamic visual cues) and nuanced methods (e.g., eye tracking) to better understand the mechanisms underlying cue prioritization and children’s decision-making in real time.
