# Supplementary material for: The role of speaker accuracy and mutual exclusivity in children’s novel word learning
Source: PLoS One. 2026 Oct 29;21(10):e0361483. doi: 10.1371/journal.pone.0361483 (PMC13718952; doi:10.1371/journal.pone.0361483)
Supplement: Supplementary file 1 [file pone.0361483.s004.pdf]

I was involved in both the first and second rounds of review and was very pleased to see how substantially the manuscript has improved in response to the thoughtful revisions made by the authors and the suggestions raised during the first round of review. In particular, I found the revised Introduction much clearer and well structured. The addition of a conceptual introduction and the more explicit formulation of the main focus and research gap considerably strengthen the motivation for the study. The revised Method section has also benefited substantially from the new structure and figures, which make the procedure much easier to follow.
The majority of the points I raised in the first round have been addressed satisfactorily. I have a smaller number of remaining comments, most of which are minor and concern points that either emerged while reading the revised manuscript or that concern a clarification of comments I raised in the first round. I outline these points below.

1. Abstract:

- The revised abstract is much clearer and, in my view, now provides a good overview of the study. One point that remained somewhat unclear to me concerns the final conclusion ("that the relative weighting of these cues may vary depending on the learning context") – could the authors clarify how this conclusion follows from the reported findings?

2. Introduction:

- The revised manuscript now makes the focus on children's (short-term) retention across different learning contexts much clearer. I noticed, however, a slight asymmetry in the literature reviewed: The section on speaker accuracy discusses previous findings concerning both referent selection and retention, whereas the section on ME appears to focus primarily on children's referent selection. The authors may decide whether they prefer to retain this focus, or whether they want to mention previous empirical findings on retention of word-object mappings established through ME in this section.

3. Method

- p. 22: I think the reading flow might be slightly improved if the model itself were introduced and explained first, followed by the explanation of why other random effects were not included.

4. Results

- One remaining point concerns the explanation of the GLMM coefficients at the beginning of the Results section (based on a comment I raised in the first round). I appreciate that the authors have added a clarification in response to my previous comment. However, I think the current wording could still be made somewhat clearer.
  - The authors state that "Because speaker accuracy and learning condition were contrast coded (-0.5, 0.5), fixed-effects estimates are interpreted at the average level of the other predictor. Thus, the reported effects reflect average differences across levels of the other factor rather than effects at a specific reference category". I have two concerns with the phrasing of these sentences:
    - This interpretation does not concern all "fixed-effects estimates"/ "the reported effects", but specifically the lower-order terms for condition and speaker accuracy, as these predictors are also included in the interaction.
    - Further, referring to the "average level" of a categorical factor might be somewhat confusing, as there is no actual intermediate level between, e.g., an accurate and an inaccurate speaker (instead, the effect of, e.g., condition is the average of the effect of condition for accurate speakers and the effect of condition for inaccurate speakers). Therefore, it would be clearer to explain directly that, with the chosen contrast coding, the reported effect of condition represents the difference between conditions averaged equally across the two levels of speaker accuracy – and vice versa for the effect of speaker accuracy. (This distinction is particularly relevant if the numbers of observations across the two levels are not equal, because the model coefficient then does not correspond to simply collapsing the observed data across speaker accuracy.)

- I noticed that some of the numerical values reported in the main text of the Results section are not identical to the ones reported in Table 2.
- In response to one of my comments from the first round, the authors state that “We have now combined Figures 5 and 6 as suggested”. In the revised manuscript, however, the two figures still appear separately. I was wondering whether an outdated version of the figure has been uploaded, or alternatively whether the response letter was not updated to reflect the final revision?
- Finally, in my previous review I suggested specifying in the captions of Figures 5 and 6 what the error bars represent (e.g., SE, SD, 95% confidence intervals etc.). This information still appears to be missing. Although this is a minor point, I think it would be useful to add so that the figures can be interpreted unambiguously.

5. Discussion

- The authors have clearly made an effort to incorporate the additional discussion points raised during the first round of review. I found these additions very valuable. In a few places, however, the transitions between the original discussion and newly added material felt somewhat abrupt.
    - E.g., p. 30: “some children associated the test speaker’s voice with one of the speakers from the teaching phase” – the transition to this point could perhaps be made a little more fluent, and it might be helpful to briefly spell out the implication of this possibility.
    - E.g., p. 33/34: the part beginning with “It is also important to note …” might work better as a separate paragraph. At present, both the transition into this point and the transition from it to the concluding sentence felt somewhat abrupt.

6. Reference list

- Reference 71 appears to contain a short paragraph that may have been intended as a footnote?
