# Supplementary material for: The role of speaker accuracy and mutual exclusivity in children’s novel word learning
Source: PLoS One. 2026 Oct 29;21(10):e0361483. doi: 10.1371/journal.pone.0361483 (PMC13718952; doi:10.1371/journal.pone.0361483)
Supplement: Supplementary file 4 [file pone.0361483.s005.docx]

We thank the three anonymous reviewers for their helpful suggestions on the improvement of our manuscript. Below is a point-by-point response (in italics) to each comment, with “**Response:**” clearly indicated for easy reference. We have also tracked our revisions in the manuscript, highlighted in yellow.

**Reviewer #1**

This study is examining the extent to which 4-to-7 year-old monolinguals weigh mutual exclusivity (ME) versus speaker testimony when learning novel words. Learners were exposed to four conditions: accurate speaker in align-with-ME, inaccurate speaker in align-with-ME, accurate speaker in conflict-with-ME, inaccurate speaker in conflict-with-ME. Findings suggest that children learned better when ME wasn’t violated, regardless of speaker accuracy history.

This is a novel and important contribution to the literature and fits the scope of the journal. The introduction and the design are especially robust. I have a few comments on the analyses that should only require a minor revision.

***Response:*** *We are grateful to Reviewer 1 for their positive review of the manuscript, and for highlighting the importance of our study. We are especially thankful for the Reviewer’s suggestions to describe our analysis plan in more detail.*

**Major comments**

1. Why not add item as a random effect as well since the analyses are said to be done at the trial level on line 481?

***Response:*** *We thank Reviewer 1 for this question. In our initial analysis, we indeed added test word as an item-level random effect. The resulting model produced a singular fit, with negligible variability attributable to individual test words (Variance = 0.00, SD = 0.00). In addition, inclusion of the item random effect did not improve model fit relative to the participant random-intercept model (AIC = 521.20 vs. 519.20, respectively), and the fixed-effect estimates remained unchanged. Therefore, item as a random effect was removed from the final model.*

*We have updated the Analyses section (p.22-23, lines 534-549) to describe the model comparison procedure and rationale for retaining the simpler model. The revised R analysis script has also been included in the OSF repository (*[*https://osf.io/5x2ze/files/osfstorage*](https://osf.io/5x2ze/files/osfstorage)*).*

2. Did you remove by-participant slopes because of convergence and singularity issues? If so, could you add a sentence to describe this?

***Response:*** *We thank the Reviewer for this question. The Reviewer is correct. We evaluated models with random slopes for the main effects of speaker accuracy and learning condition, as well as their interaction. However, these resulted in singular fits and negligible differences in model fit relative to the simpler participant random-intercept model (e.g., AIC = 518.86 vs. 519.20).*

*The Analyses section (p.23, lines 547-549) has been updated to clarify the model comparison procedure, final random-effects structure, and model specification.*

3. Why is the estimate negative for the main effect of condition in the Novel object test trials GLMM? If Align with ME is coded as 0.5 and that learning was higher in that condition than in the Conflict with ME, shouldn’t the estimate be positive (and vice-versa for the next GLMM)?

***Response:*** *We are grateful to Reviewer 1 for identifying this discrepancy. We incorrectly described the contrast coding scheme in the manuscript, although the analyses themselves were conducted using the correct coding specification, that of Align as -0.5 and Conflict as 0.5.*

*We have now revised the contrast coding description in the Analyses section (p.22-23, lines 544-546) to accurately reflect the coding used in the analyses and reported results.*

4. What was the rationale for having two sessions? Was it because this project is part of a larger one or was there a specific rationale? Either way, it would be helpful to specify this.

***Response:*** *We thank the reviewer for this helpful suggestion.There were methodological and practical reasons for conducting the study across two sessions. Because the study employed a within-subjects design (i.e., all children completed both Align-with-ME and Conflict-with-ME learning conditions), separating the conditions across two sessions would minimize confusion between learning conditions and speaker accuracy status. Moreover, the study included the word learning task as well as standardized assessments; dividing participation across two visits reduced burden and fatigue for young children.*

*We have included a rationale for conducting the study over two sessions in the Procedure section (p.14, lines 323-326).*

5. In the description of the testing phase, I am confused with these sentences: “Each novel label was tested twice, once in each of the two blocks. This resulted in 8 trials per testing block.” Aren’t there only 2 blocks, novel object block and a familiar object block? Therefore, “tested once in each block” would mean 4 trials across both sessions, not 8? Perhaps, the sentence should be corrected to “Each of the 4 novel labels (accurate or inaccurate, in align-with ME or conflict-with ME) was tested twice, per blocks. This resulted in 8 trials per testing block.”

***Response:*** *We thank the reviewer for catching this error, and we agree that the original wording was unclear. We have revised the description of the testing phase to clarify that each of the four novel labels was tested twice, per block, resulting in eight trials per testing block (p.21, lines 492-495).*

**Minor comments**

6. The second sentence of the abstract is repeated twice.

***Response:*** *We have rectified this error.*

7. Small detail: the recruitment dates could be written with the month in letters – I read the first one as 10th Jan. but based on the second one, it’s probably 1st Oct.

***Response:*** *We have now done so (p.13, line 318).*

8. I was not able to access the OSF files; an error message popped up, at least for the .xlsx files (I did not try the R files).

***Response:*** *We thank the reviewer for bringing this issue to our attention. We have now updated the repository permissions, verified access to all uploaded materials, and re-uploaded the relevant files. In addition, we have added the revised R analysis script and session counterbalancing scheme to the repository for transparency. All files should now be accessible through the OSF link provided in the manuscript.*

**Reviewer #2**

My major concerns surround the structure of the Introduction, the discussion of the theoretical framework, the design of the study, the one-sample tests, and the interpretation of results.

***Response:*** *We thank Reviewer 2 for their thoughtful and constructive feedback on our manuscript. We have carefully revised the manuscript to strengthen the conceptual framing of the study, clarify terminology, provide additional methodological details and justifications, and refine our interpretation of the findings within a broader multiple-cue framework. We have also improved the overall organization and flow of the manuscript.*

**Major Comments**

1. I suggest streamlining the first two paragraphs and using only one paragraph to provide an overview of the background and state the aim of the study.

***Response:*** *We thank the reviewer for this helpful suggestion. We have now revised the original first two paragraphs to provide a more concise overview of the theoretical background and study rationale.*

2. Details of previous work should be worked into later paragraphs. In places where there are subheadings, links to previous paragraphs should be made explicit to allow for a better flow. Also, some careful thinking is needed to discuss related information closer together to avoid unnecessary repetition. One way would be to look at how citations are repeated throughout the Introduction and bring the discussion around the same citations closer together.

***Response:*** *We appreciate Reviewer 2 for this valuable suggestion. We have reorganized the Introduction to improve its overall flow. We have provided clearer transitions between subsections to explicitly connect the discussion of speaker accuracy, mutual exclusivity, and their combined role in word learning.*

3. In terms of theoretical framework, the authors outlined the multiple-cue account of word learning. However, often, the authors create a false dilemma between speaker accuracy and mutual exclusivity (e.g., lines 127-128). According to the multiple-cue account, children would draw on any informative cues when learning the meanings of new words and the reliance of different cues may vary across contexts (see Chan & Monaghan, 2025). The study’s focus on two particular cues does not rule out children’s use of other cues. The constant omission of other cues throughout the manuscript is rather problematic and has serious implications for the interpretation of results. In particular, since the pairing of objects in the teaching and test phases was different, correct identification of objects at test could be due to cross-situational statistical learning – this is especially relevant given that the children identified the correct novel objects in the Align-with-ME regardless of speaker accuracy, suggesting that they actually accepted lexical overlap to some extent (based on my understanding that two names were introduced to each object, one by the accurate speaker and one by the inaccurate speaker).

***Response:*** *We thank the Reviewer for this insightful comment. We recognize that we may have framed speaker accuracy and mutual exclusivity as competing cues in some sections of the manuscript, which is inconsistent with a multiple-cue account of word learning. In response, we have revised the Introduction and Discussion to more explicitly acknowledge that children draw on multiple sources of information during word learning and that the relative weighting of these cues may vary across learning contexts. We have also removed language that could be interpreted as children relied on speaker accuracy or mutual exclusivity in isolation and instead emphasize how these cues may operate alongside other sources of information.*

*Furthermore, we have expanded on this as a limitation in the Discussion section to indicate that children’s performance may have been influenced by other cues not directly examined in the present study, including cross-situational statistical learning. We now discuss the possibility that children integrated multiple cues when learning and retrieving novel word–object associations and note that future research should further investigate the relative contribution of these cues to word learning outcomes (p.32, lines763-777).*

4. A major concern about the design is that it is unclear why the task is referred to as a retention/recall task. First, given the timings of the task, the time lag between teaching and test could be as short as 30 s. This is considerably shorter than the conventional delay of at least 5 minutes used in the word-learning literature to ensure retrieval from long-term memory (Horst & Samuelson, 2008). A clear explanation and justification for not using a longer delay is needed for claims to be made about retention/recall. Further, even if the short delay could be seen as involving long-term memory, the lack of an immediate referent selection test makes it impossible to see if the results were truly about poor retention or if they were about poor learning in the first place. A more careful consideration of whether the results speak to learning or retention is needed.

***Response:*** *We thank the Reviewer for this comment. We agree that the distinction between immediate referent selection, learning, and retention should be articulated more clearly (a similar concern raised by Reviewer 3). Our intention was not to make claims about long-term retention or retrieval from long-term memory, but rather to examine children's ability to retain newly learned word–object associations following completion of the teaching phase. We use the term retention to distinguish performance during the testing phase from immediate referent selection (i.e., disambiguation). Prior work has emphasized that successful referent selection does not necessarily result in stable word learning and that word learning is a dynamic, multi-stage process that extends beyond the initial mapping of a label to its referent (e.g., Horst & Samuelson, 2008; McMurray et al., 2012). Consistent with this distinction, several studies have assessed children's ability to retrieve newly learned word–object mappings across multiple trials following completion of the learning phase and have referred to this process as immediate retention, including when testing occurred within the same experimental session (e.g., Bion et al., 2013; Escobar et al., 2023; Pomper & Saffran, 2019; Wojcik, 2019). Importantly, unlike traditional referent-selection paradigms in which children identify a referent immediately after hearing a novel label, all teaching trials in the present study were completed before testing began. Children were therefore required to retrieve previously learned label–object mappings across multiple test trials following the learning episode.*

*We have now revised the manuscript to clarify the distinction between referent selection and immediate retention of label-object mappings (Introduction, p.4, lines 79-87 and p.10, lines 225-231), and have refined our terminology throughout.*

*We also acknowledge that, because the study did not include an immediate referent selection measure, we cannot definitively determine whether chance-level performance reflected failures of initial learning, retrieval, or a combination of both. We have, therefore, tempered our interpretation of the findings in the Discussion. Finally, we have revised the Introduction and Discussion to more clearly articulate the study's contribution as examining children's immediate retention of word–object associations rather than long-term retention following an extended delay.*

5. One other question about the task is how distinguishable was the voice of the third speaker in the test phase from those of the first and second speakers in the teaching phase? If the voices could not be clearly distinguished, the children may have attributed the voice in the test phase to one of the previous speakers, which may have implications for their performance.

***Response:*** *We thank Reviewer 2 for this question. The test speaker was a different individual from the two speakers who participated in the teaching phase (now clarified in the Method section, (p.20, lines 478-480). However, because the degree of perceived similarity between the test speaker and the teaching speakers audio recordings were not formally assessed, we cannot completely rule out the possibility that some children may have associated the test speaker's voice with one of the speakers from the teaching phase. We have, therefore, noted this as a limitation of the study and include that future studies could introduce a third speaker visually during testing to make sure that children realize that this is a completely different speaker (see p.30-31, lines 727-730).*

6. The results would be more appropriately interpreted as cues that do not violate established lexical knowledge allowed cross-situational statistics to override mutual exclusivity, regardless of speaker accuracy. And the reason for not observing the same in the Conflict-with-ME condition would be that the introduction of novel labels to objects that the children were overly familiar with confused them, leading to them not learning anything. Hence, the chance-level performance. In short, the authors need to take other cues into account when discussing literature and interpreting the results.

***Response:*** *We appreciate Reviewer 2’s suggestion about more carefully interpreting our findings, especially because children's performance may have been influenced by multiple sources of information available during learning. We have now revised the Discussion (p.28, lines 668-674) to acknowledge that successful performance in the Align-with-ME condition may reflect the combined influence of multiple cues, including mutual exclusivity expectations, repeated label–object pairings, and speaker testimony, rather than reliance on any single cue in isolation.*

*We also agree with the Reviewer that chance-level performance in the Conflict-with-ME condition may reflect the increased difficulty associated with learning novel labels for highly familiar objects. We have therefore expanded the Discussion to include this alternative interpretation and consider how competing lexical representations may have contributed to poorer performance in this condition. These revisions have been incorporated into the Discussion section (p.28-29, lines 677-679 and lines 684-689).*

7. Regarding the one-sample tests, it appears to me that the reported tests were one-tailed, as the graph for the familiar object trials in the Align-with-ME condition seems to suggest that performance was significantly below chance, but the statistics suggest otherwise. Was there a rationale for conducting one-tailed tests? This has serious implications for the interpretation of results, because if the children performed significantly below chance, it means that they were showing preference for a different object than expected. This would demonstrate associations of ‘not the target’ has been formed, which would be evidence for statistical learning taking place. If this was the case, I wondered if the manipulation of speaker accuracy was relevant at all. It could be that the other cues (i.e., cross-situational statistics and their lexical knowledge) were more salient and the children relied on them solely and disregarded speaker accuracy completely. This would mean that the study did not actually test the effect of speaker accuracy and any claims about speaker accuracy would be unwarranted. In this case, the authors did not really test what they set out to investigate.

***Response:*** *We thank the Reviewer for this thoughtful comment. The one-sample Wilcoxon signed-rank tests were conducted as one-tailed tests because our hypotheses were directional and specifically predicted performance above chance rather than below chance. We now make this rationale explicit in the original manuscript in the Analyses section (p.22, lines 530-532).*

*However, the Reviewer’s point is well taken, and we conducted additional analysis to examine whether performance in all learning conditions differed significantly from chance using two-tailed tests. None of the conditions, specifically including the familiar-object testing, yielded performance values significantly below chance (all ps > .05). Therefore, we do not interpret the results as evidence of a systematic preference for the other object or as direct evidence that children formed "not-the-target" associations. We now add a footnote (see Reference list 71 ) on conducting a post-hoc two-tailed analysis to confirm chance performance across learning conditions.*

*Footnote - To address the possibility of below-chance performance, we conducted post-hoc two-tailed Wilcoxon signed-rank tests for all learning conditions. None of the conditions, specifically including familiar object testing differed significantly from chance performance (all ps > .05), indicating no evidence that children formed consistent non-target associations.*

Minor Comments: Below are responses to substantial minor comments. All other suggestions and corrections have been incorporated into the manuscript. We have also tracked our revisions in the manuscript, highlighted in yellow.

8. It would be good to start with some background information.

***Response:*** *We have now revised this section.*

9. (lines 28-33) The description of the design of the study could be made clearer and more succinct.

***Response:*** *We have now revised this section.*

10. (lines 70-74) This sentence is utterly difficult to follow. Please check that the information here is accurate and that it follows logically from the previous sentence.

***Response:*** *We have now removed these lines and revised the paragraph.*

11. (lines 101-104) The writing here could be clearer. I suggest explicitly comparing against accurate speakers here and replacing ‘correct’ with ‘intended’. Also, it should be made clear in the description of the study that the children were not tested on immediate referent selection, but a slightly delayed identification/recall.

***Response:*** *We have made the suggested changes (see p.5, lines 108-110).*

12. (line 113) Please explain/elaborate on what is meant by ‘equally well’.

***Response:*** *We have revised this (see p.5, lines 117-122).*

13. (lines 405-424) Please provide detailed information on the timings of event. How long were the objects shown before the word was spoken? How long did each trial last?

***Response:*** *We have now added this information under Procedure, specific to each phase in the experiment.*

14. (line 481) Please provide clear justification for running separate GLMMs for the novel and familiar blocks.

***Response:*** *We have now added this information (see p.22-23, lines 540-544).*

**Reviewer #3**

After reading the manuscript carefully, my main concerns primarily relate to a) the conceptual and theoretical integration of the research question within the existing literature, b) the clarity and strength of the study’s motivation and rationale, c) the structure of the methodological description, and d) the implementation and interpretation of their statistical analysis. I will explain each of these points, along with additional minor comments, in detail below.

***Response:*** *We thank Reviewer 3 for their thoughtful and constructive feedback on our manuscript. We have carefully revised the manuscript to provide a stronger theoretical foundation, improve the structure of the Method section, provide additional methodological details and justifications, and refine our interpretation of the findings.*

1. I experienced the writing style of the abstract as somehow segmented and lacking a (short) conceptual introduction of the topic

***Response:*** *We have now added a brief conceptual introduction to the Abstract and revised it for better flow.*

2. “The combined effect of speaker accuracy and mutual exclusivity (ME) was assessed” --> Could the authors define the effect of interest (effect on …?)

***Response:*** *We have now added “The present study examined the combined effect of speaker accuracy and mutual exclusivity (ME) on retention of label-object associations in 4-7-year-old English-speaking monolingual children.” to the abstract to indicate what was assessed.*

3. The 2nd and 3rd sentence are redundant

***Response:*** *We have rectified this error.*

4. The introduction moves rather quickly to empirical findings. It would benefit from a stronger conceptual and theoretical framing at the outset, including more background and a strong argumentation why the two focal cues (ME and speaker accuracy), and especially their combined effect, are theoretically important.

***Response:*** *We thank Reviewer 3 for this helpful suggestion. We agree that the Introduction would benefit from a stronger conceptual and theoretical framing. We have now reorganized the Introduction to begin with a broader multiple-cue account of word learning and strengthened the rationale for examining speaker accuracy and mutual exclusivity together. Specifically, we now emphasize that speaker accuracy and mutual exclusivity represent distinct sources of information that children must coordinate during real-world word learning. We have also streamlined the presentation of previous empirical work and more clearly articulated the theoretical importance of understanding how these cues jointly influence children's learning.*

5. Further, the introduction would benefit from a clearer argumentation for the study’s rationale, by highlighting the novel contribution. In particular, if the main aspect of interest is the focus on children’s retention, as opposed to their immediate referent selection, this argumentation needs to be clearer, including the theoretical importance of assessing retention performance, as well as concrete differences to prior studies (e.g., p. 9 “However, this study assessed children’s immediate referent selection rather than the retention” – The authors may want to specify in how far this immediate referent selection differs from the immediate retention in the present study?)
6. p. 9: If the distinction between disambiguation and retention is of particular interest for the current study, and since retention is mentioned much earlier, introducing this contrast earlier in the manuscript (and clarifying its theoretical implications and relevance) would improve the structure and clarity of the study motivation.

***Response:*** *We thank Reviewer 3 for the valuable suggestions in numbered points 5 and 6, and we respond to these together here.*

*Indeed, the distinction between immediate referent selection (i.e., disambiguation) and retention of word–object mappings requires a clearer articulation in the Introduction. We note that a similar concern was raised by Reviewer 2, and we have collectively addressed this issue throughout the revised manuscript. Specifically, we have introduced this distinction earlier in the paper (Introduction, p.4, lines 79-87) and have expanded our rationale for focusing on retention as an outcome of word learning.*

*We now clarify that immediate referent selection reflects children's ability to identify a potential referent at the moment of learning, whereas retention provides information about the stability of newly formed word–object associations beyond the initial mapping event. We also discuss the theoretical importance of examining retention in the current study and highlight how this contribution differs from prior studies that primarily assessed immediate referent selection. These revisions have been incorporated into the Introduction (p.10, 224-230), where we more explicitly motivate the study's novel contribution and clarify the relevance of assessing retention performance.*

7. p. 5 “While children’s looking patterns differed as a function of speaker accuracy” --> How did they differ?

***Response:*** *We have now added this information (see p.5, lines 107-112).*

8. p. 7 (reference to Kalashnikova et al., 2016): From the current description, it is not entirely clear what the study is intended to illustrate in this context. If the goal of this paragraph is to show that children sometimes suspend ME, there is a substantial body of literature that could support this point more directly: e.g., work by Diesendruck shows that children avoid the ME conclusion if the speaker uses proper nouns instead of conventional words (Diesendruck, 2005), if the speaker doesn’t know the conventional word because they don’t speak the language in question (Diesendruck, 2005) or because they proved to be an inaccurate language user (Diesendruck et al., 2010). This line of work seems highly relevant to the current study and could be incorporated here or elsewhere in the manuscript.

***Response:*** *We greatly appreciate Reviewer 3 for the suggestion to include studies by Diesendruck and colleagues to highlight more directly children’s variable adherence to mutual exclusivity. Accordingly, we have revised our discussion of mutual exclusivity and replaced the Kalashnikova et al. (2016) example with findings from Diesendruck (2005) and Diesendruck et al. (2010), which demonstrate that children can suspend mutual exclusivity depending on a speaker's knowledge, conventional language use, and communicative context. These revisions strengthen the theoretical connection between mutual exclusivity and speaker characteristics and are now reflected on p.7 lines 165–170 in the Introduction.*

9. p. 10: The section on the “combined impact of speaker accuracy and mutual exclusivity” would benefit from a clearer articulation of what remains unsolved by the current literature to motivate the study.

***Response:*** *We thank Reviewer 3 for the suggestion to provide further clarification on the rationale for examining the combined effects of speaker accuracy and mutual exclusivity. In the revised manuscript, we have strengthened the “Combined impact of speaker accuracy and mutual exclusivity” section in the Introduction by more explicitly identifying the gap in the existing literature. Specifically, we now highlight that prior studies have primarily examined the interaction between these cues using immediate referent selection paradigms, leaving open the question of how speaker accuracy and mutual exclusivity jointly influence the retention of newly learned word–object associations. We have also strengthened the transition from review of prior work to motivation for the present study, thereby clarifying the study's novel contribution (p.11, lines 256-259).*

10. p. 11: “In the first block (novel object testing), children were presented with two novel objects …”: Here and in the following sentence, could the authors specify that these refer to the previously presented novel objects and words?

***Response:*** *We thank the Reviewer for this question. We have revised this section to clarify that the novel objects, familiar objects, and novel labels presented during testing were the same items that had been introduced during the Teaching phase (see p.12, lines 274-280).*

11. The formatting and hierarchy of section headers seem inconsistent and might need revision (e.g., “Stimuli” as sub-header of “Measures”; “Recordings” at the same level as “Stimuli”; “Design” as sub-header of “Recordings” & at the same level as the components of the study; no “Procedure”, etc.) Further, the ordering of sections seems rather unintuitive and would benefit from a clearer structure (e.g., presenting the Design and Procedure before the Measures would make the manuscript easier to follow; the “Experimental task” is currently listed under Measures (p.14) – it would be more intuitive to describe this information directly before the procedure (i.e., the single components) are described).

***Response:*** *We have reorganized the Method section to improve the structure and flow of the experimental task and other measures used in the study.*

12. In general, from the description of the procedure and design details, it was challenging to reconstruct the design fully, including the number of trials per condition, session etc., as relevant information is distributed across sections. Providing a clearer overview of the design (both in-text and potentially visually) would greatly improve readability.

***Response:*** *We have reorganized the Method section to improve the structure and flow of the experimental task and other measures used in the study. In addition, we have revised Figures 2, 3, and 4 to use consistent objects and labels across figures and have added information about the number of trials included in each phase of the task within each session to improve clarity and readability.*

13. Relatedly, Figures 3 & 4: It would be helpful if the figures illustrated a complete sequence of example trials using consistent stimuli (same objects/ words across figures). This would make it easier to see how different trial types are constituted and relate to each other.

***Response:*** *We have now added this information to Figures 3 and 4 (pages 18,20 and 22).*

14. Figure 3: Could the authors specify whether the information about the number of labels refers to both sessions or only one (and possibly add a similar information to the test trials/ Figure 4)?

***Response:*** *We have now added this information.*

15. p. 17/18 “Using a non-social attentional cue ensured that any learning differences could be attributed to children’s weighting of speaker accuracy versus ME, rather than to their sensitivity to eye gaze or ostensive signals” --> Could the authors clarify this argument? It is not immediately obvious to me why this decision would produce less differences between tasks than, e.g., the consistent use of speaker pointing/ looking at one object.

***Response:*** *We appreciate the opportunity to clarify our rationale for using a non-social attentional cue during the Teaching phase. Our goal was to minimize children's reliance on highly salient social-referential cues, such as eye gaze or pointing, which could independently support label–object mappings. By using a simple motion cue to indicate the intended referent, we aimed to more directly examine children's use of speaker accuracy and mutual exclusivity during word learning.*

16. p.19: “This resulted in 8 trials per testing block”: Per block or per condition/ session? A more explicit overview of the procedure earlier in the section would probably help clarify this as well.

***Response:*** *We have revised the description of the testing phase accordingly. The sentence now clarifies that each of the four novel labels was tested twice, once in each of the two testing blocks, resulting in eight trials per testing block (p.20, lines 492-495).*

17. What was the reasoning behind deciding against a delay between the learning and test phase (although retention strictly refers to retrieving information from the long-term memory)?

***Response****: We appreciate the opportunity to clarify this point. In the revised manuscript (Introduction, p.4, lines 79-87 and p.10, lines 225-231) we now more clearly distinguish between immediate referent selection and retention of word–object mappings and explicitly discuss word learning as a multi-stage process. We also clarify that our intention was not to make claims about long-term retention or retrieval from long-term memory, but rather to examine children's ability to retain newly learned word–object associations across multiple trials following completion of the learning phase. In addition, we now acknowledge in the Discussion (p.33-34, lines 805-811) that the relatively short interval between teaching and testing differs from delayed retention paradigms commonly used to assess long-term memory and discuss this as a limitation of the current study.*

18. Why did the novel object test block always precede the familiar object test block?

***Response:*** *We thank Reviewer 3 for this question.* *The Novel Object Test Block always preceded the Familiar Object Test Block because retention of novel label–novel object mappings represented the primary outcome of interest. In addition, novel-object testing provided the most direct assessment of children's learning, as the novel objects did not already have established labels. In this block, children were asked to identify novel objects associated with newly learned labels, whereas the Familiar Object Test Block required children to consider whether a novel label could refer to an object for which they already knew a conventional label. We anticipated that beginning with the novel-object trials would reduce potential confusion and help establish the task demands before children encountered the more challenging familiar-object trials. However, the fixed testing order may have affected performance, particularly in the Familiar Object Test Block. We have, therefore, revised the Discussion (p.31, lines737-746) to acknowledge testing order as a limitation of the current design and to suggest that future studies should consider alternative testing formats that allow novel and familiar referents to be assessed within a single testing phase and use implicit measures of word learning (e.g., eye-tracking).*

19. Was there a reason to separate novel and familiar object testing into two blocks, rather than presenting them within a single trial (with 4 objects presented)? As currently implemented, it may suggest to participants that the same word should refer to both a novel object (in the novel object testing) as well as a familiar object (in the familiar object testing)?

***Response:*** *Reviewer 3 raises an excellent question. Novel-object and familiar-object testing were separated into two blocks to independently assess whether children had a preference to retain novel labels for novel objects or familiar object. This would not have been possible if all four objects were presented in a single trial. We also anticipated children in this age range getting confused when making explicit button-press responses for four choices. Nonetheless, presenting the same novel labels across both testing blocks may have influenced children's interpretation of the task and encouraged consideration of multiple potential referents. We have, therefore, added this as a limitation of the current design and discuss how future studies may address this issue using alternative testing formats and more implicit measures of word learning in the Discussion (p.31, lines741-743).*

20. Table 1: The ordering of indices should be checked.

***Response:*** *We have now rectified this error.*

21. In the description of the GLMM, could the authors a) add the inclusion of the interaction term as a fixed effect to the description and b) specify which kind of GLMM has been performed (e.g., binomial error distribution)?

***Response:*** *We have now added this information (see p.22, lines 540-541)*

22. As a suggestion, while a Wilcoxon test is suitable for this kind of data, the same information could be inferred from a GLMM (i.e., using a consistent approach): e.g., When predicting the outcome by a four-level factor (encompassing the 4 levels accurate-align, inaccurate-align, accurate-conflict, inaccurate-conflict) and removing the intercept, the output would directly reveal the results of testing each of these groups against chance (50%).

***Response:*** *We thank the reviewer for this helpful suggestion. We agree that performance relative to chance could also be examined within a GLMM framework using a four-level factor representing the four learning conditions. However, our primary GLMM analyses were designed to evaluate the factorial structure of the design, that is, the effects of speaker accuracy, learning condition, and their interaction at the trial level. In contrast, the Wilcoxon signed-rank tests addressed a separate question: whether performance within each individual learning condition differed from chance. We chose to retain this approach because it provides a straightforward and commonly used method for evaluating chance-level performance (Crespo & Kaushanskaya, 2021; Gangopadhyay & Kaushanskaya, 2021,2022; Wojcik, 2019) and complements the trial-level GLMM analyses.*

23. My main concern in this section relates to the interpretation of the GLMM: Because the model includes an interaction term, the main effects are conditional on the value of the other predictor (i.e., they refer to the other predictor being 0). Given the chosen coding (centered predictors), the reported main effects reflect effects at the average level of the other predictor (i.e., at a very specific value of the other predictor), which is not equivalent to a global main effect. In the current form of the manuscript, this distinction is not made explicit, although it changes how the results are interpreted and might have implications for the study’s implications. If the goal is to test global main effects, these should be evaluated directly – either by fitting a second model without the interaction term (after finding it to be non-significant) and interpreting the main effects directly, or via model comparisons (models with vs. without the main effect of interest).

***Response:*** *We thank the Reviewer for this thoughtful observation. We agree that, when interaction terms are included in a model, the interpretation of fixed effects requires clarification. In the present analyses, we used contrast coding for speaker accuracy (Accurate = -0.5, Inaccurate = 0.5) and learning condition (Align-with-ME = -0.5, Conflict-with-ME = 0.5), such that the reported fixed-effect estimates represent effects evaluated at the average level of the other predictor (i.e., the main effect). The interaction between speaker accuracy and learning condition was retained because it was a theoretically motivated component of the planned model. Because the interaction between speaker accuracy and learning condition was not significant in either model, our substantive conclusions remained unchanged. However, to improve clarity on the analytical model, we have revised the Results section to explicitly describe the interpretation of the fixed effects under contrast coding (p. 24, lines 584-586 and p.25, lines 608-609)*

24. Age as predictor: Could the authors clarify why age was excluded from the analyses due to non-significance (and not either kept as a control variable, or even kept as a variable that potentially interacts with the effects of interest)?

***Response:*** *We thank Reviewer 3 for this question.* *Given that the sample spanned ages 4–7 years, we evaluated the first model with Age(centered) as a covariate. Age did not significantly predict performance, did not improve model fit (AIC was lower for the models without Age), and did not alter the pattern of fixed effects. Therefore, we retained the simpler model without Age in the final analyses. We have clarified this rationale in the Results section.*

25. Figures 5 & 6: It would be very nice and more informative to present the data at the level of individual participants (e.g., by showing single dots) rather than aggregated bars.

***Response:*** *We have now added data at the level of individual participants using scatter plots.*

26. Further, as a suggestion, the authors could consider combining Figures 5 and 6 into a single (2x2) figure – since the conditions are later compared, presenting them jointly would facilitate visual comparison. And finally, it would be helpful to specify what the error bars represent (if they are retained).

***Response:*** *We have now combined Figures 5 and 6 as suggested and revised the figures to show data at the individual level.*

27. p. 25 “Children’s below-chance performance”: Was the performance really below chance or rather “not above chance?

***Response:*** *We agree that the original wording was imprecise. We have revised the text to clarify that children's performance in this condition was not above chance.*

28. p. 25 “…possible that children assigned the novel label to the familiar object instead”: If that was the case, wouldn’t we see that in their performance in the familiar object testing?

***Response:*** *We agree with the Reviewer that the original interpretation was not fully supported by the pattern of results, as children did not perform above chance during familiar-object testing in the Conflict-with-ME condition. We have, therefore, revised this section (see p.28, lines 684-689) to avoid suggesting that children systematically assigned the novel label to the familiar object. Instead, we now discuss the possibility that conflicting cues disrupted learning, resulting in uncertainty about the intended referent and a failure to establish stable word–object mappings.*

29. p. 26 “older children, with more developed metacognitive and linguistic skills, may rely less on speaker accuracy”: Could the authors elaborate further, why it is plausible to expect an effect in this direction (although sensitivity to speaker reliability is generally rather assumed to increase with age)? Elaborate, why?

***Response:*** *We agree that the original wording could be interpreted as suggesting that sensitivity to speaker accuracy decreases with age, which was not our intention. We have revised the Discussion ( p.29, lines 711-718) to now discuss the possibility that older children may integrate speaker accuracy with other available cues, such as lexical expectations and repeated label–object pairings (to infer from cross-situational statistics), when forming label–object mappings. This interpretation is more consistent with the broader literature on selective trust and cue integration during word learning.*

30. p. 27 “This ordering was intentional, as presenting familiar-object trials first could prime children …”: Could the authors explain why an interference in this (prevented) direction would be less worrying than in the other direction?

***Response:*** *We appreciate this thoughtful observation by Reviewer 3.* *Our rationale for presenting the Novel Object Test Block first was that it provided the more intuitive testing situation for children. We agree, however, that the fixed testing order may have influenced performance in the subsequent familiar-object block. We have, therefore, revised the Discussion to acknowledge this possibility and discuss testing order as a limitation of the current design (see p.31 lines 737-745).*
