# Supplementary material for: The role of speaker accuracy and mutual exclusivity in children’s novel word learning
Source: PLoS One. 2026 Oct 29;21(10):e0361483. doi: 10.1371/journal.pone.0361483 (PMC13718952; doi:10.1371/journal.pone.0361483)
Supplement: Supplementary file 6 [file pone.0361483.s003.docx]

**Title: The role of speaker accuracy and mutual exclusivity in children’s novel word learning**

**Overview**

This study is examining the extent to which 4-to-7 year-old monolinguals weigh mutual exclusivity (ME) versus speaker testimony when learning novel words. Learners were exposed to four conditions: accurate speaker in align-with-ME, inaccurate speaker in align-with-ME, accurate speaker in conflict-with-ME, inaccurate speaker in conflict-with-ME. Findings suggest that children learned better when ME wasn’t violated, regardless of speaker accuracy history.

This is a novel and important contribution to the literature, and fits the scope of the journal. The introduction and the design are especially robust. I have a few comments on the analyses that should only require a minor revision.

**Major comments**

Analyses:

- Why not add item as a random effect as well since the analyses are said to be done at the trial level on line 481?
  🡪 The detailed response fully addressed my initial concern, thank you.
- Did you remove by-participant slopes because of convergence and singularity issues? If so, could you add a sentence to describe this?
  🡪 Same comment, here, thank you.
- Why is the estimate negative for the main effect of condition in the Novel object test trials GLMM? If Align with ME is coded as 0.5 and that learning was higher in that condition than in the Conflict with ME, shouldn’t the estimate be positive (and vice-versa for the next GLMM)?
  🡪 Thank you for checking this and editing the coding reporting.
- What was the rationale for having two sessions? Was it because this project is part of a larger one or was there a specific rationale? Either way, it would be helpful to specify this.
  🡪 Perfect, thank you for clarifying this.
- In the description of the testing phase, I am confused with these sentences: “Each novel label was tested twice, once in each of the two blocks. This resulted in 8 trials per testing block.” Aren’t there only 2 blocks, novel object block and a familiar object block? Therefore, “tested once in each block” would mean 4 trials across both sessions, not 8? Perhaps, the sentence should be corrected to “Each **of the 4** novel label**s** (**accurate or inaccurate, in align-with ME or conflict-with ME**) was tested twice, ~~once in each of the two~~ per blocks. This resulted in 8 trials per testing block.”
  🡪 Same comment, thank you.

**Minor comments**

Thank you for addressing all the comments in this section, I have no further comments.
