# Supplementary material for: The role of speaker accuracy and mutual exclusivity in children’s novel word learning
Source: PLoS One. 2026 Oct 29;21(10):e0361483. doi: 10.1371/journal.pone.0361483 (PMC13718952; doi:10.1371/journal.pone.0361483)
Supplement: Supplementary file 7 [file pone.0361483.s006.docx]

We thank the Editor, the two anonymous reviewers and Dr. Chan for their helpful suggestions on the improvement of our manuscript PONE-D-26-10165R1 “The role of speaker accuracy and mutual exclusivity in children’s novel word learning” Below is a point-by-point response (in italics) of all the changes we have made to the manuscript as a result of the comments and the suggestions made by the Editor and the Reviewers. We have also tracked our revisions in the manuscript, highlighted in yellow.

**Reviewer #1**

Thank you for addressing all the comments in this section, I have no further comments.

***Response:*** We appreciate the Reviewer’s acknowledgment that our responses and revisions have addressed their previous comments and concerns. We thank the Reviewer for their helpful feedback throughout the review process.

**Reviewer #2 Dr. Chan**

The authors have substantially revised the manuscript to address many of my concerns to satisfaction and clarified several things that were unclear in the previous version. Overall, the manuscript has improved in readability, clarity, and coherence. I only have the following, mostly minor, comments/suggestions.

**Response:** We are grateful to Dr Chan for their careful review of the revised manuscript and are happy that our revisions addressed many of their previous concerns. We also thank Dr Chan for the additional comments and suggestions to further improve the manuscript. We have addressed each of these comments below.

Below are responses to substantial minor comments. All other editorial suggestions and corrections have been incorporated into the manuscript, highlighted in yellow.

1. (line 55) The acronym “ME” is defined here, please do not define this again later on. Also, please replace all subsequent mention of “mutual exclusivity” with the acronym.

**Response:** We have made the suggested changes throughout the manuscript.

2. (lines 61-62) “In contrast” here does not really make sense, please replace it with another adverbial.

**Response:** We have replaced “In contrast” with “On the other hand” as suggested (see p. 3, lines 61–62).

3. (line 85) I find the use of “immediately” here problematic and inaccurate. Perhaps replace it with “following a short delay”?

**Response:** We have replaced “immediately” with “following a short delay” as suggested (see p. 4, line 85).

4. (line 87) Here and in the rest of the manuscript, please qualify “retention” with “short-term” for clarity – to avoid confusion with the typical use of the word to refer to longer-term retention in the literature. In some instances (e.g., line 117), this would mean replacing “immediate” with “short-term”.

**Response:** We have incorporated the term “short-term retention” throughout the manuscript where appropriate.

5. (line 119) Please clarify what “looking patterns were faster” means. Was it quicker fixation to the target object or more frequent switching between target and distractor objects?

**Response:** We have clarified that “faster looking patterns” referred to quicker fixation to the target object (see p. 5, line 121).

6. (lines 131-132) The “inconsistent findings” discussed here do not seem to follow from what was discussed in the previous paragraph(s). Please explain what was inconsistent in the literature.

**Response:** We have revised this section to clarify the mixed findings in the literature. Specifically, we now note that some studies have found a preference for accurate speakers during referent selection and retention, whereas others have found that children can learn from both accurate and inaccurate speakers, with differences instead emerging in the speed of looking toward labeled objects (see p. 6, lines 132–136).

7. (line 147) Some studies have shown that children can use ME to learn the meaning of words at earlier ages: Mather and Plunkett (2010) at 10 months and Halberda (2003) at 17 months [reference no. 16].

**Response:** We have replaced the previous statement with the following: “Some studies have shown that children can use ME to learn the meaning of words at earlier ages: Mather and Plunkett (2010) at 10 months and Halberda (2003) at 17 months.” as suggested (see p. 7, lines 150–151).

8. (lines 171-177) To help with the flow and reduce redundancy, I suggest replacing the first sentence in the subsequent section (lines 179-180) with this paragraph. Also, I suggest omitting “, in the service of word learning” in line 173 and inserting a citation of Hollich et al. (2000) [reference no. 3] in line 175.

**Response:** We have made the suggested revisions (see p. 8, lines 176–181).

9. (line 249) The writing “deciding which label-object associations to retain” sounds to me that the children have explicit control over which cues to use when these are typically conceptualised as implicit. Please rephrase for clarity.

**Response:** We have rephrased this sentence to clarify that children form and retain label–object associations when integrating multiple sources of information, rather than explicitly deciding which associations to retain (see p. 11, lines 246–248).

10. (line 573) My understanding is that the journal does not permit footnotes, so please briefly summarise the content of the footnote here.

***Response:*** *We appreciate Dr. Chan for pointing this out. We have now added the information that was in the footnote to the main text (see p.24, lines 566-569).*

11. (Table 2) The numbers in the table do not match those presented in the main text. Also, please include information on random effects. It would also be helpful to present 95% confidence intervals for the fixed effects.

***Response:*** *We thank Dr. Chan for catching this error. The numbers in the table now match those in the text. We have also added information on random effects and confidence intervals for the fixed effects.*

12. (Table 3) Similar to Table 2, please include information on random effects and provide 95% confidence intervals for the fixed effects.

***Response:*** *We have now added this information.*

13. In general, please provide exact p-values to 3 decimal places whenever possible (i.e., except for < .001) and other test statistics to 2 decimal places.

***Response:*** *We have now done so throughout the manuscript.*

14. (lines 794-718) The discussion here seems to point towards an age by speaker accuracy effect, which could have been included in the GLMM analyses. What I suggest here is to say, if accurate, that the current sample did not provide sufficient power to test this interaction and then suggest this for future work.

**Response:** We thank Dr. Chan for this suggestion. We have now mentioned this limitation in the Discussion and noted that future studies with larger samples could directly examine whether the influence of speaker accuracy on word learning changes with age (see p. 30, lines 716–719). Consequently, we have removed “*According to multiple-cue accounts of word learning, children weigh and combine information from a variety of sources, including speaker characteristics, lexical expectations, and distributional information available within the learning environment [42-43]. In the present study, repeated label–object pairings and children's existing lexical knowledge may have provided additional information that influenced learning outcomes alongside speaker accuracy.”*

15. (lines 727-730) This newly added information breaks the flow of the paragraph. I suggest moving this to the end of the paragraph, and instead of “recognize”, say “further note”.

**Response:** We have revised this section as suggested (see p. 31, lines 737–740).

16. (line 746) Please add brief information on what implicit measures of word learning can show us.

**Response:** We have added brief explanation of what implicit measures of word learning can reveal (see p. 31, lines 744–748), namely, that implicit measures can provide insight into the real-time dynamics of children’s recognition of newly learned label–object associations.

**Reviewer #3**

The majority of the points I raised in the first round have been addressed satisfactorily. I have a smaller number of remaining comments, most of which are minor and concern points that either emerged while reading the revised manuscript or that concern a clarification of comments I raised in the first round. I outline these points below.

**Response:** We are grateful to Reviewer 3 for their careful review of the revised manuscript and for their positive comments on the improvements made to the manuscript. We also thank them for the additional comments and suggestions to further strengthen the manuscript. We have addressed each of these comments below.

1. The revised abstract is much clearer and, in my view, now provides a good overview of the study. One point that remained somewhat unclear to me concerns the final conclusion (“that the relative weighting of these cues may vary depending on the learning context”) – could the authors clarify how this conclusion follows from the reported findings?

**Response:** We have now clarified the conclusion of the Abstract to more directly reflect the reported findings (see p. 2, lines 45–47). We now specifically say tha*t children’s short-term retention of label–object associations is supported when available cues align, irrespective of speaker accuracy status. These associations, however, become less robust when ME and speaker cues provide conflicting information.*

2. The revised manuscript now makes the focus on children’s (short-term) retention across different learning contexts much clearer. I noticed, however, a slight asymmetry in the literature reviewed: The section on speaker accuracy discusses previous findings concerning both referent selection and retention, whereas the section on ME appears to focus primarily on children’s referent selection. The authors may decide whether they prefer to retain this focus, or whether they want to mention previous empirical findings on retention of word-object mappings established through ME in this section.

***Response:*** We thank the Reviewer for noting this distinction. In the section on speaker accuracy, we discuss studies demonstrating mixed findings regarding children’s preference for accurate versus inaccurate speakers, which include evidence from both referent selection and short-term retention tasks. In the ME section, our primary goal was to establish ME as an important cue that children use during word learning and to highlight the flexibility with which this cue is applied across different learning contexts. We therefore retained the current focus of this section and discuss evidence concerning short-term retention in the subsequent section on the combined influence of speaker accuracy and ME, which is more directly relevant to the present study.

3. p. 22: I think the reading flow might be slightly improved if the model itself were introduced and explained first, followed by the explanation of why other random effects were not included.

***Response:*** *We have now introduced the model first, followed by information on the final model, as suggested (see p.22-23, lines 530-543).*

4. One remaining point concerns the explanation of the GLMM coefficients at the beginning of the Results section (based on a comment I raised in the first round). I appreciate that the authors have added a clarification in response to my previous comment. However, I think the current wording could still be made somewhat clearer. The authors state that “Because speaker accuracy and learning condition were contrast coded (-0.5, 0.5), fixed-effects estimates are interpreted at the average level of the other predictor. Thus, the reported effects reflect average differences across levels of the other factor rather than effects at a specific reference category”. I have two concerns with the phrasing of these sentences:

a. This interpretation does not concern all “fixed-effects estimates”/ “the reported effects”, but specifically the lower-order terms for condition and speaker accuracy, as these predictors are also included in the interaction.

b. Further, referring to the “average level” of a categorical factor might be somewhat confusing, as there is no actual intermediate level between, e.g., an accurate and an inaccurate speaker (instead, the effect of, e.g., condition is the average of the effect of condition for accurate speakers and the effect of condition for inaccurate speakers). Therefore, it would be clearer to explain directly that, with the chosen contrast coding, the reported effect of condition represents the difference between conditions averaged equally across the two levels of speaker accuracy – and vice versa for the effect of speaker accuracy. (This distinction is particularly relevant if the numbers of observations across the two levels are not equal, because the model coefficient then does not correspond to simply collapsing the observed data across speaker accuracy.)

***Response:*** *We thank Reviewer 3 for the valuable suggestions in numbered points 4a and 4b, and we respond to these together here.* We agree that our previous wording was imprecise in referring broadly to all fixed-effect estimates and to the “average level” of the other predictor. We have revised the text to clarify that this interpretation applies specifically to the lower-order effects of learning condition and speaker accuracy (see p.24, lines 584-588). We now state that, with the chosen contrast coding, the lower-order effect of learning condition represents the difference between conditions averaged equally across the two levels of speaker accuracy, and the lower-order effect of speaker accuracy represents the difference between speaker accuracy levels averaged equally across the two learning conditions.

5. I noticed that some of the numerical values reported in the main text of the Results section are not identical to the ones reported in Table 2.

***Response:*** *We thank the Reviewer for identifying this error, which was also caught by Dr. Chan (Reviewer 2). We have now ensured that values in the table match those in the main text.*

6. In response to one of my comments from the first round, the authors state that “We have now combined Figures 5 and 6 as suggested”. In the revised manuscript, however, the two figures still appear separately. I was wondering whether an outdated version of the figure has been uploaded, or alternatively whether the response letter was not updated to reflect the final revision?

**Response:** We thank the Reviewer for noting this discrepancy and apologize for the misunderstanding. Indeed, an outdated version of the figure was previously uploaded. We have now combined Figures 5 and 6 into a single figure (Figure 5), as originally intended and indicated in our previous response.

7. Finally, in my previous review I suggested specifying in the captions of Figures 5 and 6 what the error bars represent (e.g., SE, SD, 95% confidence intervals etc.). This information still appears to be missing. Although this is a minor point, I think it would be useful to add so that the figures can be interpreted unambiguously.

**Response:** We thank the Reviewer for noting this. We have now clarified in the caption of the combined figure (Figure 5) that the error bars represent standard errors of the mean.

8. Discussion - The authors have clearly made an effort to incorporate the additional discussion points raised during the first round of review. I found these additions very valuable. In a few places, however, the transitions between the original discussion and newly added material felt somewhat abrupt.
   a. E.g., p. 30: “some children associated the test speaker’s voice with one of the speakers from the teaching phase” – the transition to this point could perhaps be made a little more fluent, and it might be helpful to briefly spell out the implication of this possibility.
   b. E.g., p. 33/34: the part beginning with “It is also important to note …” might work better as a separate paragraph. At present, both the transition into this point and the transition from it to the concluding sentence felt somewhat abrupt.

**Response:** We thank the Reviewer for these helpful suggestions (for 8a and 8b). We have revised the transitions and overall flow of the Discussion.

9. Reference 71 appears to contain a short paragraph that may have been intended as a footnote?

**Response:** We thank the Reviewer for noting this. This paragraph was initially intended as a footnote. We have instead incorporated this explanation into the main text, as recommended by Dr. Chan (Reviewer 2).
