# Supplementary material for: The role of speaker accuracy and mutual exclusivity in children’s novel word learning
Source: PLoS One. 2026 Oct 29;21(10):e0361483. doi: 10.1371/journal.pone.0361483 (PMC13718952; doi:10.1371/journal.pone.0361483)
Supplement: Supplementary file 8 [file pone.0361483.s007.docx]

We thank the Dr. Chan and Reviewer 3 for their helpful suggestions on the improvement of our manuscript. Below is a point-by-point response (in italics) to all the comments and the suggestions made by the Reviewers. We have also tracked our revisions in the manuscript, highlighted in yellow.

**Reviewer #2 Dr. Chan**

Most of my comments from the previous round of review have been addressed to satisfaction. I only have a few very minor suggestions.

***Response:*** We are grateful to Dr. Chan for their careful review of the revised manuscript and are happy that our revisions addressed most of their comments. We also thank Dr. Chan for the additional suggestions to further improve the manuscript.

1. (line 55) ‘ME’ should be spelt out in full here as it is the first time this acronym is used after the Abstract. Sorry if this was not clear in my previous comment.

**Response:** We have replaced “ME” by spelling it out as “mutual exclusivity (ME)” (p. 3, line 55), as this is the first time the acronym appears in the manuscript after the Abstract.

2. (line 83) Here, I think it would be helpful to mention that some studies have looked at long-term retention (delay of 5-10 minutes), to highlight the need to study short-term retention.

**Response:** We thank Dr Chan for this suggestion. We have now revised this section to note that some studies of word learning have examined retention following longer delays (Chan & Monaghan, 2025; Horst & Samuelson, 2008; Vlach & DeBrock, 2019), while highlighting the need to examine short-term retention when speaker accuracy and ME are considered together (p. 4, lines 83–85).

3. (lines 150-151) This first sentence may be better suited in line 161, before “However”.

**Response:** We have added “*Some studies have shown that children can use ME to learn the meaning of words at earlier ages – Mather and Plunkett (2010) at 10 months and Halberda (2003) at 17 month*s”, before “however” as suggested (see p. 7, lines 162–163).

4. (line 187) Please use the past tense ‘provided’ instead.

**Response:** We have replaced “provide” with “provided” as suggested (see p.8, line 189).

5. (line 207) Please use the plural ‘speakers’.

**Response:** We have replaced “speaker” with “speakers” as suggested (see p.9, line 209).

6. (line 293) Full stop missing at the end of this line.

**Response:** We have added a period at the end of this line (see p.12, lines 295).

7. (line 530) Wilcoxon signed-rank test is not descriptive. I would omit ‘descriptive analyses’ here to avoid confusion.

**Response:** We have removed “descriptive” and now say “Following these analyses” (see p.22, lines 532).

8. Please follow APA conventions and be consistent in including leading zeroes in the main text and in the tables for Ms, SDs, bs, zs, ps, and SEs.

**Response:** We have revised the main text and tables to ensure that leading zeroes are reported consistently according to APA conventions (and exact p-values to three decimal places).

9. Please report exact p-values to 3 decimal places in the Chance performance analysis section.

**Response:** We have revised the Chance performance analysis section to report exact p-values to three decimal places.

10. (line 599) The SE reported here is still different from that in Table 2.

**Response:** We appreciate Dr. Chan for pointing this out. This was a typing error, and we have now corrected the SE in the main text to 0.42 to match Table 2.

11. (line 764) Please replace ‘effect’ with ‘affect’.

**Response:** We have replaced “effect” with “affect” as suggested (see p.32, line 768).

**Reviewer #3**

The authors have addressed the great majority of the points I raised in the previous round, and I think the revisions have clearly improved the manuscript in terms of readability and clarity. I only have two very minor remaining requests for clarification, both relating to comments from the previous round. Beyond these points, I have no further comments.

**Response:** We are grateful to Reviewer 3 for their careful review of the revised manuscript and for their positive comments on the improvements made to the manuscript. We also thank them for the additional suggestions to further strengthen the manuscript. We have addressed both their comments below.

1. Following the revisions, the GLMMs and their interpretation are now described very clearly, particularly on p. 24. On p. 25, in the sentence “As in the novel-object analyses, fixed-effect estimates are interpreted at the average level of the other predictor due to contrast coding,” the authors could simply clarify that this refers to the “lower-level fixed-effect estimates” or, more specifically, to the “fixed-effect estimates of condition and accuracy,” as they do on p. 24.

**Response:** We have revised the sentence as suggested to clarify that this interpretation refers specifically to the fixed-effect estimates of condition and accuracy (see p. 25, lines 611–613).

2. Table 2 still contains one value that differs from the corresponding value reported in the text: the SE for the interaction term.

**Response:** We thank the Reviewer for identifying this discrepancy, which was also noted by Dr. Chan (Reviewer 2). We have corrected the SE for the interaction term and ensured that the values in Table 2 match those reported in the main text.
