# Supplementary material for: The role of speaker accuracy and mutual exclusivity in children’s novel word learning
Source: PLoS One. 2026 Oct 29;21(10):e0361483. doi: 10.1371/journal.pone.0361483 (PMC13718952; doi:10.1371/journal.pone.0361483)
Supplement: Peer review history

# Peer review: Decision Letter 0

**DOI:** 10.1371/journal.pone.0361483.r001

**Authors:** Jie Wang

5 May 2026

PONE-D-26-10165The role of speaker accuracy and mutual exclusivity in children’s novel word learningPLOS One

Dear Dr. Banerjee,

Thank you for submitting your manuscript to PLOS ONE. After careful consideration, we feel that it has merit but does not fully meet PLOS ONE’s publication criteria as it currently stands. Therefore, we invite you to submit a revised version of the manuscript that addresses the points raised during the review process.

Please submit your revised manuscript by Jun 19 2026 11:59PM. If you will need more time than this to complete your revisions, please reply to this message or contact the journal office at plosone@plos.org. When you're ready to submit your revision, log on to https://www.editorialmanager.com/pone/ and select the 'Submissions Needing Revision' folder to locate your manuscript file.

Please include the following items when submitting your revised manuscript:

- A letter that responds to each point raised by the academic editor and reviewer(s). You should upload this letter as a separate file labeled 'Response to Reviewers'.
- A marked-up copy of your manuscript that highlights changes made to the original version. You should upload this as a separate file labeled 'Revised Manuscript with Track Changes'.
- An unmarked version of your revised paper without tracked changes. You should upload this as a separate file labeled 'Manuscript'.

If you would like to make changes to your financial disclosure, please include your updated statement in your cover letter. Guidelines for resubmitting your figure files are available below the reviewer comments at the end of this letter.

If applicable, we recommend that you deposit your laboratory protocols in protocols.io to enhance the reproducibility of your results. Protocols.io assigns your protocol its own identifier (DOI) so that it can be cited independently in the future. For instructions see: https://journals.plos.org/plosone/s/submission-guidelines#loc-laboratory-protocols. Additionally, PLOS ONE offers an option for publishing peer-reviewed Lab Protocol articles, which describe protocols hosted on protocols.io. Read more information on sharing protocols at https://plos.org/protocols?utm_medium=editorial-email&utm_source=authorletters&utm_campaign=protocols.

As the corresponding author, your ORCID iD is verified in the submission system and will appear in the published article. PLOS supports the use of ORCID, and we encourage all coauthors to register for an ORCID iD and use it as well. Please encourage your coauthors to verify their ORCID iD within the submission system before final acceptance, as unverified ORCID iDs will not appear in the published article. *Only*  the individual author can complete the verification step; PLOS staff *cannot*  verify ORCID iDs on behalf of authors.

We look forward to receiving your revised manuscript.

Kind regards,

Jie Wang, Ph.D.

Academic Editor

PLOS One

Journal Requirements:

When submitting your revision, we need you to address these additional requirements.

1. Please ensure that your manuscript meets PLOS ONE's style requirements, including those for file naming. The PLOS ONE style templates can be found at

https://journals.plos.org/plosone/s/file?id=wjVg/PLOSOne_formatting_sample_main_body.pdf and

https://journals.plos.org/plosone/s/file?id=ba62/PLOSOne_formatting_sample_title_authors_affiliations.pdf

2. Thank you for stating the following in the Acknowledgments Section of your manuscript:

“This research was supported by the Indiana University Bloomington - Department of Speech Language and Hearing Sciences Graduate Research Fund.”

We note that you have provided funding information that is not currently declared in your Funding Statement. However, funding information should not appear in the Acknowledgments section or other areas of your manuscript. We will only publish funding information present in the Funding Statement section of the online submission form.

Please remove any funding-related text from the manuscript and let us know how you would like to update your Funding Statement. Currently, your Funding Statement reads as follows:

“This research was supported by the Indiana University Bloomington - Department of Speech Language and Hearing Sciences Graduate Research Fund.”

Please include your amended statements within your cover letter; we will change the online submission form on your behalf.

3. We note that the grant information you provided in the ‘Funding Information’ and ‘Financial Disclosure’ sections do not match.

When you resubmit, please ensure that you provide the correct grant numbers for the awards you received for your study in the ‘Funding Information’ section.

4. Thank you for stating the following financial disclosure:

“This research was supported by the Indiana University Bloomington - Department of Speech Language and Hearing Sciences Graduate Research Fund.”

Please state what role the funders took in the study.  If the funders had no role, please state: "The funders had no role in study design, data collection and analysis, decision to publish, or preparation of the manuscript."

If this statement is not correct you must amend it as needed.

Please include this amended Role of Funder statement in your cover letter; we will change the online submission form on your behalf.

5. Thank you for stating the following financial disclosure:

“This research was supported by the Indiana University Bloomington - Department of Speech Language and Hearing Sciences Graduate Research Fund.”

We note that one or more of the authors is affiliated with the funding organization, indicating the funder may have had some role in the design, data collection, analysis or preparation of your manuscript for publication; in other words, the funder played an indirect role through the participation of the co-authors. If the funding organization did not play a role in the study design, data collection and analysis, decision to publish, or preparation of the manuscript and only provided financial support in the form of authors' salaries and/or research materials, please do the following:

1. Review your statements relating to the author contributions, and ensure you have specifically and accurately indicated the role(s) that these authors had in your study. These amendments should be made in the online form.

2. Confirm in your cover letter that you agree with the following statement, and we will change the online submission form on your behalf:

“The funder provided support in the form of salaries for authors [insert relevant initials], but did not have any additional role in the study design, data collection and analysis, decision to publish, or preparation of the manuscript. The specific roles of these authors are articulated in the ‘author contributions’ section.

6. PLOS requires an ORCID iD for the corresponding author in Editorial Manager on papers submitted after December 6th, 2016. Please ensure that you have an ORCID iD and that it is validated in Editorial Manager. To do this, go to ‘Update my Information’ (in the upper left-hand corner of the main menu), and click on the Fetch/Validate link next to the ORCID field. This will take you to the ORCID site and allow you to create a new iD or authenticate a pre-existing iD in Editorial Manager.

7. Please include a separate caption for each figure in your manuscript.

8. Please upload a copy of Figures 1 - 6, to which you refer in your text on page 15, 16, 19 and 21. If the figure is no longer to be included as part of the submission please remove all reference to it within the text.

9. Please ensure that you refer to Figures S1 Fig 1 - S6 Fig 6 in your text as, if accepted, production will need this reference to link the reader to the figure.

10. We note that Figures S2 Fig 2 and S3 Fig 3 includes an image of a participant in the study.

As per the PLOS ONE policy (http://journals.plos.org/plosone/s/submission-guidelines#loc-human-subjects-research) on papers that include identifying, or potentially identifying, information, the individual(s) or parent(s)/guardian(s) must be informed of the terms of the PLOS open-access (CC-BY) license and provide specific permission for publication of these details under the terms of this license. Please download the Consent Form for Publication in a PLOS Journal (http://journals.plos.org/plosone/s/file?id=8ce6/plos-consent-form-english.pdf). The signed consent form should not be submitted with the manuscript, but should be securely filed in the individual's case notes. Please amend the methods section and ethics statement of the manuscript to explicitly state that the patient/participant has provided consent for publication: “The individual in this manuscript has given written informed consent (as outlined in PLOS consent form) to publish these case details”.

If you are unable to obtain consent from the subject of the photograph, you will need to remove the figure and any other textual identifying information or case descriptions for this individual.

11. If the reviewer comments include a recommendation to cite specific previously published works, please review and evaluate these publications to determine whether they are relevant and should be cited. There is no requirement to cite these works unless the editor has indicated otherwise.

Additional Editor Comments:

The authors need to carefully address the comments raised by the three reviewers.

[Note: HTML markup is below. Please do not edit.]

Reviewers' comments:

Reviewer's Responses to Questions

**Comments to the Author**

1. Is the manuscript technically sound, and do the data support the conclusions?

The manuscript must describe a technically sound piece of scientific research with data that supports the conclusions. Experiments must have been conducted rigorously, with appropriate controls, replication, and sample sizes. The conclusions must be drawn appropriately based on the data presented.

Reviewer #1: Yes

Reviewer #2: No

Reviewer #3: Yes

**********

2. Has the statistical analysis been performed appropriately and rigorously?

Reviewer #1: Yes

Reviewer #2: No

Reviewer #3: Yes

**********

3. Have the authors made all data underlying the findings in their manuscript fully available?

The PLOS Data policy requires authors to make all data underlying the findings described in their manuscript fully available without restriction, with rare exception (please refer to the Data Availability Statement in the manuscript PDF file). The data should be provided as part of the manuscript or its supporting information, or deposited to a public repository. For example, in addition to summary statistics, the data points behind means, medians and variance measures should be available. If there are restrictions on publicly sharing data—e.g. participant privacy or use of data from a third party—those must be specified.

Reviewer #1: No

Reviewer #2: Yes

Reviewer #3: Yes

**********

4. Is the manuscript presented in an intelligible fashion and written in standard English?

PLOS ONE does not copyedit accepted manuscripts, so the language in submitted articles must be clear, correct, and unambiguous. Any typographical or grammatical errors should be corrected at revision, so please note any specific errors here.

Reviewer #1: Yes

Reviewer #2: Yes

Reviewer #3: Yes

**********

5. Review Comments to the Author

Please use the space provided to explain your answers to the questions above. You may also include additional comments for the author, including concerns about dual publication, research ethics, or publication ethics. (Please upload your review as an attachment if it exceeds 20,000 characters)

Reviewer #1: Yes, the introduction is robust, and so are the design and analyses. The results are well-interpreted. The OSF link is shared but I could not access the files, an error message popped up. The level of English is excellent.

Reviewer #2: The manuscript reports a study that tested 4- to 7-year-olds on a word learning task where they were taught novel labels for novel and familiar objects by two speakers, one who had been shown to be always accurate in naming familiar objects and the other consistently inaccurate. It was found that the children were only able to identify the intended objects for the taught novel labels when the labels were taught for novel objects, regardless of speaker accuracy.

The study built on previous research that has shown children’s learning of novel words are guided by mutual exclusivity and speaker accuracy and explicitly examined performance beyond immediate referent selection with both novel and familiar objects. The topic of the study is interesting and fits with the remit of the journal. However, with the concerns I outline below, the contribution to the literature is very limited in its current form. My major concerns surround the structure of the Introduction, the discussion of the theoretical framework, the design of the study, the one-sample tests, and the interpretation of results.

First, I find the structure of the Introduction rather difficult to follow. There is some unnecessary repetition and the flow between different subsections is not always clear. I suggest streamlining the first two paragraphs and using only one paragraph to provide an overview of the background and state the aim of the study. Details of previous work should be worked into later paragraphs. In places where there are subheadings, links to previous paragraphs should be made explicit to allow for a better flow. Also, some careful thinking is needed to discuss related information closer together to avoid unnecessary repetition. One way would be to look at how citations are repeated throughout the Introduction and bring the discussion around the same citations closer together.

In terms of theoretical framework, the authors outlined the multiple-cue account of word learning. However, often, the authors create a false dilemma between speaker accuracy and mutual exclusivity (e.g., lines 127-128). According to the multiple-cue account, children would draw on any informative cues when learning the meanings of new words and the reliance of different cues may vary across contexts (see Chan & Monaghan, 2025). The study’s focus on two particular cues does not rule out children’s use of other cues. The constant omission of other cues throughout the manuscript is rather problematic and has serious implications for the interpretation of results. In particular, since the pairing of objects in the teaching and test phases was different, correct identification of objects at test could be due to cross-situational statistical learning – this is especially relevant given that the children identified the correct novel objects in the Align-with-ME regardless of speaker accuracy, suggesting that they actually accepted lexical overlap to some extent (based on my understanding that two names were introduced to each object, one by the accurate speaker and one by the inaccurate speaker). The results would be more appropriately interpreted as cues that do not violate established lexical knowledge allowed cross-situational statistics to override mutual exclusivity, regardless of speaker accuracy. And the reason for not observing the same in the Conflict-with-ME condition would be that the introduction of novel labels to objects that the children were overly familiar with confused them, leading to them not learning anything. Hence, the chance-level performance. In short, the authors need to take other cues into account when discussing the literature and interpreting the results.

A major concern about the design is that it is unclear why the task is referred to as a retention/recall task. First, given the timings of the task, the time lag between teaching and test could be as short as 30 s. This is considerably shorter than the conventional delay of at least 5 minutes used in the word-learning literature to ensure retrieval from long-term memory (Horst & Samuelson, 2008). A clear explanation and justification for not using a longer delay is needed for claims to be made about retention/recall. Further, even if the short delay could be seen as involving long-term memory, the lack of an immediate referent selection test makes it impossible to see if the results were truly about poor retention or if they were about poor learning in the first place. A more careful consideration of whether the results speak to learning or retention is needed.

One other question about the task is how distinguishable was the voice of the third speaker in the test phase from those of the first and second speakers in the teaching phase? If the voices could not be clearly distinguised, the children may have attributed the voice in the test phase to one of the previous speakers, which may have implications for their performance.

Regarding the one-sample tests, it appears to me that the reported tests were one-tailed, as the graph for the familiar object trials in the Align-with-ME condition seems to suggest that performance was significantly below chance, but the statistics suggest otherwise. Was there a rationale for conducting one-tailed tests? This has serious implications for the interpretation of results, because if the children performed significantly below chance, it means that they were showing preference for a different object than expected. This would demonstrate associations of ‘not the target’ has been formed, which would be evidence for statistical learning taking place. If this was the case, I wondered if the manipulation of speaker accuracy was relevant at all. It could be that the other cues (i.e., cross-situational statistics and their lexical knowledge) were more salient and the children relied on them solely and disregarded speaker accuracy completely. This would mean that the study did not actually test the effect of speaker accuracy and any claims about speaker accuracy would be unwarranted. In this case, the authors did not really test what they set out to investigate.

Below is a list of other minor concerns:

Abstract

1. It would be good to start with some background information.

2. (line 28) Unnecessary repetition noted.

3. (lines 28-33) The description of the design of the study could be made clearer and more succinct.

Introduction

4. (lines 52-54) This sentence does not seem grammatical. Please rephrase.

5. (lines 70-74) This sentence is utterly difficult to follow. Please check that the information here is accurate and that it follows logically from the previous sentence.

6. (lines 90-91) This sentence is difficult to follow. Please rephrase.

7. (line 97) I suggest replacing ‘a novel object’ with ‘the same novel object’ for clarity.

8. (lines 101-104) The writing here could be clearer. I suggest explicitly comparing against accurate speakers here and replacing ‘correct’ with ‘intended’. Also, it should be made clear in the description of the study that the children were not tested on immediate referent selection, but a slightly delayed identification/recall.

9. (lines 107 and 113) Please replace ‘4-5-year-old’ with ‘4- to 5-year-old’.

10. (line 113) Please explain/elaborate on what is meant by ‘equally well’.

Method

11. (lines 334-335) The information in parentheses should be moved to line 336, after ‘familiar objects’.

12. (lines 405-424) Please provide detailed information on the timings of event. How long were the objects shown before the word was spoken? How long did each trial last?

13. (line 481) Please provide clear justification for running separate GLMMs for the novel and familiar blocks.

Results

14. Please be consistent with formatting when reporting p-values.

General

15. Please make sure to use past tense when discussing previous work.

16. Please do not use ‘significant’ to mean important or substantial – reserve it for statistical significance to avoid confusion.

17. Please use commas appropriately to help with readability and clarity.

18. Please check the reference list. 6 and 13 seem to be the same paper. Also, title case (and the lack of) has not been consistently applied.

Reviewer #3: The present manuscript investigates how speaker accuracy and mutual exclusivity, independently or in interaction, may shape children’s word learning. Based on their findings, the authors conclude that speaker cues, when aligned with mutual exclusivity expectations, facilitate children’s word retention, whereas conflicting information may be disregarded. Overall, the research question is compelling, the idea sound and the design appears in principle suitable to address the proposed questions. After reading the manuscript carefully, my main concerns primarily relate to a) the conceptual and theoretical integration of the research question within the existing literature, b) the clarity and strength of the study’s motivation and rationale, c) the structure of the methodological description, and d) the implementation and interpretation of their statistical analysis. I will explain each of these points, along with additional minor comments, in detail below.

Abstract

- I experienced the writing style of the abstract as somehow segmented and lacking a (short) conceptual introduction of the topic

- “The combined effect of speaker accuracy and mutual exclusivity (ME) was assessed” --> Could the authors define the effect of interest (effect on …?)

- The 2nd and 3rd sentence are redundant

Introduction

- The introduction moves rather quickly to empirical findings. It would benefit from a stronger conceptual and theoretical framing at the outset, including more background and a strong argumentation why the two focal cues (ME and speaker accuracy), and especially their combined effect, are theoretically important.

- Further, the introduction would benefit from a clearer argumentation for the study’s rationale, by highlighting the novel contribution. In particular, if the main aspect of interest is the focus on children’s retention, as opposed to their immediate referent selection, this argumentation needs to be clearer, including the theoretical importance of assessing retention performance, as well as concrete differences to prior studies (e.g., p. 9 “However, this study assessed children’s immediate referent selection rather than the retention” – The authors may want to specify in how far this immediate referent selection differs from the immediate retention in the present study?)

- p. 5 “While children’s looking patterns differed as a function of speaker accuracy” --> How did they differ?

- p. 7 (reference to Kalashnikova et al., 2016): From the current description, it is not entirely clear what the study is intended to illustrate in this context. If the goal of this paragraph is to show that children sometimes suspend ME, there is a substantial body of literature that could support this point more directly: e.g., work by Diesendruck shows that children avoid the ME conclusion if the speaker uses proper nouns instead of conventional words (Diesendruck, 2005), if the speaker doesn’t know the conventional word because they don’t speak the language in question (Diesendruck, 2005) or because they proved to be an inaccurate language user (Diesendruck et al., 2010). This line of work seems highly relevant to the current study and could be incorporated here or elsewhere in the manuscript.

- p. 9: If the distinction between disambiguation and retention is of particular interest for the current study, and since retention is mentioned much earlier, introducing this contrast earlier in the manuscript (and clarifying its theoretical implications and relevance) would improve the structure and clarity of the study motivation.

- p. 10: The section on the “combined impact of speaker accuracy and mutual exclusivity” would benefit from a clearer articulation of what remains unsolved by the current literature to motivate the study.

- p. 11: “In the first block (novel object testing), children were presented with two novel objects …”: Here and in the following sentence, could the authors specify that these refer to the previously presented novel objects and words?

Method

- Overall, the methods appear generally appropriate to address the research question. However, I found the whole section difficult to follow at times, as it currently lacks the level of structure needed for a reader to fully understand the details of this design.

- The formatting and hierarchy of section headers seem inconsistent and might need revision (e.g., “Stimuli” as sub-header of “Measures”; “Recordings” at the same level as “Stimuli”; “Design” as sub-header of “Recordings” & at the same level as the components of the study; no “Procedure”, etc.)

- Further, the ordering of sections seems rather unintuitive and would benefit from a clearer structure (e.g., presenting the Design and Procedure before the Measures would make the manuscript easier to follow; the “Experimental task” is currently listed under Measures (p.14) – it would be more intuitive to describe this information directly before the procedure (i.e., the single components) are described).

- In general, from the description of the procedure and design details, it was challenging to reconstruct the design fully, including the number of trials per condition, session etc., as relevant information is distributed across sections. Providing a clearer overview of the design (both in-text and potentially visually) would greatly improve readability.

- Relatedly, Figures 3 & 4: It would be helpful if the figures illustrated a complete sequence of example trials using consistent stimuli (same objects/ words across figures). This would make it easier to see how different trial types are constituted and relate to each other.

- Figure 3: Could the authors specify whether the information about the number of labels refers to both sessions or only one (and possibly add a similar information to the test trials/ Figure 4)?

- p. 17/18 “Using a non-social attentional cue ensured that any learning differences could be attributed to children’s weighting of speaker accuracy versus ME, rather than to their sensitivity to eye gaze or ostensive signals” --> Could the authors clarify this argument? It is not immediately obvious to me why this decision would produce less differences between tasks than, e.g., the consistent use of speaker pointing/ looking at one object?

- p.19: “This resulted in 8 trials per testing block”: Per block or per condition/ session? A more explicit overview of the procedure earlier in the section would probably help clarify this as well.

- What was the reasoning behind deciding against a delay between the learning and test phase (although retention strictly refers to retrieving information from the long-term memory)?

- Why did the novel object test block always precede the familiar object test block?

- Was there a reason to separate novel and familiar object testing into two blocks, rather than presenting them within a single trial (with 4 objects presented)? As currently implemented, it may suggest to participants that the same word should refer to both a novel object (in the novel object testing) as well as a familiar object (in the familiar object testing)?

- Table 1: The ordering of indices should be checked

- In the description of the GLMM, could the authors a) add the inclusion of the interaction term as a fixed effect to the description and b) specify which kind of GLMM has been performed (e.g., binomial error distribution)?

- As a suggestion, while a Wilcoxon test is suitable for this kind of data, the same information could be inferred from a GLMM (i.e., using a consistent approach): e.g., When predicting the outcome by a four-level factor (encompassing the 4 levels accurate-align, inaccurate-align, accurate-conflict, inaccurate-conflict) and removing the intercept, the output would directly reveal the results of testing each of these groups against chance (50%).

Results

- My main concern in this section relates to the interpretation of the GLMM: Because the model includes an interaction term, the main effects are conditional on the value of the other predictor (i.e., they refer to the other predictor being 0). Given the chosen coding (centered predictors), the reported main effects reflect effects at the average level of the other predictor (i.e., at a very specific value of the other predictor), which is not equivalent to a global main effect. In the current form of the manuscript, this distinction is not made explicit, although it changes how the results are interpreted and might have implications for the study’s implications.

- If the goal is to test global main effects, these should be evaluated directly – either by fitting a second model without the interaction term (after finding it to be non-significant) and interpreting the main effects directly, or via model comparisons (models with vs. without the main effect of interest).

- Figures 5 & 6: It would be very nice and more informative to present the data at the level of individual participants (e.g., by showing single dots) rather than aggregated bars. Further, as a suggestion, the authors could consider combining Figures 5 and 6 into a single (2x2) figure – since the conditions are later compared, presenting them jointly would facilitate visual comparison. And finally, it would be helpful to specify what the error bars represent (if they are retained).

- Age as predictor: Could the authors clarify why age was excluded from the analyses due to non-significance (and not either kept as a control variable, or even kept as a variable that potentially interacts with the effects of interest)?

Discussion

- p. 25 “Children’s below-chance performance”: Was the performance really below chance or rather “not above chance?

- p. 25 “…possible that children assigned the novel label to the familiar object instead”: If that was the case, wouldn’t we see that in their performance in the familiar object testing?

- p. 26 “older children, with more developed metacognitive and linguistic skills, may rely less on speaker accuracy”: Could the authors elaborate further, why it is plausible to expect an effect in this direction (although sensitivity to speaker reliability is generally rather assumed to increase with age)?

- p. 27 “This ordering was intentional, as presenting familiar-object trials first could prime children …”: Could the authors explain why an interference in this (prevented) direction would be less worrying than in the other direction?

**********

6. PLOS authors have the option to publish the peer review history of their article (what does this mean?). If published, this will include your full peer review and any attached files.

If you choose “no”, your identity will remain anonymous but your review may still be made public.

**Do you want your identity to be public for this peer review?**  For information about this choice, including consent withdrawal, please see our Privacy Policy.

Reviewer #1: No

Reviewer #2: No

Reviewer #3: No

**********

[NOTE: If reviewer comments were submitted as an attachment file, they will be attached to this email and accessible via the submission site. Please log into your account, locate the manuscript record, and check for the action link "View Attachments". If this link does not appear, there are no attachment files.]

To ensure your figures meet our technical requirements, please review our figure guidelines: https://journals.plos.org/plosone/s/figures

You may also use PLOS’s free figure tool, NAAS, to help you prepare publication quality figures: https://journals.plos.org/plosone/s/figures#loc-tools-for-figure-preparation.

NAAS will assess whether your figures meet our technical requirements by comparing each figure against our figure specifications.

Attachment Submitted filename: PLOS_040126_R1.docx

# Author comment: Author response to Decision Letter 1

**DOI:** 10.1371/journal.pone.0361483.r002

17 Jul 2026

We thank the three anonymous reviewers for their helpful suggestions on the improvement of our manuscript. Below is a point-by-point response (in italics) to each comment, with “Response:” clearly indicated for easy reference. We have also tracked our revisions in the manuscript, highlighted in yellow.

Reviewer #1

This study is examining the extent to which 4-to-7 year-old monolinguals weigh mutual exclusivity (ME) versus speaker testimony when learning novel words. Learners were exposed to four conditions: accurate speaker in align-with-ME, inaccurate speaker in align-with-ME, accurate speaker in conflict-with-ME, inaccurate speaker in conflict-with-ME. Findings suggest that children learned better when ME wasn’t violated, regardless of speaker accuracy history.

This is a novel and important contribution to the literature and fits the scope of the journal. The introduction and the design are especially robust. I have a few comments on the analyses that should only require a minor revision.

Response: We are grateful to Reviewer 1 for their positive review of the manuscript, and for highlighting the importance of our study. We are especially thankful for the Reviewer’s suggestions to describe our analysis plan in more detail.

Major comments

1. Why not add item as a random effect as well since the analyses are said to be done at the trial level on line 481?

Response: We thank Reviewer 1 for this question. In our initial analysis, we indeed added test word as an item-level random effect. The resulting model produced a singular fit, with negligible variability attributable to individual test words (Variance = 0.00, SD = 0.00). In addition, inclusion of the item random effect did not improve model fit relative to the participant random-intercept model (AIC = 521.20 vs. 519.20, respectively), and the fixed-effect estimates remained unchanged. Therefore, item as a random effect was removed from the final model.

We have updated the Analyses section (p.22-23, lines 534-549) to describe the model comparison procedure and rationale for retaining the simpler model. The revised R analysis script has also been included in the OSF repository (https://osf.io/5x2ze/files/osfstorage).

2. Did you remove by-participant slopes because of convergence and singularity issues? If so, could you add a sentence to describe this?

Response: We thank the Reviewer for this question. The Reviewer is correct. We evaluated models with random slopes for the main effects of speaker accuracy and learning condition, as well as their interaction. However, these resulted in singular fits and negligible differences in model fit relative to the simpler participant random-intercept model (e.g., AIC = 518.86 vs. 519.20).

The Analyses section (p.23, lines 547-549) has been updated to clarify the model comparison procedure, final random-effects structure, and model specification.

3. Why is the estimate negative for the main effect of condition in the Novel object test trials GLMM? If Align with ME is coded as 0.5 and that learning was higher in that condition than in the Conflict with ME, shouldn’t the estimate be positive (and vice-versa for the next GLMM)?

Response: We are grateful to Reviewer 1 for identifying this discrepancy. We incorrectly described the contrast coding scheme in the manuscript, although the analyses themselves were conducted using the correct coding specification, that of Align as -0.5 and Conflict as 0.5.

We have now revised the contrast coding description in the Analyses section (p.22-23, lines 544-546) to accurately reflect the coding used in the analyses and reported results.

4. What was the rationale for having two sessions? Was it because this project is part of a larger one or was there a specific rationale? Either way, it would be helpful to specify this.

Response: We thank the reviewer for this helpful suggestion.There were methodological and practical reasons for conducting the study across two sessions. Because the study employed a within-subjects design (i.e., all children completed both Align-with-ME and Conflict-with-ME learning conditions), separating the conditions across two sessions would minimize confusion between learning conditions and speaker accuracy status. Moreover, the study included the word learning task as well as standardized assessments; dividing participation across two visits reduced burden and fatigue for young children.

We have included a rationale for conducting the study over two sessions in the Procedure section (p.14, lines 323-326).

5. In the description of the testing phase, I am confused with these sentences: “Each novel label was tested twice, once in each of the two blocks. This resulted in 8 trials per testing block.” Aren’t there only 2 blocks, novel object block and a familiar object block? Therefore, “tested once in each block” would mean 4 trials across both sessions, not 8? Perhaps, the sentence should be corrected to “Each of the 4 novel labels (accurate or inaccurate, in align-with ME or conflict-with ME) was tested twice, per blocks. This resulted in 8 trials per testing block.”

Response: We thank the reviewer for catching this error, and we agree that the original wording was unclear. We have revised the description of the testing phase to clarify that each of the four novel labels was tested twice, per block, resulting in eight trials per testing block (p.21, lines 492-495).

Minor comments

6. The second sentence of the abstract is repeated twice.

Response: We have rectified this error.

7. Small detail: the recruitment dates could be written with the month in letters – I read the first one as 10th Jan. but based on the second one, it’s probably 1st Oct.

Response: We have now done so (p.13, line 318).

8. I was not able to access the OSF files; an error message popped up, at least for the .xlsx files (I did not try the R files).

Response: We thank the reviewer for bringing this issue to our attention. We have now updated the repository permissions, verified access to all uploaded materials, and re-uploaded the relevant files. In addition, we have added the revised R analysis script and session counterbalancing scheme to the repository for transparency. All files should now be accessible through the OSF link provided in the manuscript.

Reviewer #2

My major concerns surround the structure of the Introduction, the discussion of the theoretical framework, the design of the study, the one-sample tests, and the interpretation of results.

Response: We thank Reviewer 2 for their thoughtful and constructive feedback on our manuscript. We have carefully revised the manuscript to strengthen the conceptual framing of the study, clarify terminology, provide additional methodological details and justifications, and refine our interpretation of the findings within a broader multiple-cue framework. We have also improved the overall organization and flow of the manuscript.

Major Comments

1. I suggest streamlining the first two paragraphs and using only one paragraph to provide an overview of the background and state the aim of the study.

Response: We thank the reviewer for this helpful suggestion. We have now revised the original first two paragraphs to provide a more concise overview of the theoretical background and study rationale.

2. Details of previous work should be worked into later paragraphs. In places where there are subheadings, links to previous paragraphs should be made explicit to allow for a better flow. Also, some careful thinking is needed to discuss related information closer together to avoid unnecessary repetition. One way would be to look at how citations are repeated throughout the Introduction and bring the discussion around the same citations closer together.

Response: We appreciate Reviewer 2 for this valuable suggestion. We have reorganized the Introduction to improve its overall flow. We have provided clearer transitions between subsections to explicitly connect the discussion of speaker accuracy, mutual exclusivity, and their combined role in word learning.

3. In terms of theoretical framework, the authors outlined the multiple-cue account of word learning. However, often, the authors create a false dilemma between speaker accuracy and mutual exclusivity (e.g., lines 127-128). According to the multiple-cue account, children would draw on any informative cues when learning the meanings of new words and the reliance of different cues may vary across contexts (see Chan & Monaghan, 2025). The study’s focus on two particular cues does not rule out children’s use of other cues. The constant omission of other cues throughout the manuscript is rather problematic and has serious implications for the interpretation of results. In particular, since the pairing of objects in the teaching and test phases was different, correct identification of objects at test could be due to cross-situational statistical learning – this is especially relevant given that the children identified the correct novel objects in the Align-with-ME regardless of speaker accuracy, suggesting that they actually accepted lexical overlap to some extent (based on my understanding that two names were introduced to each object, one by the accurate speaker and one by the inaccurate speaker).

Response: We thank the Reviewer for this insightful comment. We recognize that we may have framed speaker accuracy and mutual exclusivity as competing cues in some sections of the manuscript, which is inconsistent with a multiple-cue account of word learning. In response, we have revised the Introduction and Discussion to more explicitly acknowledge that children draw on multiple sources of information during word learning and that the relative weighting of these cues may vary across learning contexts. We have also removed language that could be interpreted as children relied on speaker accuracy or mutual exclusivity in isolation and instead emphasize how these cues may operate alongside other sources of information.

Furthermore, we have expanded on this as a limitation in the Discussion section to indicate that children’s performance may have been influenced by other cues not directly examined in the present study, including cross-situational statistical learning. We now discuss the possibility that children integrated multiple cues when learning and retrieving novel word–object associations and note that future research should further investigate the relative contribution of these cues to word learning outcomes (p.32, lines763-777).

4. A major concern about the design is that it is unclear why the task is referred to as a retention/recall task. First, given the timings of the task, the time lag between teaching and test could be as short as 30 s. This is considerably shorter than the conventional delay of at least 5 minutes used in the word-learning literature to ensure retrieval from long-term memory (Horst & Samuelson, 2008). A clear explanation and justification for not using a longer delay is needed for claims to be made about retention/recall. Further, even if the short delay could be seen as involving long-term memory, the lack of an immediate referent selection test makes it impossible to see if the results were truly about poor retention or if they were about poor learning in the first place. A more careful consideration of whether the results speak to learning or retention is needed.

Response: We thank the Reviewer for this comment. We agree that the distinction between immediate referent selection, learning, and retention should be articulated more clearly (a similar concern raised by Reviewer 3). Our intention was not to make claims about long-term retention or retrieval from long-term memory, but rather to examine children's ability to retain newly learned word–object associations following completion of the teaching phase. We use the term retention to distinguish performance during the testing phase from immediate referent selection (i.e., disambiguation). Prior work has emphasized that successful referent selection does not necessarily result in stable word learning and that word learning is a dynamic, multi-stage process that extends beyond the initial mapping of a label to its referent (e.g., Horst & Samuelson, 2008; McMurray et al., 2012). Consistent with this distinction, several studies have assessed children's ability to retrieve newly learned word–object mappings across multiple trials following completion of the learning phase and have referred to this process as immediate retention, including when testing occurred within the same experimental session (e.g., Bion et al., 2013; Escobar et al., 2023; Pomper & Saffran, 2019; Wojcik, 2019). Importantly, unlike traditional referent-selection paradigms in which children identify a referent immediately after hearing a novel label, all teaching trials in the present study were completed before testing began. Children were therefore required to retrieve previously learned label–object mappings across multiple test trials following the learning episode.

We have now revised the manuscript to clarify the distinction between referent selection and immediate retention of label-object mappings (Introduction, p.4, lines 79-87 and p.10, lines 225-231), and have refined our terminology throughout.

We also acknowledge that, because the study did not include an immediate referent selection measure, we cannot definitively determine whether chance-level performance reflected failures of initial learning, retrieval, or a combination of both. We have, therefore, tempered our interpretation of the findings in the Discussion. Finally, we have revised the Introduction and Discussion to more clearly articulate the study's contribution as examining children's immediate retention of word–object associations rather than long-term retention following an extended delay.

5. One other question about the task is how distinguishable was the voice of the third speaker in the test phase from those of the first and second speakers in the teaching phase? If the voices could not be clearly distinguished, the children may have attributed the voice in the test phase to one of the previous speakers, which may have implications for their performance.

Response: We thank Reviewer 2 for this question. The test speaker was a different individual from the two speakers who participated in the teaching phase (now clarified in the Method section, (p.20, lines 478-480). However, because the degree of perceived similarity between the test speaker and the teaching speakers audio recordings were not formally assessed, we cannot completely rule out the possibility that some children may have associated the test speaker's voice with one of the speakers from the teaching phase. We have, therefore, noted this as a limitation of the study and include that future studies could introduce a third speaker visually during testing to make sure that children realize that this is a completely different speaker (see p.30-31, lines 727-730).

6. The results would be more appropriately interpreted as cues that do not violate established lexical knowledge allowed cross-situational statistics to override mutual exclusivity, regardless of speaker accuracy. And the reason for not observing the same in the Conflict-with-ME condition would be that the introduction of novel labels to objects that the children were overly familiar with confused them, leading to them not learning anything. Hence, the chance-level performance. In short, the authors need to take other cues into account when discussing literature and interpreting the results.

Response: We appreciate Reviewer 2’s suggestion about more carefully interpreting our findings, especially because children's performance may have been influenced by multiple sources of information available during learning. We have now revised the Discussion (p.28, lines 668-674) to acknowledge that successful performance in the Align-with-ME condition may reflect the combined influence of multiple cues, including mutual exclusivity expectations, repeated label–object pairings, and speaker testimony, rather than reliance on any single cue in isolation.

We also agree with the Reviewer that chance-level performance in the Conflict-with-ME condition may reflect the increased difficulty associated with learning novel labels for highly familiar objects. We have therefore expanded the Discussion to include this alternative interpretation and consider how competing lexical representations may have contributed to poorer performance in this condition. These revisions have been incorporated into the Discussion section (p.28-29, lines 677-679 and lines 684-689).

7. Regarding the one-sample tests, it appears to me that the reported tests were one-tailed, as the graph for the familiar object trials in the Align-with-ME condition seems to suggest that performance was significantly below chance, but the statistics suggest otherwise. Was there a rationale for conducting one-tailed tests? This has serious implications for the interpretation of results, because if the children performed significantly below chance, it means that they were showing preference for a different object than expected. This would demonstrate associations of ‘not the target’ has been formed, which would be evidence for statistical learning taking place. If this was the case, I wondered if the manipulation of speaker accuracy was relevant at all. It could be that the other cues (i.e., cross-situational statistics and their lexical knowledge) were more salient and the children relied on them solely and disregarded speaker accuracy completely. This would mean that the study did not actually test the effect of speaker accuracy and any claims about speaker accuracy would be unwarranted. In this case, the authors did not really test what they set out to investigate.

Response: We thank the Reviewer for this thoughtful comment. The one-sample Wilcoxon signed-rank tests were conducted as one-tailed tests because our hypotheses were directional and specifically predicted performance above chance rather than below chance. We now make this rationale explicit in the original manuscript in the Analyses section (p.22, lines 530-532).

However, the Reviewer’s point is well taken, and we conducted additional analysis to examine whether performance in all learning conditions differed significantly from chance using two-tailed tests. None of the conditions, specifically including the familiar-object testing, yielded performance values significantly below chance (all ps > .05). Therefore, we do not interpret the results as evidence of a systematic preference for the other object or as direct evidence that children formed "not-the-target" associations. We now add a footnote (see Reference list 71 ) on conducting a post-hoc two-tailed analysis to confirm chance performance across learning conditions.

Footnote - To address the possibility of below-chance performance, we conducted post-hoc two-tailed Wilcoxon signed-rank tests for all learning conditions. None of the conditions, specifically including familiar object testing differed significantly from chance performance (all ps > .05), indicating no evidence that children formed consistent non-target associations.

Minor Comments: Below are responses to substantial minor comments. All other suggestions and corrections have been incorporated into the manuscript. We have also tracked our revisions in the manuscript, highlighted in yellow.

8. It would be good to start with some background information.

Response: We have now revised this section.

9. (lines 28-33) The description of the design of the study could be made clearer and more succinct.

Response: We have now revised this section.

10. (lines 70-74) This sentence is utterly difficult to follow. Please check that the information here is accurate and that it follows logically from the previous sentence.

Response: We have now removed these lines and revised the paragraph.

11. (lines 101-104) The writing here could be clearer. I suggest explicitly comparing against accurate speakers here and replacing ‘correct’ with ‘intended’. Also, it should be made clear in the description of the study that the children were not tested on immediate referent selection, but a slightly delayed identification/recall.

Response: We have made the suggested changes (see p.5, lines 108-110).

12. (line 113) Please explain/elaborate on what is meant by ‘equally well’.

Response: We have revised this (see p.5, lines 117-122).

13. (lines 405-424) Please provide detailed information on the timings of event. How long were the objects shown before the word was spoken? How long did each trial last?

Response: We have now added this information under Procedure, specific to each phase in the experiment.

14. (line 481) Please provide clear justification for running separate GLMMs for the novel and familiar blocks.

Response: We have now added this information (see p.22-23, lines 540-544).

Reviewer #3

After reading the manuscript carefully, my main concerns primarily relate to a) the conceptual and theoretical integration of the research question within the existing literature, b) the clarity and strength of the study’s motivation and rationale, c) the structure of the methodological description, and d) the implementation and interpretation of their statistical analysis. I will explain each of these points, along with additional minor comments, in detail below.

Response: We thank Reviewer 3 for their thoughtful and constructive feedback on our manuscript. We have carefully revised the manuscript to provide a stronger theoretical foundation, improve the structure of the Method section, provide additional methodological details and justifications, and refine our interpretation of the findings.

1. I experienced the writing style of the abstract as somehow segmented and lacking a (short) conceptual introduction of the topic

Response: We have now added a brief conceptual introduction to the Abstract and revised it for better flow.

2. “The combined effect of speaker accuracy and mutual exclusivity (ME) was assessed” --> Could the authors define the effect of interest (effect on …?)

Response: We have now added “The present study examined the combined effect of speaker accuracy and mutual exclusivity (ME) on retention of label-object associations in 4-7-year-old English-speaking monolingual children.” to the abstract to indicate what was assessed.

3. The 2nd and 3rd sentence are redundant

Response: We have rectified this error.

4. The introduction moves rather quickly to empirical findings. It would benefit from a stronger conceptual and theoretical framing at the outset, including more background and a strong argumentation why the two focal cues (ME and speaker accuracy), and especially their combined effect, are theoretically important.

Response: We thank Reviewer 3 for this helpful suggestion. We agree that the Introduction would benefit from a stronger conceptual and theoretical framing. We have now reorganized the Introduction to begin with a broader multiple-cue account of word learning and strengthened the rationale for examining speaker accuracy and mutual exclusivity together. Specifically, we now emphasize that speaker accuracy and mutual exclusivity represent distinct sources of information that children must coordinate during real-world word learning. We have also streamlined the presentation of previous empirical work and more clearly articulated the theoretical importance of understanding how these cues jointly influence children's learning.

5. Further, the introduction would benefit from a clearer argumentation for the study’s rationale, by highlighting the novel contribution. In particular, if the main aspect of interest is the focus on children’s retention, as opposed to their immediate referent selection, this argumentation needs to be clearer, including the theoretical importance of assessing retention performance, as well as concrete differences to prior studies (e.g., p. 9 “However, this study assessed children’s immediate referent selection rather than the retention” – The authors may want to specify in how far this immediate referent selection differs from the immediate retention in the present study?)

6. p. 9: If the distinction between disambiguation and retention is of particular interest for the current study, and since retention is mentioned much earlier, introducing this contrast earlier in the manuscript (and clarifying its theoretical implications and relevance) would improve the structure and clarity of the study motivation.

Response: We thank Reviewer 3 for the valuable suggestions in numbered points 5 and 6, and we respond to these together here.

Indeed, the distinction between immediate referent selection (i.e., disambiguation) and retention of word–object mappings requires a clearer articulation in the Introduction. We note that a similar concern was raised by Reviewer 2, and we have collectively addressed this issue throughout the revised manuscript. Specifically, we have introduced this distinction earlier in the paper (Introduction, p.4, lines 79-87) and have expanded our rationale for focusing on retention as an outcome of word learning.

We now clarify that immediate referent selection reflects children's ability to identify a potential referent at the moment of learning, whereas retention provides information about the stability of newly formed word–object associations beyond the initial mapping event. We also discuss the theoretical importance of examining retention in the current study and highlight how this contribution differs from prior studies that primarily assessed immediate referent selection. These revisions have been incorporated into the Introduction (p.10, 224-230), where we more explicitly motivate the study's novel contribution and clarify the relevance of assessing retention performance.

7. p. 5 “While children’s looking patterns differed as a function of speaker accuracy” --> How did they differ?

Response: We have now added this information (see p.5, lines 107-112).

8. p. 7 (reference to Kalashnikova et al., 2016): From the current description, it is not entirely clear what the study is intended to illustrate in this context. If the goal of this paragraph is to show that children sometimes suspend ME, there is a substantial body of literature that could support this point more directly: e.g., work by Diesendruck shows that children avoid the ME conclusion if the speaker uses proper nouns instead of conventional words (Diesendruck, 2005), if the speaker doesn’t know the conventional word because they don’t speak the language in question (Diesendruck, 2005) or because they proved to be an inaccurate language user (Diesendruck et al., 2010). This line of work seems highly relevant to the current study and could be incorporated here or elsewhere in the manuscript.

Response: We greatly appreciate Reviewer 3 for the suggestion to include studies by Diesendruck and colleagues to highlight more directly children’s variable adherence to mutual exclusivity. Accordingly, we have revised our discussion of mutual exclusivity and replaced the Kalashnikova et al. (2016) example with findings from Diesendruck (2005) and Diesendruck et al. (2010), which demonstrate that children can suspend mutual exclusivity depending on a speaker's knowledge, conventional language use, and communicative context. These revisions strengthen the theoretical connection between mutual exclusivity and speaker characteristics and are now reflected on p.7 lines 165–170 in the Introduction.

9. p. 10: The section on the “combined impact of speaker accuracy and mutual exclusivity” would benefit from a clearer articulation of what remains unsolved by the current literature to motivate the study.

Response: We thank Reviewer 3 for the suggestion to provide further clarification on the rationale for examining the combined effects of speaker accuracy and mutual exclusivity. In the revised manuscript, we have strengthened the “Combined impact of speaker accuracy and mutual exclusivity” section in the Introduction by more explicitly identifying the gap in the existing literature. Specifically, we now highlight that prior studies have primarily examined the interaction between these cues using immediate referent selection paradigms, leaving open the question of how speaker accuracy and mutual exclusivity jointly influence the retention of newly learned word–object associations. We have also strengthened the transition from review of prior work to motivation for the present study, thereby clarifying the study's novel contribution (p.11, lines 256-259).

10. p. 11: “In the first block (novel object testing), children were presented with two novel objects …”: Here and in the following sentence, could the authors specify that these refer to the previously presented novel objects and words?

Response: We thank the Reviewer for this question. We have revised this section to clarify that the novel objects, familiar objects, and novel labels presented during testing were the same items that had been introduced during the Teaching phase (see p.12, lines 274-280).

11. The formatting and hierarchy of section headers seem inconsistent and might need revision (e.g., “Stimuli” as sub-header of “Measures”; “Recordings” at the same level as “Stimuli”; “Design” as sub-header of “Recordings” & at the same level as the components of the study; no “Procedure”, etc.) Further, the ordering of sections seems rather unintuitive and would benefit from a clearer structure (e.g., presenting the Design and Procedure before the Measures would make the manuscript easier to follow; the “Experimental task” is currently listed under Measures (p.14) – it would be more intuitive to describe this information directly before the procedure (i.e., the single components) are described).

Response: We have reorganized the Method section to improve the structure and flow of the experimental task and other measures used in the study.

12. In general, from the description of the procedure and design details, it was challenging to reconstruct the design fully, including the number of trials per condition, session etc., as relevant information is distributed across sections. Providing a clearer overview of the design (both in-text and potentially visually) would greatly improve readability.

Response: We have reorganized the Method section to improve the structure and flow of the experimental task and other measures used in the study. In addition, we have revised Figures 2, 3, and 4 to use consistent objects and labels across figures and have added information about the number of trials included in each phase of the task within each session to improve clarity and readability.

13. Relatedly, Figures 3 & 4: It would be helpful if the figures illustrated a complete sequence of example trials using consistent stimuli (same objects/ words across figures). This would make it easier to see how different trial types are constituted and relate to each other.

Response: We have now added this information to Figures 3 and 4 (pages 18,20 and 22).

14. Figure 3: Could the authors specify whether the information about the number of labels refers to both sessions or only one (and possibly add a similar information to the test trials/ Figure 4)?

Response: We have now added this information.

15. p. 17/18 “Using a non-social attentional cue ensured that any learning differences could be attributed to children’s weighting of speaker accuracy versus ME, rather than to their sensitivity to eye gaze or ostensive signals” --> Could the authors clarify this argument? It is not immediately obvious to me why this decision would produce less differences between tasks than, e.g., the consistent use of speaker pointing/ looking at one object.

Response: We appreciate the opportunity to clarify our rationale for using a non-social attentional cue during the Teaching phase. Our goal was to minimize children's reliance on highly salient social-referential cues, such as eye gaze or pointing, which could independently support label–object mappings. By using a simple motion cue to indicate the intended referent, we aimed to more directly examine children's use of speaker accuracy and mutual exclusivity during word learning.

16. p.19: “This resulted in 8 trials per testing block”: Per block or per condition/ session? A more explicit overview of the procedure earlier in the section would probably help clarify this as well.

Response: We have revised the description of the testing phase accordingly. The sentence now clarifies that each of the four novel labels was tested twice, once in each of the two testing blocks, resulting in eight trials per testing block (p.20, lines 492-495).

17. What was the reasoning behind deciding against a delay between the learning and test phase (although retention strictly refers to retrieving information from the long-term memory)?

Response: We appreciate the opportunity to clarify this point. In the revised manuscript (Introduction, p.4, lines 79-87 and p.10, lines 225-231) we now more clearly distinguish between immediate referent selection and retention of word–object mappings and explicitly discuss word learning as a multi-stage process. We also clarify that our intention was not to make claims about long-term retention or retrieval from long-term memory, but rather to examine children's ability to retain newly learned word–object associations across multiple trials following completion of the learning phase. In addition, we now acknowledge in the Discussion (p.33-34, lines 805-811) that the relatively short interval between teaching and testing differs from delayed retention paradigms commonly used to assess long-term memory and discuss this as a limitation of the current study.

18. Why did the novel object test block always precede the familiar object test block?

Response: We thank Reviewer 3 for this question. The Novel Object Test Block always preceded the Familiar Object Test Block because retention of novel label–novel object mappings represented the primary outcome of interest. In addition, novel-object testing provided the most direct assessment of children's learning, as the novel objects did not already have established labels. In this block, children were asked to identify novel objects associated with newly learned labels, whereas the Familiar Object Test Block required children to consider whether a novel label could refer to an object for which they already knew a conventional label. We anticipated that beginning with the novel-object trials would reduce potential confusion and help establish the task demands before children encountered the more challenging familiar-object trials. However, the fixed testing order may have affected performance, particularly in the Familiar Object Test Block. We have, therefore, revised the Discussion (p.31, lines737-746) to acknowledge testing order as a limitation of the current design and to suggest that future studies should consider alternative testing formats that allow novel and familiar referents to be assessed within a single testing phase and use implicit measures of word learning (e.g., eye-tracking).

19. Was there a reason to separate novel and familiar object testing into two blocks, rather than presenting them within a single trial (with 4 objects presented)? As currently implemented, it may suggest to participants that the same word should refer to both a novel object (in the novel object testing) as well as a familiar object (in the familiar object testing)?

Response: Reviewer 3 raises an excellent question. Novel-object and familiar-object testing were separated into two blocks to independently assess whether children had a preference to retain novel labels for novel objects or familiar object. This would not have been possible if all four objects were presented in a single trial. We also anticipated children in this age range getting confused when making explicit button-press responses for four choices. Nonetheless, presenting the same novel labels across both testing blocks may have influenced children's interpretation of the task and encouraged consideration of multiple potential referents. We have, therefore, added this as a limitation of the current design and discuss how future studies may address this issue using alternative testing formats and more implicit measures of word learning in the Discussion (p.31, lines741-743).

20. Table 1: The ordering of indices should be checked.

Response: We have now rectified this error.

21. In the description of the GLMM, could the authors a) add the inclusion of the interaction term as a fixed effect to the description and b) specify which kind of GLMM has been performed (e.g., binomial error distribution)?

Response: We have now added this information (see p.22, lines 540-541)

22. As a suggestion, while a Wilcoxon test is suitable for this kind of data, the same information could be inferred from a GLMM (i.e., using a consistent approach): e.g., When predicting the outcome by a four-level factor (encompassing the 4 levels accurate-align, inaccurate-align, accurate-conflict, inaccurate-conflict) and removing the intercept, the output would directly reveal the results of testing each of these groups against chance (50%).

Response: We thank the reviewer for this helpful suggestion. We agree that performance relative to chance could also be examined within a GLMM framework using a four-level factor representing the four learning conditions. However, our primary GLMM analyses were designed to evaluate the factorial structure of the design, that is, the effects of speaker accuracy, learning condition, and their interaction at the trial level. In contrast, the Wilcoxon signed-rank tests addressed a separate question: whether performance within each individual learning condition differed from chance. We chose to retain this approach because it provides a straightforward and commonly used method for evaluating chance-level performance (Crespo & Kaushanskaya, 2021; Gangopadhyay & Kaushanskaya, 2021,2022; Wojcik, 2019) and complements the trial-level GLMM analyses.

23. My main concern in this section relates to the interpretation of the GLMM: Because the model includes an interaction term, the main effects are conditional on the value of the other predictor (i.e., they refer to the other predictor being 0). Given the chosen coding (centered predictors), the reported main effects reflect effects at the average level of the other predictor (i.e., at a very specific value of the other predictor), which is not equivalent to a global main effect. In the current form of the manuscript, this distinction is not made explicit, although it changes how the results are interpreted and might have implications for the study’s implications. If the goal is to test global main effects, these should be evaluated directly – either by fitting a second model without the interaction term (after finding it to be non-significant) and interpreting the main effects directly, or via model comparisons (models with vs. without the main effect of interest).

Response: We thank the Reviewer for this thoughtful observation. We agree that, when interaction terms are included in a model, the interpretation of fixed effects requires clarification. In the present analyses, we used contrast coding for speaker accuracy (Accurate = -0.5, Inaccurate = 0.5) and learning condition (Align-with-ME = -0.5, Conflict-with-ME = 0.5), such that the reported fixed-effect estimates represent effects evaluated at the average level of the other predictor (i.e., the main effect). The interaction between speaker accuracy and learning condition was retained because it was a theoretically motivated component of the planned model. Because the interaction between speaker accuracy and learning condition was not significant in either model, our substantive conclusions remained unchanged. However, to improve clarity on the analytical model, we have revised the Results section to explicitly describe the interpretation of the fixed effects under contrast coding (p. 24, lines 584-586 and p.25, lines 608-609)

24. Age as predictor: Could the authors clarify why age was excluded from the analyses due to non-significance (and not either kept as a control variable, or even kept as a variable that potentially interacts with the effects of interest)?

Response: We thank Reviewer 3 for this question. Given that the sample spanned ages 4–7 years, we evaluated the first model with Age(centered) as a covariate. Age did not significantly predict performance, did not improve model fit (AIC was lower for the models without Age), and did not alter the pattern of fixed effects. Therefore, we retained the simpler model without Age in the final analyses. We have clarified this rationale in the Results section.

25. Figures 5 & 6: It would be very nice and more informative to present the data at the level of individual participants (e.g., by showing single dots) rather than aggregated bars.

Response: We have now added data at the level of individual participants using scatter plots.

26. Further, as a suggestion, the authors could consider combining Figures 5 and 6 into a single (2x2) figure – since the conditions are later compared, presenting them jointly would facilitate visual comparison. And finally, it would be helpful to specify what the error bars represent (if they are retained).

Response: We have now combined Figures 5 and 6 as suggested and revised the figures to show data at the individual level.

27. p. 25 “Children’s below-chance performance”: Was the performance really below chance or rather “not above chance?

Response: We agree that the original wording was imprecise. We have revised the text to clarify that children's performance in this condition was not above chance.

28. p. 25 “…possible that children assigned the novel label to the familiar object instead”: If that was the case, wouldn’t we see that in their performance in the familiar object testing?

Response: We agree with the Reviewer that the original interpretation was not fully supported by the pattern of results, as children did not perform above chance during familiar-object testing in the Conflict-with-ME condition. We have, therefore, revised this section (see p.28, lines 684-689) to avoid suggesting that children systematically assigned the novel label to the familiar object. Instead, we now discuss the possibility that conflicting cues disrupted learning, resulting in uncertainty about the intended referent and a failure to establish stable word–object mappings.

29. p. 26 “older children, with more developed metacognitive and linguistic skills, may rely less on speaker accuracy”: Could the authors elaborate further, why it is plausible to expect an effect in this direction (although sensitivity to speaker reliability is generally rather assumed to increase with age)? Elaborate, why?

Response: We agree that the original wording could be interpreted as suggesting that sensitivity to speaker accuracy decreases with age, which was not our intention. We have revised the Discussion ( p.29, lines 711-718) to now discuss the possibility that older children may integrate speaker accuracy with other available cues, such as lexical expectations and repeated label–object pairings (to infer from cross-situational statistics), when forming label–object mappings. This interpretation is more consistent with the broader literature on selective trust and cue integration during word learning.

30. p. 27 “This ordering was intentional, as presenting familiar-object trials first could prime children …”: Could the authors explain why an interference in this (prevented) direction would be less worrying than in the other direction?

Response: We appreciate this thoughtful observation by Reviewer 3. Our rationale for presenting the Novel Object Test Block first was that it provided the more intuitive testing situation for children. We agree, however, that the fixed testing order may have influenced performance in the subsequent familiar-object block. We have, therefore, revised the Discussion to acknowledge this possibility and discuss testing order as a limitation of the current design (see p.31 lines 737-745).

Attachment Submitted filename: Response to Reviewers.docx

# Peer review: Decision Letter 1

**DOI:** 10.1371/journal.pone.0361483.r003

**Authors:** Jie Wang

11 Sep 2026

PONE-D-26-10165R1The role of speaker accuracy and mutual exclusivity in children’s novel word learningPLOS One

Dear Dr. Banerjee,

Thank you for submitting your manuscript to PLOS One. After careful consideration, we feel that it has merit but does not fully meet PLOS One’s publication criteria as it currently stands. Therefore, we invite you to submit a revised version of the manuscript that addresses the points raised during the review process.

Please submit your revised manuscript by Oct 26 2026 11:59PM. If you will need more time than this to complete your revisions, please reply to this message or contact the journal office at plosone@plos.org. When you're ready to submit your revision, log on to https://www.editorialmanager.com/pone/ and select the 'Submissions Needing Revision' folder to locate your manuscript file.

Please include the following items when submitting your revised manuscript:

- A letter that responds to each point raised by the academic editor and reviewer(s). You should upload this letter as a separate file labeled 'Response to Reviewers'.
- A marked-up copy of your manuscript that highlights changes made to the original version. You should upload this as a separate file labeled 'Revised Manuscript with Track Changes'.
- An unmarked version of your revised paper without tracked changes. You should upload this as a separate file labeled 'Manuscript'.

If you would like to make changes to your financial disclosure, please include your updated statement in your cover letter. Guidelines for resubmitting your figure files are available below the reviewer comments at the end of this letter.

If applicable, we recommend that you deposit your laboratory protocols in protocols.io to enhance the reproducibility of your results. Protocols.io assigns your protocol its own identifier (DOI) so that it can be cited independently in the future. For instructions see: https://journals.plos.org/plosone/s/submission-guidelines#loc-laboratory-protocols. Additionally, PLOS One offers an option for publishing peer-reviewed Lab Protocol articles, which describe protocols hosted on protocols.io. Read more information on sharing protocols at https://plos.org/protocols?utm_medium=editorial-email&utm_source=authorletters&utm_campaign=protocols.

As the corresponding author, your ORCID iD is verified in the submission system and will appear in the published article. PLOS supports the use of ORCID, and we encourage all coauthors to register for an ORCID iD and use it as well. Please encourage your coauthors to verify their ORCID iD within the submission system before final acceptance, as unverified ORCID iDs will not appear in the published article. *Only*  the individual author can complete the verification step; PLOS staff *cannot*  verify ORCID iDs on behalf of authors.

We look forward to receiving your revised manuscript.

Kind regards,

Jie Wang, Ph.D.

Academic Editor

PLOS One

Journal Requirements:

1. If the reviewer comments include a recommendation to cite specific previously published works, please review and evaluate these publications to determine whether they are relevant and should be cited. There is no requirement to cite these works unless the editor has indicated otherwise.

2. Please review your reference list to ensure that it is complete and correct. If you have cited papers that have been retracted, please include the rationale for doing so in the manuscript text, or remove these references and replace them with relevant current references. Any changes to the reference list should be mentioned in the rebuttal letter that accompanies your revised manuscript. If you need to cite a retracted article, indicate the article’s retracted status in the References list and also include a citation and full reference for the retraction notice.

Additional Editor Comments:

The authors should address the comments raised by the reviewers.

[Note: HTML markup is below. Please do not edit.]

Reviewer's Responses to Questions

**Comments to the Author**

1. If the authors have adequately addressed your comments raised in a previous round of review and you feel that this manuscript is now acceptable for publication, you may indicate that here to bypass the “Comments to the Author” section, enter your conflict of interest statement in the “Confidential to Editor” section, and submit your "Accept" recommendation.

Reviewer #1: All comments have been addressed

Reviewer #2: (No Response)

Reviewer #3: (No Response)

**********

2. Is the manuscript technically sound, and do the data support the conclusions?

The manuscript must describe a technically sound piece of scientific research with data that supports the conclusions. Experiments must have been conducted rigorously, with appropriate controls, replication, and sample sizes. The conclusions must be drawn appropriately based on the data presented.

Reviewer #1: Yes

Reviewer #2: Yes

Reviewer #3: Yes

**********

3. Has the statistical analysis been performed appropriately and rigorously?

Reviewer #1: Yes

Reviewer #2: Yes

Reviewer #3: Yes

**********

4. Have the authors made all data underlying the findings in their manuscript fully available?

The PLOS Data policy requires authors to make all data underlying the findings described in their manuscript fully available without restriction, with rare exception (please refer to the Data Availability Statement in the manuscript PDF file). The data should be provided as part of the manuscript or its supporting information, or deposited to a public repository. For example, in addition to summary statistics, the data points behind means, medians and variance measures should be available. If there are restrictions on publicly sharing data—e.g. participant privacy or use of data from a third party—those must be specified.

Reviewer #1: Yes

Reviewer #2: Yes

Reviewer #3: Yes

**********

5. Is the manuscript presented in an intelligible fashion and written in standard English?

PLOS ONE does not copyedit accepted manuscripts, so the language in submitted articles must be clear, correct, and unambiguous. Any typographical or grammatical errors should be corrected at revision, so please note any specific errors here.

Reviewer #1: Yes

Reviewer #2: Yes

Reviewer #3: Yes

**********

6. Review Comments to the Author

Please use the space provided to explain your answers to the questions above. You may also include additional comments for the author, including concerns about dual publication, research ethics, or publication ethics. (Please upload your review as an attachment if it exceeds 20,000 characters)

Reviewer #1: Title: The role of speaker accuracy and mutual exclusivity in children’s novel word learning

Overview

This study is examining the extent to which 4-to-7 year-old monolinguals weigh mutual exclusivity (ME) versus speaker testimony when learning novel words. Learners were exposed to four conditions: accurate speaker in align-with-ME, inaccurate speaker in align-with-ME, accurate speaker in conflict-with-ME, inaccurate speaker in conflict-with-ME. Findings suggest that children learned better when ME wasn’t violated, regardless of speaker accuracy history.

This is a novel and important contribution to the literature, and fits the scope of the journal. The introduction and the design are especially robust. I have a few comments on the analyses that should only require a minor revision.

Major comments

Analyses:

- Why not add item as a random effect as well since the analyses are said to be done at the trial level on line 481?

� The detailed response fully addressed my initial concern, thank you.

- Did you remove by-participant slopes because of convergence and singularity issues? If so, could you add a sentence to describe this?

� Same comment, here, thank you.

- Why is the estimate negative for the main effect of condition in the Novel object test trials GLMM? If Align with ME is coded as 0.5 and that learning was higher in that condition than in the Conflict with ME, shouldn’t the estimate be positive (and vice-versa for the next GLMM)?

� Thank you for checking this and editing the coding reporting.

- What was the rationale for having two sessions? Was it because this project is part of a larger one or was there a specific rationale? Either way, it would be helpful to specify this.

� Perfect, thank you for clarifying this.

- In the description of the testing phase, I am confused with these sentences: “Each novel label was tested twice, once in each of the two blocks. This resulted in 8 trials per testing block.” Aren’t there only 2 blocks, novel object block and a familiar object block? Therefore, “tested once in each block” would mean 4 trials across both sessions, not 8? Perhaps, the sentence should be corrected to “Each of the 4 novel labels (accurate or inaccurate, in align-with ME or conflict-with ME) was tested twice, once in each of the two per blocks. This resulted in 8 trials per testing block.”

� Same comment, thank you.

Minor comments

Thank you for addressing all the comments in this section, I have no further comments.

Reviewer #2: The manuscript is a revised version of a manuscript I reviewed earlier for this journal. My main concerns pertained to the structure of the Introduction, the discussion of the theoretical framework, the design of the study, the one-sample tests, and the interpretation of results. The authors have substantially revised the manuscript to address many of my concerns to satisfaction and clarified several things that were unclear in the previous version. Overall, the manuscript has improved in readability, clarity, and coherence. I only have the following, mostly minor, comments/suggestions.

Abstract

1. (line 32) “monolingual English speaking” would be more conventional and in line with other instances in the manuscript.

Introduction

2. (line 55) The acronym “ME” is defined here, please do not define this again later on. Also, please replace all subsequent mention of “mutual exclusivity” with the acronym.

3. (lines 61-62) “In contrast” here does not really make sense, please replace it with another adverbial.

4. (line 85) I find the use of “immediately” here problematic and inaccurate. Perhaps replace it with “following a short delay”?

5. (line 87) Here and in the rest of the manuscript, please qualify “retention” with “short-term” for clarity – to avoid confusion with the typical use of the word to refer to longer-term retention in the literature. In some instances (e.g., line 117), this would mean replacing “immediate” with “short-term”.

6. (line 119) Please clarify what “looking patterns were faster” means. Was it quicker fixation to the target object or more frequent switching between target and distractor objects?

7. (line 128) Please replace “only” with something along the lines of “tended to”.

8. (lines 131-132) The “inconsistent findings” discussed here do not seem to follow from what was discussed in the previous paragraph(s). Please explain what was inconsistent in the literature.

9. (line 147) Some studies have shown that children can use ME to learn the meaning of words at earlier ages: Mather and Plunkett (2010) at 10 months and Halberda (2003) at 17 months [reference no. 16].

10. (line 149 and 154) Please replace “avoided” and “resisted” with something along the lines of “tended to avoid/resist”. In line 154, “interpreted” will also need to be replaced with “interpret” following the edit.

11. (lines 171-177) To help with the flow and reduce redundancy, I suggest replacing the first sentence in the subsequent section (lines 179-180) with this paragraph. Also, I suggest omitting “, in the service of word learning” in line 173 and inserting a citation of Hollich et al. (2000) [reference no. 3] in line 175.

12. (line 249) The writing “deciding which label-object associations to retain” sounds to me that the children have explicit control over which cues to use when these are typically conceptualised as implicit. Please rephrase for clarity.

Method

13. (lines 328 and 334) Please use the more conventional “inclusion/exclusion criteria” instead of “inclusionary/exclusionary criteria”.

14. (Table 1) Perhaps replace “Mean” in the column heading with M for consistency with SD. Please italicise statistical notations.

15. (line 366) Please add a comma after “phase”.

16. (line 367) Please replace “representing” with “in” for clarity.

Results

17. (line 551) Please use “24” instead of “twenty-four” for consistency.

18. (line 573) My understanding is that the journal does not permit footnotes, so please briefly summarise the content of the footnote here.

19. (line 593) Please insert “significant” before “main effect”.

20. (line 597) Should “weight” be “weigh”?

21. (Table 2) The numbers in the table do not match those presented in the main text. Also, please include information on random effects. It would also be helpful to present 95% confidence intervals for the fixed effects.

22. (line 617) Please insert “significant” before “main effect”.

23. (line 618) The p-value is rounded incorrectly – should be .15. However, please see my comment below and report all p-values to 3 decimal places.

24. (Table 3) Similar to Table 2, please include information on random effects and provide 95% confidence intervals for the fixed effects.

25. In general, please provide exact p-values to 3 decimal places whenever possible (i.e., except for < .001) and other test statistics to 2 decimal places.

Discussion

26. (line 683) Please omit the p-value.

27. (line 698) Please insert “significant” before “main effect” and omit the p-value.

28. (lines 794-718) The discussion here seems to point towards an age by speaker accuracy effect, which could have been included in the GLMM analyses. What I suggest here is to say, if accurate, that the current sample did not provide sufficient power to test this interaction and then suggest this for future work.

29. (lines 727-730) This newly added information breaks the flow of the paragraph. I suggest moving this to the end of the paragraph, and instead of “recognize”, say “further note”.

30. (line 746) Please add brief information on what implicit measures of word learning can show us.

Reviewer #3: (see attachment)

I was involved in both the first and second rounds of review and was very pleased to see how substantially the manuscript has improved in response to the thoughtful revisions made by the authors and the suggestions raised during the first round of review. In particular, I found the revised Introduction much clearer and well structured. The addition of a conceptual introduction and the more explicit formulation of the main focus and research gap considerably strengthen the motivation for the study. The revised Method section has also benefited substantially from the new structure and figures, which make the procedure much easier to follow.

The majority of the points I raised in the first round have been addressed satisfactorily. I have a smaller number of remaining comments, most of which are minor and concern points that either emerged while reading the revised manuscript or that concern a clarification of comments I raised in the first round. I outline these points below.

1. Abstract:

- The revised abstract is much clearer and, in my view, now provides a good overview of the study. One point that remained somewhat unclear to me concerns the final conclusion (“that the relative weighting of these cues may vary depending on the learning context”) – could the authors clarify how this conclusion follows from the reported findings?

2. Introduction:

- The revised manuscript now makes the focus on children’s (short-term) retention across different learning contexts much clearer. I noticed, however, a slight asymmetry in the literature reviewed: The section on speaker accuracy discusses previous findings concerning both referent selection and retention, whereas the section on ME appears to focus primarily on children’s referent selection. The authors may decide whether they prefer to retain this focus, or whether they want to mention previous empirical findings on retention of word-object mappings established through ME in this section.

3. Method

- p. 22: I think the reading flow might be slightly improved if the model itself were introduced and explained first, followed by the explanation of why other random effects were not included.

4. Results

- One remaining point concerns the explanation of the GLMM coefficients at the beginning of the Results section (based on a comment I raised in the first round). I appreciate that the authors have added a clarification in response to my previous comment. However, I think the current wording could still be made somewhat clearer.

o The authors state that “Because speaker accuracy and learning condition were contrast coded (-0.5, 0.5), fixed-effects estimates are interpreted at the average level of the other predictor. Thus, the reported effects reflect average differences across levels of the other factor rather than effects at a specific reference category”. I have two concerns with the phrasing of these sentences:

� This interpretation does not concern all “fixed-effects estimates”/ “the reported effects”, but specifically the lower-order terms for condition and speaker accuracy, as these predictors are also included in the interaction.

� Further, referring to the “average level” of a categorical factor might be somewhat confusing, as there is no actual intermediate level between, e.g., an accurate and an inaccurate speaker (instead, the effect of, e.g., condition is the average of the effect of condition for accurate speakers and the effect of condition for inaccurate speakers). Therefore, it would be clearer to explain directly that, with the chosen contrast coding, the reported effect of condition represents the difference between conditions averaged equally across the two levels of speaker accuracy – and vice versa for the effect of speaker accuracy.

(This distinction is particularly relevant if the numbers of observations across the two levels are not equal, because the model coefficient then does not correspond to simply collapsing the observed data across speaker accuracy.)

- I noticed that some of the numerical values reported in the main text of the Results section are not identical to the ones reported in Table 2.

- In response to one of my comments from the first round, the authors state that “We have now combined Figures 5 and 6 as suggested”. In the revised manuscript, however, the two figures still appear separately. I was wondering whether an outdated version of the figure has been uploaded, or alternatively whether the response letter was not updated to reflect the final revision?

- Finally, in my previous review I suggested specifying in the captions of Figures 5 and 6 what the error bars represent (e.g., SE, SD, 95% confidence intervals etc.). This information still appears to be missing. Although this is a minor point, I think it would be useful to add so that the figures can be interpreted unambiguously.

5. Discussion

- The authors have clearly made an effort to incorporate the additional discussion points raised during the first round of review. I found these additions very valuable. In a few places, however, the transitions between the original discussion and newly added material felt somewhat abrupt.

o E.g., p. 30: “some children associated the test speaker’s voice with one of the speakers from the teaching phase” – the transition to this point could perhaps be made a little more fluent, and it might be helpful to briefly spell out the implication of this possibility.

o E.g., p. 33/34: the part beginning with “It is also important to note …” might work better as a separate paragraph. At present, both the transition into this point and the transition from it to the concluding sentence felt somewhat abrupt.

6. Reference list

- Reference 71 appears to contain a short paragraph that may have been intended as a footnote?

**********

7. PLOS authors have the option to publish the peer review history of their article (what does this mean?). If published, this will include your full peer review and any attached files.

If you choose “no”, your identity will remain anonymous but your review may still be made public.

**Do you want your identity to be public for this peer review?**  For information about this choice, including consent withdrawal, please see our Privacy Policy.

Reviewer #1: No

Reviewer #2: **Yes:**  Kin Chung Jacky Chan

Reviewer #3: No

**********

[NOTE: If reviewer comments were submitted as an attachment file, they will be attached to this email and accessible via the submission site. Please log into your account, locate the manuscript record, and check for the action link "View Attachments". If this link does not appear, there are no attachment files.]

To ensure your figures meet our technical requirements, please review our figure guidelines: https://journals.plos.org/plosone/s/figures

You may also use PLOS’s free figure tool, NAAS, to help you prepare publication quality figures: https://journals.plos.org/plosone/s/figures#loc-tools-for-figure-preparation.

NAAS will assess whether your figures meet our technical requirements by comparing each figure against our figure specifications.

Attachment Submitted filename: PLOS_082326_R2.docx

Attachment Submitted filename: Review_2.pdf

# Author comment: Author response to Decision Letter 2

**DOI:** 10.1371/journal.pone.0361483.r004

18 Sep 2026

We thank the Editor, the two anonymous reviewers and Dr. Chan for their helpful suggestions on the improvement of our manuscript PONE-D-26-10165R1 “The role of speaker accuracy and mutual exclusivity in children’s novel word learning” Below is a point-by-point response (in italics) of all the changes we have made to the manuscript as a result of the comments and the suggestions made by the Editor and the Reviewers. We have also tracked our revisions in the manuscript, highlighted in yellow.

Reviewer #1

Thank you for addressing all the comments in this section, I have no further comments.

Response: We appreciate the Reviewer’s acknowledgment that our responses and revisions have addressed their previous comments and concerns. We thank the Reviewer for their helpful feedback throughout the review process.

Reviewer #2 Dr. Chan

The authors have substantially revised the manuscript to address many of my concerns to satisfaction and clarified several things that were unclear in the previous version. Overall, the manuscript has improved in readability, clarity, and coherence. I only have the following, mostly minor, comments/suggestions.

Response: We are grateful to Dr Chan for their careful review of the revised manuscript and are happy that our revisions addressed many of their previous concerns. We also thank Dr Chan for the additional comments and suggestions to further improve the manuscript. We have addressed each of these comments below.

Below are responses to substantial minor comments. All other editorial suggestions and corrections have been incorporated into the manuscript, highlighted in yellow.

1. (line 55) The acronym “ME” is defined here, please do not define this again later on. Also, please replace all subsequent mention of “mutual exclusivity” with the acronym.

Response: We have made the suggested changes throughout the manuscript.

2. (lines 61-62) “In contrast” here does not really make sense, please replace it with another adverbial.

Response: We have replaced “In contrast” with “On the other hand” as suggested (see p. 3, lines 61–62).

3. (line 85) I find the use of “immediately” here problematic and inaccurate. Perhaps replace it with “following a short delay”?

Response: We have replaced “immediately” with “following a short delay” as suggested (see p. 4, line 85).

4. (line 87) Here and in the rest of the manuscript, please qualify “retention” with “short-term” for clarity – to avoid confusion with the typical use of the word to refer to longer-term retention in the literature. In some instances (e.g., line 117), this would mean replacing “immediate” with “short-term”.

Response: We have incorporated the term “short-term retention” throughout the manuscript where appropriate.

5. (line 119) Please clarify what “looking patterns were faster” means. Was it quicker fixation to the target object or more frequent switching between target and distractor objects?

Response: We have clarified that “faster looking patterns” referred to quicker fixation to the target object (see p. 5, line 121).

6. (lines 131-132) The “inconsistent findings” discussed here do not seem to follow from what was discussed in the previous paragraph(s). Please explain what was inconsistent in the literature.

Response: We have revised this section to clarify the mixed findings in the literature. Specifically, we now note that some studies have found a preference for accurate speakers during referent selection and retention, whereas others have found that children can learn from both accurate and inaccurate speakers, with differences instead emerging in the speed of looking toward labeled objects (see p. 6, lines 132–136).

7. (line 147) Some studies have shown that children can use ME to learn the meaning of words at earlier ages: Mather and Plunkett (2010) at 10 months and Halberda (2003) at 17 months [reference no. 16].

Response: We have replaced the previous statement with the following: “Some studies have shown that children can use ME to learn the meaning of words at earlier ages: Mather and Plunkett (2010) at 10 months and Halberda (2003) at 17 months.” as suggested (see p. 7, lines 150–151).

8. (lines 171-177) To help with the flow and reduce redundancy, I suggest replacing the first sentence in the subsequent section (lines 179-180) with this paragraph. Also, I suggest omitting “, in the service of word learning” in line 173 and inserting a citation of Hollich et al. (2000) [reference no. 3] in line 175.

Response: We have made the suggested revisions (see p. 8, lines 176–181).

9. (line 249) The writing “deciding which label-object associations to retain” sounds to me that the children have explicit control over which cues to use when these are typically conceptualised as implicit. Please rephrase for clarity.

Response: We have rephrased this sentence to clarify that children form and retain label–object associations when integrating multiple sources of information, rather than explicitly deciding which associations to retain (see p. 11, lines 246–248).

10. (line 573) My understanding is that the journal does not permit footnotes, so please briefly summarise the content of the footnote here.

Response: We appreciate Dr. Chan for pointing this out. We have now added the information that was in the footnote to the main text (see p.24, lines 566-569).

11. (Table 2) The numbers in the table do not match those presented in the main text. Also, please include information on random effects. It would also be helpful to present 95% confidence intervals for the fixed effects.

Response: We thank Dr. Chan for catching this error. The numbers in the table now match those in the text. We have also added information on random effects and confidence intervals for the fixed effects.

12. (Table 3) Similar to Table 2, please include information on random effects and provide 95% confidence intervals for the fixed effects.

Response: We have now added this information.

13. In general, please provide exact p-values to 3 decimal places whenever possible (i.e., except for < .001) and other test statistics to 2 decimal places.

Response: We have now done so throughout the manuscript.

14. (lines 794-718) The discussion here seems to point towards an age by speaker accuracy effect, which could have been included in the GLMM analyses. What I suggest here is to say, if accurate, that the current sample did not provide sufficient power to test this interaction and then suggest this for future work.

Response: We thank Dr. Chan for this suggestion. We have now mentioned this limitation in the Discussion and noted that future studies with larger samples could directly examine whether the influence of speaker accuracy on word learning changes with age (see p. 30, lines 716–719). Consequently, we have removed “According to multiple-cue accounts of word learning, children weigh and combine information from a variety of sources, including speaker characteristics, lexical expectations, and distributional information available within the learning environment [42-43]. In the present study, repeated label–object pairings and children's existing lexical knowledge may have provided additional information that influenced learning outcomes alongside speaker accuracy.”

15. (lines 727-730) This newly added information breaks the flow of the paragraph. I suggest moving this to the end of the paragraph, and instead of “recognize”, say “further note”.

Response: We have revised this section as suggested (see p. 31, lines 737–740).

16. (line 746) Please add brief information on what implicit measures of word learning can show us.

Response: We have added brief explanation of what implicit measures of word learning can reveal (see p. 31, lines 744–748), namely, that implicit measures can provide insight into the real-time dynamics of children’s recognition of newly learned label–object associations.

Reviewer #3

The majority of the points I raised in the first round have been addressed satisfactorily. I have a smaller number of remaining comments, most of which are minor and concern points that either emerged while reading the revised manuscript or that concern a clarification of comments I raised in the first round. I outline these points below.

Response: We are grateful to Reviewer 3 for their careful review of the revised manuscript and for their positive comments on the improvements made to the manuscript. We also thank them for the additional comments and suggestions to further strengthen the manuscript. We have addressed each of these comments below.

1. The revised abstract is much clearer and, in my view, now provides a good overview of the study. One point that remained somewhat unclear to me concerns the final conclusion (“that the relative weighting of these cues may vary depending on the learning context”) – could the authors clarify how this conclusion follows from the reported findings?

Response: We have now clarified the conclusion of the Abstract to more directly reflect the reported findings (see p. 2, lines 45–47). We now specifically say that children’s short-term retention of label–object associations is supported when available cues align, irrespective of speaker accuracy status. These associations, however, become less robust when ME and speaker cues provide conflicting information.

2. The revised manuscript now makes the focus on children’s (short-term) retention across different learning contexts much clearer. I noticed, however, a slight asymmetry in the literature reviewed: The section on speaker accuracy discusses previous findings concerning both referent selection and retention, whereas the section on ME appears to focus primarily on children’s referent selection. The authors may decide whether they prefer to retain this focus, or whether they want to mention previous empirical findings on retention of word-object mappings established through ME in this section.

Response: We thank the Reviewer for noting this distinction. In the section on speaker accuracy, we discuss studies demonstrating mixed findings regarding children’s preference for accurate versus inaccurate speakers, which include evidence from both referent selection and short-term retention tasks. In the ME section, our primary goal was to establish ME as an important cue that children use during word learning and to highlight the flexibility with which this cue is applied across different learning contexts. We therefore retained the current focus of this section and discuss evidence concerning short-term retention in the subsequent section on the combined influence of speaker accuracy and ME, which is more directly relevant to the present study.

3. p. 22: I think the reading flow might be slightly improved if the model itself were introduced and explained first, followed by the explanation of why other random effects were not included.

Response: We have now introduced the model first, followed by information on the final model, as suggested (see p.22-23, lines 530-543).

4. One remaining point concerns the explanation of the GLMM coefficients at the beginning of the Results section (based on a comment I raised in the first round). I appreciate that the authors have added a clarification in response to my previous comment. However, I think the current wording could still be made somewhat clearer. The authors state that “Because speaker accuracy and learning condition were contrast coded (-0.5, 0.5), fixed-effects estimates are interpreted at the average level of the other predictor. Thus, the reported effects reflect average differences across levels of the other factor rather than effects at a specific reference category”. I have two concerns with the phrasing of these sentences:

a. This interpretation does not concern all “fixed-effects estimates”/ “the reported effects”, but specifically the lower-order terms for condition and speaker accuracy, as these predictors are also included in the interaction.

b. Further, referring to the “average level” of a categorical factor might be somewhat confusing, as there is no actual intermediate level between, e.g., an accurate and an inaccurate speaker (instead, the effect of, e.g., condition is the average of the effect of condition for accurate speakers and the effect of condition for inaccurate speakers). Therefore, it would be clearer to explain directly that, with the chosen contrast coding, the reported effect of condition represents the difference between conditions averaged equally across the two levels of speaker accuracy – and vice versa for the effect of speaker accuracy. (This distinction is particularly relevant if the numbers of observations across the two levels are not equal, because the model coefficient then does not correspond to simply collapsing the observed data across speaker accuracy.)

Response: We thank Reviewer 3 for the valuable suggestions in numbered points 4a and 4b, and we respond to these together here. We agree that our previous wording was imprecise in referring broadly to all fixed-effect estimates and to the “average level” of the other predictor. We have revised the text to clarify that this interpretation applies specifically to the lower-order effects of learning condition and speaker accuracy (see p.24, lines 584-588). We now state that, with the chosen contrast coding, the lower-order effect of learning condition represents the difference between conditions averaged equally across the two levels of speaker accuracy, and the lower-order effect of speaker accuracy represents the difference between speaker accuracy levels averaged equally across the two learning conditions.

5. I noticed that some of the numerical values reported in the main text of the Results section are not identical to the ones reported in Table 2.

Response: We thank the Reviewer for identifying this error, which was also caught by Dr. Chan (Reviewer 2). We have now ensured that values in the table match those in the main text.

6. In response to one of my comments from the first round, the authors state that “We have now combined Figures 5 and 6 as suggested”. In the revised manuscript, however, the two figures still appear separately. I was wondering whether an outdated version of the figure has been uploaded, or alternatively whether the response letter was not updated to reflect the final revision?

Response: We thank the Reviewer for noting this discrepancy and apologize for the misunderstanding. Indeed, an outdated version of the figure was previously uploaded. We have now combined Figures 5 and 6 into a single figure (Figure 5), as originally intended and indicated in our previous response.

7. Finally, in my previous review I suggested specifying in the captions of Figures 5 and 6 what the error bars represent (e.g., SE, SD, 95% confidence intervals etc.). This information still appears to be missing. Although this is a minor point, I think it would be useful to add so that the figures can be interpreted unambiguously.

Response: We thank the Reviewer for noting this. We have now clarified in the caption of the combined figure (Figure 5) that the error bars represent standard errors of the mean.

8. Discussion - The authors have clearly made an effort to incorporate the additional discussion points raised during the first round of review. I found these additions very valuable. In a few places, however, the transitions between the original discussion and newly added material felt somewhat abrupt.

a. E.g., p. 30: “some children associated the test speaker’s voice with one of the speakers from the teaching phase” – the transition to this point could perhaps be made a little more fluent, and it might be helpful to briefly spell out the implication of this possibility.

b. E.g., p. 33/34: the part beginning with “It is also important to note …” might work better as a separate paragraph. At present, both the transition into this point and the transition from it to the concluding sentence felt somewhat abrupt.

Response: We thank the Reviewer for these helpful suggestions (for 8a and 8b). We have revised the transitions and overall flow of the Discussion.

9. Reference 71 appears to contain a short paragraph that may have been intended as a footnote?

Response: We thank the Reviewer for noting this. This paragraph was initially intended as a footnote. We have instead incorporated this explanation into the main text, as recommended by Dr. Chan (Reviewer 2).

Attachment Submitted filename: Response_to_Reviewers_auresp_2.docx

# Peer review: Decision Letter 2

**DOI:** 10.1371/journal.pone.0361483.r005

**Authors:** Jie Wang

5 Oct 2026

<div>PONE-D-26-10165R2The role of speaker accuracy and mutual exclusivity in children’s novel word learningPLOS One

Dear Dr. Banerjee,

Thank you for submitting your manuscript to PLOS One. After careful consideration, we feel that it has merit but does not fully meet PLOS One’s publication criteria as it currently stands. Therefore, we invite you to submit a revised version of the manuscript that addresses the points raised during the review process.

Please submit your revised manuscript by Nov 19 2026 11:59PM. If you will need more time than this to complete your revisions, please reply to this message or contact the journal office at plosone@plos.org. When you're ready to submit your revision, log on to https://www.editorialmanager.com/pone/ and select the 'Submissions Needing Revision' folder to locate your manuscript file.

Please include the following items when submitting your revised manuscript:

- A letter that responds to each point raised by the academic editor and reviewer(s). You should upload this letter as a separate file labeled 'Response to Reviewers'.
- A marked-up copy of your manuscript that highlights changes made to the original version. You should upload this as a separate file labeled 'Revised Manuscript with Track Changes'.
- An unmarked version of your revised paper without tracked changes. You should upload this as a separate file labeled 'Manuscript'.

If you would like to make changes to your financial disclosure, please include your updated statement in your cover letter. Guidelines for resubmitting your figure files are available below the reviewer comments at the end of this letter.

If applicable, we recommend that you deposit your laboratory protocols in protocols.io to enhance the reproducibility of your results. Protocols.io assigns your protocol its own identifier (DOI) so that it can be cited independently in the future. For instructions see: https://journals.plos.org/plosone/s/submission-guidelines#loc-laboratory-protocols. Additionally, PLOS One offers an option for publishing peer-reviewed Lab Protocol articles, which describe protocols hosted on protocols.io. Read more information on sharing protocols at https://plos.org/protocols?utm_medium=editorial-email&utm_source=authorletters&utm_campaign=protocols.

As the corresponding author, your ORCID iD is verified in the submission system and will appear in the published article. PLOS supports the use of ORCID, and we encourage all coauthors to register for an ORCID iD and use it as well. Please encourage your coauthors to verify their ORCID iD within the submission system before final acceptance, as unverified ORCID iDs will not appear in the published article. *Only*  the individual author can complete the verification step; PLOS staff *cannot*  verify ORCID iDs on behalf of authors.

We look forward to receiving your revised manuscript.

Kind regards,

Jie Wang, Ph.D.

Academic Editor

PLOS One

Journal Requirements:

If the reviewer comments include a recommendation to cite specific previously published works, please review and evaluate these publications to determine whether they are relevant and should be cited. There is no requirement to cite these works unless the editor has indicated otherwise.

Please review your reference list to ensure that it is complete and correct. If you have cited papers that have been retracted, please include the rationale for doing so in the manuscript text, or remove these references and replace them with relevant current references. Any changes to the reference list should be mentioned in the rebuttal letter that accompanies your revised manuscript. If you need to cite a retracted article, indicate the article’s retracted status in the References list and also include a citation and full reference for the retraction notice.

[Note: HTML markup is below. Please do not edit.]

Reviewers' comments:

Reviewer's Responses to Questions

**Comments to the Author**

1. If the authors have adequately addressed your comments raised in a previous round of review and you feel that this manuscript is now acceptable for publication, you may indicate that here to bypass the “Comments to the Author” section, enter your conflict of interest statement in the “Confidential to Editor” section, and submit your "Accept" recommendation.

Reviewer #2: (No Response)

Reviewer #3: (No Response)

**********

2. Is the manuscript technically sound, and do the data support the conclusions?

The manuscript must describe a technically sound piece of scientific research with data that supports the conclusions. Experiments must have been conducted rigorously, with appropriate controls, replication, and sample sizes. The conclusions must be drawn appropriately based on the data presented.

Reviewer #2: Yes

Reviewer #3: Yes

**********

3. Has the statistical analysis been performed appropriately and rigorously?

Reviewer #2: Yes

Reviewer #3: Yes

**********

4. Have the authors made all data underlying the findings in their manuscript fully available?

The PLOS Data policy requires authors to make all data underlying the findings described in their manuscript fully available without restriction, with rare exception (please refer to the Data Availability Statement in the manuscript PDF file). The data should be provided as part of the manuscript or its supporting information, or deposited to a public repository. For example, in addition to summary statistics, the data points behind means, medians and variance measures should be available. If there are restrictions on publicly sharing data—e.g. participant privacy or use of data from a third party—those must be specified.

Reviewer #2: Yes

Reviewer #3: Yes

**********

5. Is the manuscript presented in an intelligible fashion and written in standard English?

PLOS ONE does not copyedit accepted manuscripts, so the language in submitted articles must be clear, correct, and unambiguous. Any typographical or grammatical errors should be corrected at revision, so please note any specific errors here.

Reviewer #2: Yes

Reviewer #3: Yes

**********

6. Review Comments to the Author

Please use the space provided to explain your answers to the questions above. You may also include additional comments for the author, including concerns about dual publication, research ethics, or publication ethics. (Please upload your review as an attachment if it exceeds 20,000 characters)

Reviewer #2: Most of my comments from the previous round of review have been addressed to satisfaction. I only have a few very minor suggestions.

Introduction

1. (line 55) ‘ME’ should be spelt out in full here as it is the first time this acronym is used after the Abstract. Sorry if this was not clear in my previous comment.

2. (line 83) Here, I think it would be helpful to mention that some studies have looked at long-term retention (delay of 5-10 minutes), to highlight the need to study short-term retention.

3. (lines 150-151) This first sentence may be better suited in line 161, before “However”.

4. (line 187) Please use the past tense ‘provided’ instead.

5. (line 207) Please use the plural ‘speakers’.

6. (line 293) Full stop missing at the end of this line.

Method

7. (line 530) Wilcoxon signed-rank test is not descriptive. I would omit ‘descriptive analyses’ here to avoid confusion.

Results

8. Please follow APA conventions and be consistent in including leading zeroes in the main text and in the tables for Ms, SDs, bs, zs, ps, and SEs.

9. Please report exact p-values to 3 decimal places in the Chance performance analysis section.

10. (line 599) The SE reported here is still different from that in Table 2.

Discussion

11. (line 764) Please replace ‘effect’ with ‘affect’.

Reviewer #3: The authors have addressed the great majority of the points I raised in the previous round, and I think the revisions have clearly improved the manuscript in terms of readability and clarity. I only have two very minor remaining requests for clarification, both relating to comments from the previous round. Beyond these points, I have no further comments.

First, following the revisions, the GLMMs and their interpretation are now described very clearly, particularly on p. 24. On p. 25, in the sentence “As in the novel-object analyses, fixed-effect estimates are interpreted at the average level of the other predictor due to contrast coding,” the authors could simply clarify that this refers to the “lower-level fixed-effect estimates” or, more specifically, to the “fixed-effect estimates of condition and accuracy,” as they do on p. 24.

Second, Table 2 still contains one value that differs from the corresponding value reported in the text: the SE for the interaction term.

**********

7. PLOS authors have the option to publish the peer review history of their article (what does this mean?). If published, this will include your full peer review and any attached files.

If you choose “no”, your identity will remain anonymous but your review may still be made public.

**Do you want your identity to be public for this peer review?**  For information about this choice, including consent withdrawal, please see our Privacy Policy.

Reviewer #2: **Yes:**  Kin Chung Jacky Chan

Reviewer #3: No

**********

[NOTE: If reviewer comments were submitted as an attachment file, they will be attached to this email and accessible via the submission site. Please log into your account, locate the manuscript record, and check for the action link "View Attachments". If this link does not appear, there are no attachment files.]

To ensure your figures meet our technical requirements, please review our figure guidelines: https://journals.plos.org/plosone/s/figures

You may also use PLOS’s free figure tool, NAAS, to help you prepare publication quality figures: https://journals.plos.org/plosone/s/figures#loc-tools-for-figure-preparation.

NAAS will assess whether your figures meet our technical requirements by comparing each figure against our figure specifications.

# Author comment: Author response to Decision Letter 3

**DOI:** 10.1371/journal.pone.0361483.r006

6 Oct 2026

We thank the Dr. Chan and Reviewer 3 for their helpful suggestions on the improvement of our manuscript. Below is a point-by-point response (in italics) to all the comments and the suggestions made by the Reviewers. We have also tracked our revisions in the manuscript, highlighted in yellow.

Reviewer #2 Dr. Chan

Most of my comments from the previous round of review have been addressed to satisfaction. I only have a few very minor suggestions.

Response: We are grateful to Dr. Chan for their careful review of the revised manuscript and are happy that our revisions addressed most of their comments. We also thank Dr. Chan for the additional suggestions to further improve the manuscript.

1. (line 55) ‘ME’ should be spelt out in full here as it is the first time this acronym is used after the Abstract. Sorry if this was not clear in my previous comment.

Response: We have replaced “ME” by spelling it out as “mutual exclusivity (ME)” (p. 3, line 55), as this is the first time the acronym appears in the manuscript after the Abstract.

2. (line 83) Here, I think it would be helpful to mention that some studies have looked at long-term retention (delay of 5-10 minutes), to highlight the need to study short-term retention.

Response: We thank Dr Chan for this suggestion. We have now revised this section to note that some studies of word learning have examined retention following longer delays (Chan & Monaghan, 2025; Horst & Samuelson, 2008; Vlach & DeBrock, 2019), while highlighting the need to examine short-term retention when speaker accuracy and ME are considered together (p. 4, lines 83–85).

3. (lines 150-151) This first sentence may be better suited in line 161, before “However”.

Response: We have added “Some studies have shown that children can use ME to learn the meaning of words at earlier ages – Mather and Plunkett (2010) at 10 months and Halberda (2003) at 17 months”, before “however” as suggested (see p. 7, lines 162–163).

4. (line 187) Please use the past tense ‘provided’ instead.

Response: We have replaced “provide” with “provided” as suggested (see p.8, line 189).

5. (line 207) Please use the plural ‘speakers’.

Response: We have replaced “speaker” with “speakers” as suggested (see p.9, line 209).

6. (line 293) Full stop missing at the end of this line.

Response: We have added a period at the end of this line (see p.12, lines 295).

7. (line 530) Wilcoxon signed-rank test is not descriptive. I would omit ‘descriptive analyses’ here to avoid confusion.

Response: We have removed “descriptive” and now say “Following these analyses” (see p.22, lines 532).

8. Please follow APA conventions and be consistent in including leading zeroes in the main text and in the tables for Ms, SDs, bs, zs, ps, and SEs.

Response: We have revised the main text and tables to ensure that leading zeroes are reported consistently according to APA conventions (and exact p-values to three decimal places).

9. Please report exact p-values to 3 decimal places in the Chance performance analysis section.

Response: We have revised the Chance performance analysis section to report exact p-values to three decimal places.

10. (line 599) The SE reported here is still different from that in Table 2.

Response: We appreciate Dr. Chan for pointing this out. This was a typing error, and we have now corrected the SE in the main text to 0.42 to match Table 2.

11. (line 764) Please replace ‘effect’ with ‘affect’.

Response: We have replaced “effect” with “affect” as suggested (see p.32, line 768).

Reviewer #3

The authors have addressed the great majority of the points I raised in the previous round, and I think the revisions have clearly improved the manuscript in terms of readability and clarity. I only have two very minor remaining requests for clarification, both relating to comments from the previous round. Beyond these points, I have no further comments.

Response: We are grateful to Reviewer 3 for their careful review of the revised manuscript and for their positive comments on the improvements made to the manuscript. We also thank them for the additional suggestions to further strengthen the manuscript. We have addressed both their comments below.

1. Following the revisions, the GLMMs and their interpretation are now described very clearly, particularly on p. 24. On p. 25, in the sentence “As in the novel-object analyses, fixed-effect estimates are interpreted at the average level of the other predictor due to contrast coding,” the authors could simply clarify that this refers to the “lower-level fixed-effect estimates” or, more specifically, to the “fixed-effect estimates of condition and accuracy,” as they do on p. 24.

Response: We have revised the sentence as suggested to clarify that this interpretation refers specifically to the fixed-effect estimates of condition and accuracy (see p. 25, lines 611–613).

2. Table 2 still contains one value that differs from the corresponding value reported in the text: the SE for the interaction term.

Response: We thank the Reviewer for identifying this discrepancy, which was also noted by Dr. Chan (Reviewer 2). We have corrected the SE for the interaction term and ensured that the values in Table 2 match those reported in the main text.

Attachment Submitted filename: Response_to_Reviewers_auresp_3.docx

# Editor report: Decision Letter 3

**DOI:** 10.1371/journal.pone.0361483.r007

**Authors:** Jie Wang

12 Oct 2026

The role of speaker accuracy and mutual exclusivity in children’s novel word learning

PONE-D-26-10165R3

Dear Dr. Banerjee,

We’re pleased to inform you that your manuscript has been judged scientifically suitable for publication and will be formally accepted for publication once it meets all outstanding technical requirements.

Within one week, you’ll receive an e-mail detailing the required amendments. When these have been addressed, you’ll receive a formal acceptance letter and your manuscript will be scheduled for publication.

An invoice will be generated when your article is formally accepted. Please note, if your institution has a publishing partnership with PLOS and your article meets the relevant criteria, all or part of your publication costs will be covered. Please make sure your user information is up-to-date by logging into Editorial Manager at Editorial Manager® and clicking the ‘Update My Information' link at the top of the page. For questions related to billing, please contact billing support.

If your institution or institutions have a press office, please notify them about your upcoming paper to help maximize its impact. If they’ll be preparing press materials, please inform our press team as soon as possible -- no later than 48 hours after receiving the formal acceptance. Your manuscript will remain under strict press embargo until 2 pm Eastern Time on the date of publication. For more information, please contact onepress@plos.org.

Kind regards,

Jie Wang, Ph.D.

Academic Editor

PLOS One

Additional Editor Comments (optional):

Reviewers' comments:

# Editor report: Acceptance letter

**DOI:** 10.1371/journal.pone.0361483.r008

**Authors:** Jie Wang

PONE-D-26-10165R3

PLOS One

Dear Dr. Banerjee,

I'm pleased to inform you that your manuscript has been deemed suitable for publication in PLOS One. Congratulations! Your manuscript is now being handed over to our production team.

At this stage, our production department will prepare your paper for publication. This includes ensuring the following:

* All references, tables, and figures are properly cited

* All relevant supporting information is included in the manuscript submission,

* There are no issues that prevent the paper from being properly typeset

You will receive further instructions from the production team, including instructions on how to review your proof when it is ready. Please keep in mind that we are working through a large volume of accepted articles, so please give us a few days to review your paper and let you know the next and final steps.

Lastly, if your institution or institutions have a press office, please let them know about your upcoming paper now to help maximize its impact. If they'll be preparing press materials, please inform our press team within the next 48 hours. Your manuscript will remain under strict press embargo until 2 pm Eastern Time on the date of publication. For more information, please contact onepress@plos.org.

You will receive an invoice from PLOS for your publication fee after your manuscript has reached the completed accept phase. If you receive an email requesting payment before acceptance or for any other service, this may be a phishing scheme. Learn how to identify phishing emails and protect your accounts at https://explore.plos.org/phishing.

If we can help with anything else, please email us at customercare@plos.org.

Thank you for submitting your work to PLOS One and supporting open access.

Kind regards,

PLOS One Editorial Office Staff

on behalf of

Dr. Jie Wang

Academic Editor

PLOS One
